# Positioning and reversible suppression of CCR7^+^ dendritic cells in perivascular tumor niches shape cancer immunity

**DOI:** 10.1016/j.immuni.2025.11.020

**Published:** 2025-12-19

**Authors:** Beatrice Zitti, Florent Duval, Pratyaksha Wirapati, Mehdi Hicham, Yuxuan Xie, Juhyun Oh, Jan Hoelzl, Philippa Meiser, Marco Varrone, Hannah M. Peterson, Chiara Cianciaruso, Ruben Bill, Felix Bayerl, Evangelia Bolli, Anne-Gaëlle Goubet, Máté Kiss, Sheri McDowell, Phil Cheng, Dan Celestini, Julie Terzic, Thomas Zwahlen, Nagham Alouche, Nawel Zouggari, David Tarussio, Stephanie Tissot, Paula Nunes-Hasler, Mari Mino-Kenudson, Michael Lanuti, William C. Faquin, Peter M. Sadow, Jean-Christophe Tille, Sana Intidhar Labidi-Galy, Christopher S. Garris, Stephanie Hugues, Tatiana V. Petrova, Burkhard Ludewig, Sergio Quezada, Sanjiv Luther, Thorsten R. Mempel, Giovanni Ciriello, Sara I. Pai, Olivier Michielin, Jan P. Böttcher, Ralph Weissleder, Mikael J. Pittet

**Affiliations:** 1 Department of Pathology and Immunology, and Center for Translational Oncohaematology Research, University of Geneva, Geneva, Switzerland; 2 AGORA Cancer Research Center, and Swiss Cancer Center Leman, Lausanne, Switzerland; 3 Center for Systems Biology, Massachusetts General Brigham and Harvard Medical School, Boston, MA, USA; 4 Department of Medical Oncology, Heidelberg University Hospital, Heidelberg, Germany; 5 Institute of Molecular Immunology, TUM University Hospital, School of Medicine and Health, Technical University of Munich (TUM), Munich, Germany; 6 Department of Computational Biology, University of Lausanne, Lausanne, Switzerland; 7 Swiss Institute of Bioinformatics, Lausanne, Switzerland; 8 Department of Oncology, Geneva University Hospital, Geneva, Switzerland; 9 Department of Immunobiology, University of Lausanne, Lausanne, Switzerland; 10 Department of Oncology, Center for Experimental Therapeutics, Lausanne University Hospital (CHUV), Lausanne, Switzerland; 11 Department of Pathology, Massachusetts General Hospital and Harvard Medical School, Boston, MA, USA; 12 Division of Thoracic Surgery, Massachusetts General Hospital, Boston, MA, USA; 13 Department of Otolaryngology, Massachusetts Eye and Ear, Harvard Medical School, Boston, MA, USA; 14 Division of Clinical Pathology, Geneva University Hospitals, Geneva, Switzerland; 15 Department of Oncology, University of Lausanne, Lausanne, Switzerland; 16 Ludwig Institute for Cancer Research, Lausanne, Switzerland; 17 Institute of Immunobiology, Cantonal Hospital, St. Gallen, Switzerland; 18 University Heart Center, University Hospital Zurich, Zurich, Switzerland; 19 Center for Translational and Experimental Cardiology, University Hospital Zurich, Zurich, Switzerland; 20 Cancer Research UK Lung Cancer Centre of Excellence, University College London Cancer Institute, London, UK; 21 Center for Immunology and Inflammatory Diseases, Massachusetts General Hospital, Boston, MA, USA; 22 Department of Surgery, Yale University School of Medicine, New Haven, CT, USA; 23 Department of Experimental Immunology, Institute of Immunology, University of Tübingen, Tübingen, Germany; 24 M3 Research Center, University Hospital Tübingen, University of Tübingen, Tübingen, Germany; 25 Department of Systems Biology, Harvard Medical School, Boston, MA, USA; 26 Department of Radiology, Massachusetts General Hospital, and Harvard Medical School, Boston, MA, USA; 27 These authors contributed equally; 28 Present address: IFOM, The AIRC Institute of Molecular Oncology, Milan, Italy; 29 Lead contact

## Abstract

Tumor-resident CCR7^+^ dendritic cells (DCs) are key determinants of antitumor T cell responses. Here, we examined the localization of CCR7^+^ DCs within tumors and the impact of this positioning on antitumor immunity. Spatial, single-cell, and intravital analyses of human cancers and mouse models reveal that CCR7^+^ DCs form perivascular clusters. Fibroblasts surrounding venous blood vessels produced CCL19, guiding CCR7^+^ DCs into perivascular niches. Regulatory T (Treg) cells frequently contact perivascular CCR7^+^ DCs, suppressing CD40 expression and CD4^+^ and CD8^+^ T cell activation. Treg cell depletion restored CD40 expression by CCR7^+^ DCs, enhanced immunostimulatory programs, and improved T cell-dependent tumor control. Anti-PD-1 not only increased perivascular CCR7^+^ DC clustering and IL-12 production but also strengthened Treg-DC interactions through a CCL22-dependent mechanism, limiting therapeutic efficacy. CCR7^+^ DCs expressed both co-stimulatory and co-inhibitory molecules, which may underlie their capacity for antitumor activation and concurrent vulnerability to suppression. Modulating the mechanisms that form and restrain CCR7^+^ DC perivascular immune hubs may improve cancer immunotherapy.

## In brief

CCR7^+^ dendritic cells (DCs) are pivotal for antitumor immunity. Zitti, Duval et al. find that intratumoral CCR7^+^ DCs form perivascular hubs dependent on CCL19-CCR7 signaling. Regulatory T cells (Tregs) restrain immune function within these hubs, and both DC clustering and Treg cell suppression are modulated by anti-PD-1.

## Graphical Abstract

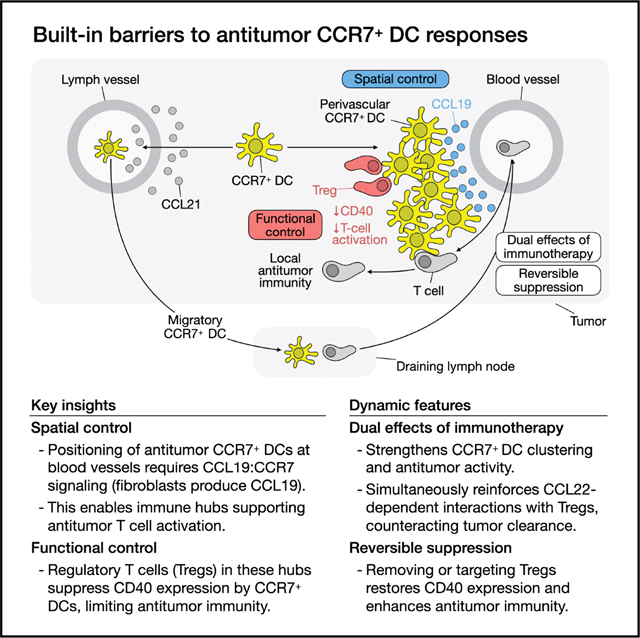

## INTRODUCTION

The cancer immunity cycle comprises a series of rate-limiting events that collectively drive antitumor immune responses.^1^ Conventional dendritic cells (cDCs) are critical to this cycle, as their presence and function in different tissues determine T cell activation and effector responses. Within tumor-draining lymph nodes, cDCs present tumor-derived antigens to naive T cells, initiating tumor-specific immunity and overcoming tolerance.^2–6^ Within tumors, cDCs sustain local effector T cell responses, as shown in mouse models.^7–21^ Human and mouse tumors lacking cDCs are typically poorly infiltrated by T cells and unresponsive to immunotherapy, whereas higher tumor cDC abundance is linked to stronger antitumor T cell responses and improved clinical outcomes.^1,8,10,22,23^ Thus, tumor cDCs are key determinants of antitumor immunity.

This study focuses on tumor-associated cDCs expressing the chemokine receptor CCR7, a hallmark of cDC activation in both cDC1s and cDC2s.^24,25^ CCR7^+^ DCs show enhanced antigen presentation, co-stimulatory factor expression, and cytokine production, promoting effector T cell activity.^20^ Single-cell RNA sequencing (scRNA-seq) studies initially identified the tumor CCR7^+^ DCs as DC3s,^26^ LAMP3^+^ DCs,^27^ or mregDCs.^28^ Despite differing nomenclature, tumor CCR7^+^ DCs are transcriptionally indistinguishable across studies,^29^ indicating conservation across cancer types and patients. The strong similarity between human and mouse CCR7^+^ DC phenotypes^26,29^ further supports the relevance of mouse models for their study.

In mouse tumors, CCR7^+^ DCs follow two main fates. Some migrate via lymphatic vessels (LVs) to tumor-draining lymph nodes, where they support T cell priming, and are termed migratory DCs.^30–32^ This migration depends on CCR7 sensing CCL21 gradients produced by lymphatic endothelial cells (LECs).^32–35^ Others remain within the tumor and accumulate near stromal blood vessels (BVs),^9,36^ hereafter referred to as perivascular CCR7^+^ DCs. These cells attract CXCR6^+^ effector T cells via CXCL16 and promote their survival and proliferation through interleukin-15 (IL-15) *trans*-presentation.^36^ Some perivascular CCR7^+^ DCs, likely cDC1-derived,^28^ produce IL-12, activating CD8^+^ T cells^9^ and natural killer (NK) cells^37^ to secrete interferon-γ (IFN-γ), which reinforces IL-12 production by CCR7^+^ DCs, establishing a type-I immunity feedback loop essential for antitumor responses and immunotherapy success.^9,21,38–41^ Consistently, higher CCR7^+^ DC abundance in human tumors is generally linked to better patient survival^26,42–45^ and improved immunotherapy outcomes.^23,46–48^ However, the mechanisms governing their perivascular localization and positioning in human tumors require investigation.

Moreover, the mere presence of CCR7^+^ DCs in tumors may not suffice for effective antitumor immunity, as their ability to deliver stimulatory signals likely determines effector T cell responses. Tumor CCR7^+^ DCs can express immunoregulatory genes such as *Cd274*, *Pdcd1lg2*, and *Cd200*, regulated by AXL Receptor Tyrosine Kinase (AXL) signaling and IL-4,^28^ suggesting that the tumor microenvironment (TME) actively modulates their function. Potential suppressors include regulatory T cells (Tregs),^49–53^ cytotoxic type 1 regulatory T cells,^54^ and macrophages.^55^ Tregs are of particular interest as they broadly suppress cDC stimulatory capacity in tumors^5,49,50,52^ and impair CCR7^+^ DC migration from lymphatic niches to lymph nodes,^56^ although their effect on perivascular CCR7^+^ DCs remains unclear.

Here, we comprehensively interrogated TMEs in various human cancers and mouse models to identify determinants of effective CCR7^+^ DC-mediated immunity, revealing perivascular positioning and local Treg regulation as critical variables in the cancer immunity cycle.

## RESULTS

### Perivascular CCR7^+^ DCs are conserved in human and mouse tumors

**Figure 1.**
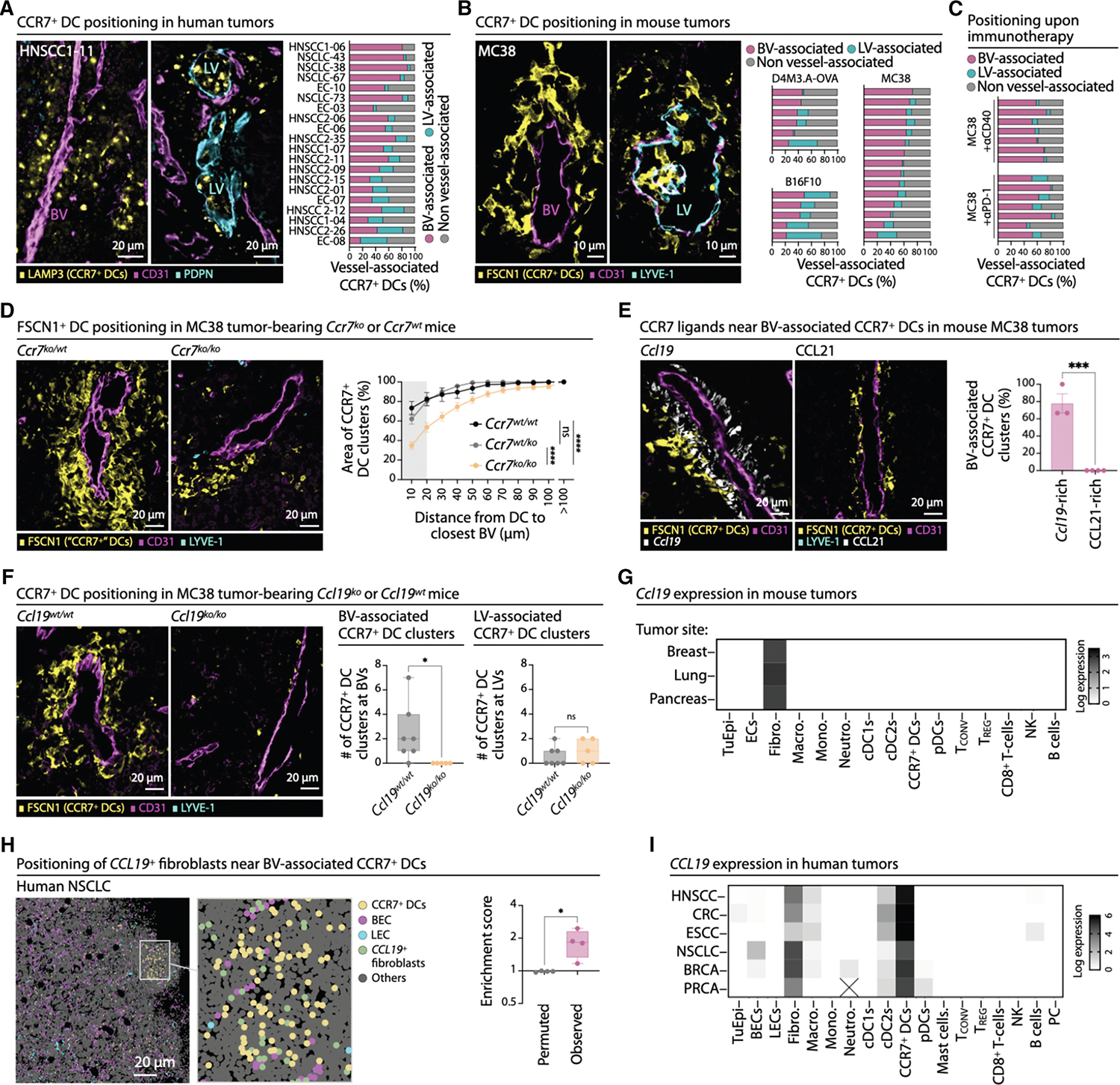
CCL19 anchors CCR7^+^ DCs to perivascular sites (A) Representative images and quantification of CCR7^+^ DCs (panCK^−^HLA-DR^+^LAMP3^+^, yellow) near BVs (CD31^+^PDPN^−^, magenta), or LVs (CD31^+^PDPN^+^, cyan) in human tumors (HNSCC, NSCLC, and EC). Scale bar represents 20 μm. Whole-tumor sections were analyzed for EC and NSCLC. Numbers of fields of view (FOVs) analyzed per HNSCC sample are as follows: HNSCC1–04 *n* = 7; HNSCC1–06 *n* = 16; HNSCC1–07 *n* = 11; HNSCC2–01 *n* = 126; HNSCC2–06 *n* = 455; HNSCC2–09 *n* = 180; HNSCC2–11 *n* = 122; HNSCC2–12 *n* = 79; HNSCC2–15 *n* = 205; HNSCC2–26 *n* = 293; HNSCC2–35 *n* = 175. One bar = one patient (Table S1). (B) Representative images and quantification of CCR7^+^ DCs (FSCN1^+^; yellow) located near BVs (CD31^+^LYVE-1^−^; magenta) or LVs (CD31^+^LYVE-1^+^; cyan) in mouse tumors (MC38, B16F10, and D4M3.A-OVA). Scale bar represents 10 μm. Whole-tumor sections were analyzed. One bar = one mouse. (C) Frequencies of BV-, LV- and non-vessel-associated CCR7^+^ DCs in mouse MC38 tumors 3 days post anti-CD40 or anti-PD-1 treatment. Whole-tumor sections were analyzed. One bar = one mouse. (D) (Left) Representative images of CCR7^+^ DCs (FSCN1^+^; yellow) located near BVs (CD31^+^LYVE-1^−^; magenta) in MC38 tumors inoculated in *Ccr7*^ko/wt^ and *Ccr7*^ko/ko^ mice, 3 days post anti-PD-1 treatment. (Right) Distribution of the area of CCR7^+^ DC surfaces in clusters relative to their distance to closest BVs and plotted as percentage of total CCR7^+^ DC cluster area. CCR7^+^ DC surfaces from clusters associated with LVs and those not in clusters were excluded from the analysis. Scale bar represents 20 μm. Whole-tumor sections were analyzed. One dot = average value of all clusters in each genotype (*Ccr7*^ko/ko^ *n* = 5 mice, 56 clusters; *Ccr7*^*wt*/ko^ *n* = 6 mice, 28 clusters; and *Ccr7*^*wt*/wt^ *n* = 3 mice, 19 clusters). Two-way ANOVA with multiple comparisons, mean with SEM; ****p < 0.0001 for comparison at 10 and 20 μm from closest BVs. (E) (Left) Representative images of CCR7^+^ DCs (FSCN1^+^; yellow) located near BVs (CD31^+^LYVE-1^−^; magenta) and *Ccl19* (*Ccl19*-eYFP^+^Tomato^+^; white) in *Ccl19*-ieYFP reporter mice (left image) or CCL21 (white, right image) in MC38 tumors. (Right) Frequencies of perivascular CCR7^+^ DC clusters associated with *Ccl19*-covered BVs or within CCL21^+^ areas of the tumors among total perivascular CCR7^+^ DC clusters. Scale bar represents 20 μm. Whole-tumor sections were analyzed. One dot = one mouse. Unpaired *t* test, mean with SEM; ***p < 0.001. (F) (Left) Representative images of CCR7^+^ DCs (FSCN1^+^; yellow) located near BVs (CD31^+^LYVE-1^−^; magenta) in MC38 tumors inoculated in *Ccl19*^wt/wt^ and *Ccl19*^ko/ko^ mice, 2 days post anti-PD-1treatment. (Right) Quantification of BV- or LV-associated CCR7^+^ DC clusters in MC38 tumors from *Ccl19*^wt/wt^ and *Ccl19*^ko/ko^ mice. Scale bar represents 20 μm. Whole-tumor sections were analyzed. One dot = one mouse, whiskers represent min to max. Unpaired *t* test; *p < 0.05. (G) Heatmap depicts log_2_-transformed averaged expression of *Ccl19* in indicated immune and non-immune populations in the TME of multiple mouse tumor models (breast,^57,58^ lung^59^ [and GSE201247], and pancreatic^60,61^). (H) (Left) Synthetic images of CCR7^+^ DCs (yellow), blood endothelial cells (BECs; magenta), lymphatic endothelial cells (LECs; cyan), and *CCL19*^+^ fibroblasts (green) in one representative NSCLC patient analyzed by spatial transcriptomics.^47^ (Right) Box plots depict the enrichment scores of *CCL19*^+^ fibroblasts within the neighborhood of BV-associated CCR7^+^ DCs, in four human NSCLC. Data are shown for both permuted (median enrichment scores from 1,000 permutations) and observed datasets. Scale bar represents 20 μm. Whole-tumor sections were analyzed. One dot = one sample. Paired *t* test, whiskers represent mean to max; *p < 0.05. (I) Heatmap depicts log_2_-transformed averaged expression of *CCL19* in indicated immune and non-immune populations in the TME of multiple human cancer types (HNSCC,^62^ *n* = 40,^63^ *n* = 18^64^ patients; CRC, *n* = 23,^65^ *n* = 64^66^ patients; ESCC, *n* = 58 patients^67^; NSCLC, *n* = 32,^68^ *n* = 7^26^ patients; BRCA, *n* = 29 patients^46^; and PRCA, *n* = 18 patients^69^). A cross indicates that the cellular population was not detected. See also Figures S1–S4.

In mouse tumors, CCR7^+^ DCs can accumulate near BVs instead of migrating via LVs to lymph nodes.^9,36^ To test whether this occurs in humans, we analyzed 11 head and neck squamous cell carcinomas (HNSCCs) (Table S1; Figure S1A) using anti-LAMP3 and CD11c monoclonal antibodies (mAbs) to identify CCR7^+^ DCs (Figures S1B–S1D). These cells were consistently found—often in clusters—near BVs or LVs, with BV-associated CCR7^+^ DCs predominating in most tumors (Figure 1A). We extended these findings to five endometrial cancer (EC) samples and four non-small cell lung cancer (NSCLC) samples (for the latter, CCR7^+^ DCs were defined by their mRNA signature^47^) (Figures 1A and S1A; Table S1). Perivascular CCR7^+^ DCs preferentially localized near venous rather than arterial vessels (Figures S1E–S1G), albeit with some variability. Overall, a large fraction of tumor CCR7^+^ DCs reside near BVs compared with LVs (HNSCC, 51% ± 16% vs. 20% ± 11%; EC, 40% ± 16% vs. 17% ± 16%; NSCLC, 83% ± 5% vs. 5% ± 2%; mean ± SEM for BV-associated vs. LV-associated CCR7^+^ DCs).

We next assessed their distribution in mouse tumor models (MC38 colon adenocarcinoma and B16F10 and D4M3.A.OVA melanomas), identifying CCR7^+^ DCs by FSCN1 staining^36^ (Figure S2A). As in human tumors, they localized near BVs and LVs, with BV-associated CCR7^+^ DCs predominating in most cases (MC38, 54% ± 14% vs. 8% ± 7%; B16F10, 36% ± 13% vs. 33% ± 15%; D4M3.A-OVA, 38% ± 8% vs. 13% ± 17%; mean ± SEM for BV-associated vs. LV-associated CCR7^+^ DCs) (Figure 1B) and clustering preferentially near venous BVs (Figures S2B and S2C). In both human and mouse tumors, LV-associated CCR7^+^ DCs often invaded the lumen, a necessary step for tumor-draining lymph node migration, whereas BV-associated CCR7^+^ DCs did not (Figures 1A, 1B, and S2A). These results reveal a conserved spatial organization of CCR7^+^ DCs in perivascular and lymphatic niches in both human and mouse tumors.

Since the above observations were made in untreated tumors, we next asked whether effective immunotherapies alter CCR7^+^ DC abundance and localization. MC38 tumor-bearing mice received anti-PD-1 or agonistic anti-CD40 mAbs. Both treatments induced tumor control (Figure S2D) and either maintained (with anti-PD-1) or increased (with anti-CD40) total tumor CCR7^+^ DC numbers 3 days later (Figures S2E and S2F). CCR7^+^ DCs formed larger clusters (Figure S2F) and remained more often located near BVs than LVs (anti-PD-1, 61% ± 15% vs. 11% ± 8%; anti-CD40, 64% ± 8% vs. 4% ± 2%; mean ± SEM for BV-associated vs. LV-associated CCR7^+^ DCs) (Figure 1C). Consistently, intravital imaging of *Il12*-*p40*-eYFP^+^ cells in MC38 tumors—which mark a subset of CCR7^+^ DCs (Figures S2G and S2H) known to sustain T cell effector functions during immunotherapy^9,36^—showed persistent BV localization, both within minutes and up to several days after anti-PD-1 administration (Figures S2I and S2J), and reduced motility 1 day after anti-PD-1 (Figure S2J). Thus, tumor CCR7^+^ DCs remain perivascular during effective antitumor immune responses, consistent with their proposed role in local immune activation.^9,36,70^

### The CCL19-CCR7 axis maintains tumor CCR7^+^ DCs near BVs

The conserved perivascular organization of CCR7^+^ DCs in human and mouse tumors highlights the relevance of mouse models for investigating the mechanisms guiding perivascular positioning. CCR7 facilitates DC migration to lymph nodes via LVs^32,33,35^ and was expressed at similar levels by BV- and LV-associated CCR7^+^ DCs (Figure S3A). However, whether CCR7 also regulates the positioning of CCR7^+^ DCs near BVs required study. We examined these cells, defined as FSCN1^+^, in MC38 tumors from *Ccr7*^*wt/wt*^, *Ccr7*^*ko/wt*^, and *Ccr7*^*ko/ko*^ mice treated with anti-PD-1 (Figure S3B). In *Ccr7*^*ko/ko*^ mice, tumors grew faster and tumor-draining lymph nodes were smaller (Figures S3C–S3E), indicating weaker antitumor immunity, despite similar total FSCN1^+^ DC numbers (Figure S3F) and vessel densities (Figure S3G). However, perivascular FSCN1^+^ DC clusters were smaller (Figures 1D and S3H) and more loosely distributed in *Ccr7*^*ko/ko*^ mice than in controls (Figure 1D). As expected, clustering near LVs was also impaired (Figures S3I and S3J). These results indicate that CCR7 controls not only DC migration to LVs but also their positioning near BVs.

We analyzed the spatial distribution of CCR7 ligands CCL19 and CCL21 to assess whether they might contribute to the positioning of perivascular CCR7^+^ DCs. Using *Ccl19*-ieYFP mice,^71^ we found *Ccl19*^+^ cells forming perivascular layers around subsets of tumor BVs (Figures 1E, S3K, and S3L) and, less frequently, around LVs (Figure S3M). In contrast, CCL21 was undetectable around BVs but enriched within and around LVs (Figures 1E and S3M), consistent with the extra- and intraluminal positioning of CCR7^+^ DCs at LVs (Figures 1A, 1B, and S2A). Accordingly, LV-associated CCR7^+^ DC clusters localized in regions containing both CCL21 and *Ccl19* (Figure S3M), whereas BV-associated CCR7^+^ DC clusters localized to *Ccl19*-rich, CCL21-poor areas (Figures 1E and S3N). To test whether CCL19 drives perivascular clustering, we analyzed *Ccl19*^*ko/ko*^ and *Ccl19*^*wt/wt*^ mice bearing MC38 tumors and treated with anti-PD-1. *Ccl19* deficiency did not affect overall CCR7^+^ DC, BV, or LV abundance within tumors (Figures S3O and S3P) and preserved LV clustering (Figure S3Q) but profoundly impaired BV-associated CCR7^+^ DC clustering (Figure 1F). Thus, CCR7 and its ligand CCL19 coordinate perivascular CCR7^+^ DC organization in tumors.

scRNA-seq datasets from mammary,^57,58^ lung^59^ (and GEO: GSE201247), and pancreatic^60,61^ mouse tumor models show *Ccl19* expression restricted to fibroblasts (Figure 1G). Histological analysis confirmed that BV-associated *Ccl19*^+^ cells in MC38 tumors expressed the fibroblast markers alpha smooth muscle actin (αSMA) and desmin (DES) (Figure S3R), consistent with prior observations of CCL19^+^ fibroblasts within tumors.^47,72–76^ Spatial transcriptomics of human NSCLC^47^ identified *CCL19*^+^ fibroblasts near BV-associated CCR7^+^ DCs (Figure 1H), with *CCL19*^+^ fibroblasts enriched within a five-cell radius of BV-associated CCR7^+^ DCs, confirming non-random co-localization (Figure 1H). Analysis of human scRNA-seq datasets from HNSCC (*n* = 52,^62^ *n* = 40,^63^ and *n* = 18^64^ patients), colorectal cancer (CRC *n* = 23^65^ and *n* = 64^66^ patients), esophageal cancer (ESCC *n* = 58 patients^67^), NSCLC (*n* = 32^68^ and *n* = 7^26^ patients), breast cancer (BRCA *n* = 29 patients^46^), and prostate cancer (PRCA *n* = 18 patients^69^) show *CCL19* enrichment in fibroblasts. Notably, unlike in mouse tumors, human CCR7^+^ DCs also express *CCL19* (Figure 1I), suggesting that CCL19 from human CCR7^+^ DCs could enhance their own clustering through a positive feedback loop. Together, these findings uncover a conserved CCR7-CCL19 axis organizing perivascular CCR7^+^ DCs in both mouse and human tumors.

To test whether anti-PD-1 amplifies the CCL19 axis, as suggested by larger CCR7^+^ DC clusters after treatment, we analyzed a scRNA-seq dataset of paired biopsies from 40 breast cancer patients receiving anti-PD-1.^46^ Patients were classified as showing T cell clonotype expansion during treatment (“E” patients) or not (“NE” patients). On-treatment samples from E patients contained more *CCL19*^+^ fibroblasts than those from NE patients (Figure S4A), and their fibroblasts already expressed higher *CCL19* levels before therapy (Figure S4B), suggesting preferential activity of *CCL19*^+^ fibroblasts in responders. However, within individual patients, we saw no increase in the frequency of *CCL19*^+^ fibroblasts or their *CCL19* expression (Figures S4A and S4B), indicating that enhanced perivascular CCR7^+^ DC clustering after therapy is unlikely to result from increased fibroblast *CCL19* activity.

### Tregs are prime interaction partners of perivascular CCR7^+^ DCs

**Figure 2.**
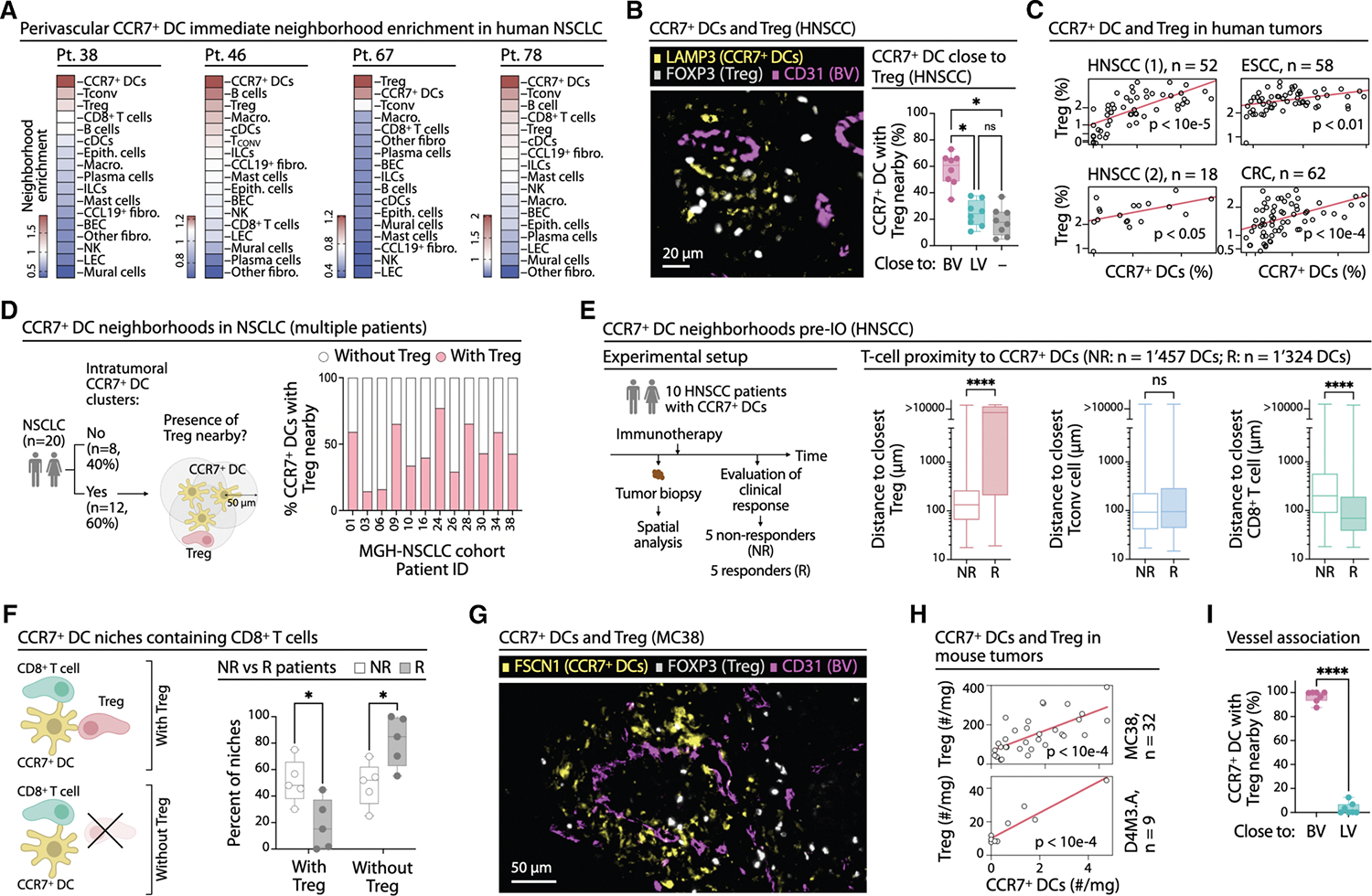
Tregs populate perivascular CCR7^+^ DC niches in human and mouse tumors (A) Heatmaps depict the enrichment of immune and non-immune cell types in the immediate neighborhood of CCR7^+^ DCs in NSCLC spatial transcriptomic data (*n* = 4).^47^ (B) (Left) Representative FOV displaying CCR7^+^ DCs (HLA-DR^+^LAMP3^+^; yellow) located near BVs (CD31^+^PDPN^−^; magenta) and Tregs (CD4^+^FOXP3^+^; white) in one HNSCC sample using high-plex whole-tissue imaging. Scale bar represents 20 μm. (Right) Box plots display the frequencies of BV-associated, LV-associated, and non-vessel-associated CCR7^+^ DCs close (<5 μm) to Tregs among all tumor CCR7^+^ DCs with nearby Tregs. Wilcoxon test, whiskers represent min to max; *p < 0.05. (C) Correlations between CCR7^+^ DCs and Tregs within CD45^+^ cells, as determined by scRNA-seq in multiple human cancer types. Spearman rank correlation; significant correlations are shown with a fitted red line. (D) (Left) Scheme outlining the analyses of CCR7^+^ DCs and Tregs in NSCLC samples. Patients with numerous (>5) CCR7^+^ DC clusters (*n* = 12) were selected for downstream analyses. (Right) Frequency of CCR7^+^ DCs (CD11c^+^LAMP3^+^) with at least one nearby (<50 μm) Treg (CD4^+^FOXP3^+^) in each individual patient. Numbers of FOVs analyzed per sample are as follows: NR01, *n* = 126; NR06, *n* = 455; NR09, *n* = 180; NR12, *n* = 79; NR26, *n* = 293; R11, *n* = 122; R15, *n* = 205; R35, *n* = 175; R37, *n* = 459; R45, *n* = 276. (E) (Left) Scheme outlining the analysis of tumor biopsies from HNSCC patients before immunotherapy (pre-IO). Patients were divided into non-responders (NR, *n* = 5) and responders (R, *n* = 5) based on the assessment of clinical response at 6 months. (Right) CCR7^+^ DC shortest distance to Tregs, T_CONV_, and CD8^+^ T cells in NR versus R tumors. Data are shown for all CCR7^+^ DCs compiled (NR tumors, *n* = 1,457 cells; R tumors, *n* = 1,324 cells). Unpaired *t* test, whiskers represent min to max; ****p < 0.0001. Numbers of FOVs analyzed per sample as in (D). (F) (Left) Scheme outlining the analyses of CCR7^+^ DC-CD8^+^ T cell niches. (Right) Frequencies of CCR7^+^ DC-CD8^+^ T cell niches with or without Tregs in their proximity (<100 μm). Two-way ANOVA with multiple comparisons, whiskers represent min to max; *p < 0.05. Numbers of FOVs analyzed per sample as in (D). (G) Representative FOV displaying CCR7^+^ DCs (FSCN1^+^ cells; FSCN1 in yellow) located near BVs (CD31^+^LYVE-1^−^ cells; CD31 in magenta) and Tregs (FOXP3^+^ cells; FOXP3 in white) in untreated MC38 tumors. Scale bar represents 50 μm. (H) Correlations between the numbers of CCR7^+^ DCs and Tregs per mg of tumor tissue, as determined by fluorescence-activated cell sorting (FACS) analyses of MC38 and D4M3. A tumors. Spearman rank correlation; significant correlations are shown with a fitted red line. (I) Box plots show the frequencies of tumor CCR7^+^ DCs close (<5 μm) to Tregs that are associated to BVs or LVs in MC38 tumors (*n* = 7). Whole-tumor sections were analyzed. Paired *t* test, whiskers represent min to max; ****p < 0.0001. See also Figures S5 and S6.

Given the importance of perivascular CCR7^+^ DCs in antitumor immunity, we hypothesized that their neighbors comprise not only effectors populations (e.g., CD8^+^ T cells^9,23,36,47^), but also regulators influencing CCR7^+^ DC functions. To identify such partners, we analyzed spatial transcriptomic data from four NSCLC patients,^47^ reclassifying cell types and calculating neighborhood enrichment scores (Figures 2A and S5A). As expected, CCR7^+^ DCs were highly enriched, reflecting their clustering behavior, along with CD4^+^ conventional (T_CONV_) and Treg cells (Figures 2A and S5A), while CD8^+^ T cells and B cells showed variable enrichment.

Treg proximity was confirmed in eight HNSCC samples (Figure 2B), as well as in NSCLC, a uterine cancer (UC), and CRC tumors^77^ (Figures S5B–S5D). These interactions occurred preferentially—albeit not exclusively—near BVs (Figure 2B). Comparison across six tumor samples (five NSCLC, one CRC)^47,77^ showed that Tregs were more enriched around CCR7^+^ DCs than around other DCs in five of six tumors (Figures S5E and S5F). Analysis of scRNA-seq datasets from 190 tumors (HNSCC^62,64^, ESCC^67^, and CRC^66^) revealed that CCR7^+^ DCs comprised on average one-quarter of all intratumoral DCs (Figures S6A–S6C), and their abundance correlated positively with that of Tregs (Figure 2C), but typically not with other T cell subsets (Figure S6D). These findings suggest a conserved, coordinated association between CCR7^+^ DCs and Tregs within perivascular niches across tumor types.

We next examined CCR7^+^ DC-Treg spatial associations in two patient cohorts. In 20 NSCLC specimens, 12 contained numerous CCR7^+^ DC clusters, and Tregs were consistently detected within 50 μm of these clusters (Figure S6E), although the frequency of nearby Tregs varied (Figure 2D), indicating interpatient heterogeneity that may drive localized immune regulation. In 10 HNSCC biopsies obtained before anti-PD-1-containing immunotherapy (5 responders [R] and 5 non-responders [NR]), Tregs were on average significantly closer to CCR7^+^ DCs in NR compared to R patients (Figure 2E). By contrast, CCR7^+^ DCs were on average equidistant to T_CONV_ and farther from CD8^+^ T cells in NR compared with R patients (Figure 2E). Since intratumoral DC-CD8^+^ T cell interactions correlate with better tumor control and immunotherapy response,^15,17,23,47,78–80^ we specifically examined CCR7^+^ DC niches containing CD8^+^ T cells. NR tumors showed more CCR7^+^ DC-CD8^+^ T cell interactions with Tregs nearby, whereas R tumors showed more interactions without Tregs (Figure 2F). These data suggest that close Treg proximity to tumor CCR7^+^ DCs impairs antitumor immunity.

To further study these interactions, we examined MC38 and D4M3.A mouse tumors. Tregs were frequently located near CCR7^+^ DCs (Figures 2G and S6F), and their abundance broadly correlated with that of CCR7^+^ DCs (Figures 2H and S6G). Most CCR7^+^ DC-Treg interactions occurred at BV sites (Figures 2I and S6H), where *Ccl19*^+^ cells were also found (Figure S6I). These findings suggest that the perivascular niche is a privileged site for CCR7^+^ DC interactions with Tregs in both human and mouse tumors, and that mouse models are relevant to study these interactions.

### Treg depletion improves the antitumor functions of tumor CCR7^+^ DCs

**Figure 3.**
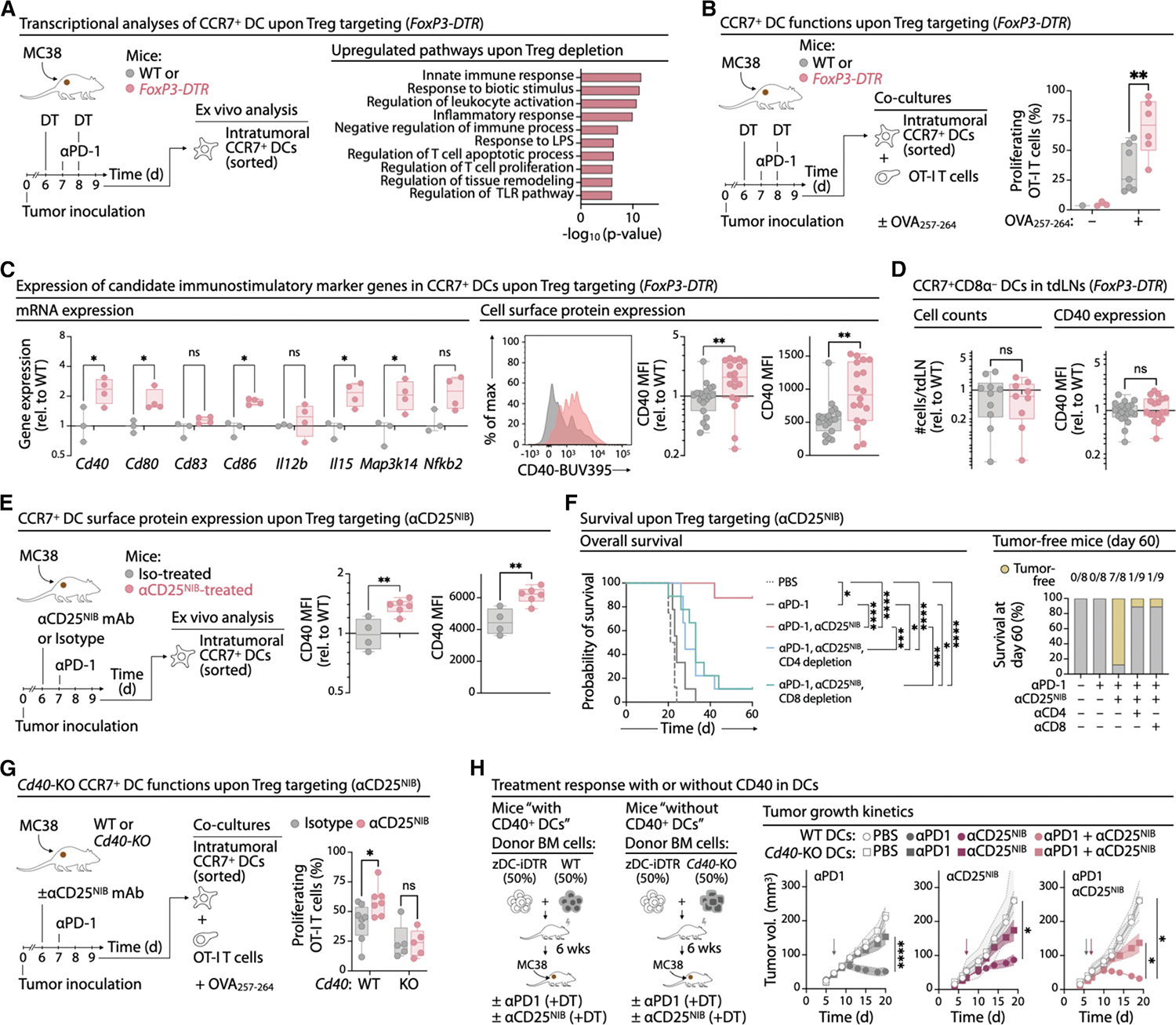
Tregs reversibly suppress antitumor functions of CCR7^+^ DCs at perivascular niches (A) (Left) Scheme outlining the experimental setup for bulk RNA-seq analyses of tumor-derived CCR7^+^ DCs. (Right) GO pathway enrichment analyses performed on differentially expressed genes (DEGs) in CCR7^+^ DCs in MC38 tumors (*n* = 4) from Treg-depleted (*FoxP3*-DTR) compared with Treg-sufficient (WT) mice. Bar plot indicates the −log_10_ raw binomial *p*-values of the top 10 most enriched pathways in CCR7^+^ DCs. (B) (Left) Experimental setup for *ex vivo* stimulation of OT-I CD8^+^ T cells with tumor CCR7^+^ DCs. (Right) Percentage of OT-I CD8^+^ T cells that proliferated after 5-day culture with OVA_257–264_ peptides-loaded CCR7^+^ DCs isolated from WT or Treg-depleted tumors. As a control, CCR7^+^ DCs without OVA_257–264_ peptides were used. Two-way ANOVA with multiple comparisons, whiskers represent min to max; **p < 0.01. (C) (Left) Relative gene expression levels analyzed by bulk RNA-seq. Each dot represents one mouse (*n* = 4), whiskers represent mean to max. Unpaired *t* test with multiple comparisons; *p < 0.05. (Right) Representative histogram of CD40 protein expression and relative mean fluorescence intensity (MFI) measured by FACS and expressed both as normalized values and absolute MFI. Each dot represents one mouse (*n* = 18), whiskers represent min to max. Unpaired *t* test; **p < 0.01. (D) Analyses of cDCs in tumor-draining lymph nodes. Absolute cell counts (left, *n* = 10) and MFI of CD40 expression (right, *n* = 18) measured by FACS in migratory cDCs (CCR7^+^CD8α^−^) from WT or Treg-depleted mice. Whiskers represent mean to max. (E) (Left) Experimental setup for *ex vivo* analyses of tumor CCR7^+^ DCs isolated from anti-PD-1-treated mice that received or not αCD25^NIB^ mAbs. (Right) CD40 protein expression measured by FACS and expressed both as normalized values and absolute MFI. Each dot represents one mouse (*n* = 4 WT and *n* = 6 FoxP3-DTR), whiskers represent min to max. Unpaired *t* test; **p < 0.01. (F) (Left) Overall survival analyses of MC38 tumor-bearing mice treated, or not treated, with αPD-1 and αCD25^NIB^ mAbs, and in which CD4^+^ or CD8^+^ cells were depleted or not (*n* = 8 or 9 mice/group). Log-rank Mantel-Cox test; *p < 0.05, ***p < 0.001, and ***p < 0.0001. (Right) Percentage of tumor-free mice on day 60 in the indicated treatment groups. (G) (Left) Experimental setup for *ex vivo* stimulation of OT-I CD8^+^ T cells with tumor CCR7^+^ DCs as in (B). The DCs were obtained from mice receiving anti-PD-1 immunotherapy and that were treated or not with αCD25^NIB^ mAbs. (Right) Percentage of OT-I CD8^+^ T cells that proliferated after 5-day culture with OVA_257–264_ peptide-loaded CCR7^+^ DCs. Each dot represents one mouse (*n* = 8 and *n* = 7), whiskers represent min to max. Two-way ANOVA with multiple comparisons; *p < 0.05. (H) (Left) Scheme outlining bone marrow chimeras with inducible *Cd40*-deficiency in cDCs and the treatment schedule. (Right) Growth curves of MC38 tumors inoculated in zDC^iDTR^:*Cd40*^WT^ and zDC^iDTR^:*Cd40*^KO^ bone marrow chimeras treated with αPD-1, αCD25^NIB^, or αPD-1 + αCD25NIB combination (*n* = 8–10 mice/group). Mean with SEM. Two-way ANOVA with multiple comparisons; *p < 0.05 and ****p < 0.0001. See also Figures S7 and S8.

To investigate how Tregs affect CCR7^+^ DC functions during immunotherapy, we examined CCR7^+^ DCs from MC38 tumor-bearing *FoxP3*-DTR mice—where CCR7^+^ DCs are mainly perivascular—after diphtheria toxin-induced Treg depletion and anti-PD-1 treatment (Figure S7A). Transcriptome and pathway analyses of CCR7^+^ DCs isolated from these Treg-depleted mice (Figure S7B) revealed enrichment of genes associated with regulation of T cell activation and proliferation, compared with CCR7^+^ DCs from control Treg-sufficient mice (Figure 3A). Functionally, CCR7^+^ DCs from Treg-depleted mice induced stronger OVA-specific OT-I CD8^+^ T cell proliferation *ex vivo* (Figure 3B), suggesting that Tregs limit the local stimulatory activity of tumor CCR7^+^ DCs.

Treg depletion also increased expression of immunostimulatory genes (*Cd40*, *Cd80*, *Cd86*, *Il15*, and *Map3k13*^9,36,81–85^; Figure 3C), and surface protein expression of CD40 (Figure 3C), CD80, and CD86 (Figures S7C and S7D). CCR7 levels remain unchanged (Figure S7E), suggesting that CCR7 expression is independent of other DC activation markers. CD40 upregulation occurred selectively in CCR7^+^ DCs, whereas CD80 and CD86 increased across all tumor cDCs (Figures S7C and S7D). In tumor-draining lymph nodes, Treg depletion did not increase CCR7^+^ DC numbers or their CD40 expression (Figure 3D), though CD80 and CD86 were upregulated in both CCR7^−^ and CCR7^+^ DCs (Figures S7F and S7G). These results indicate that Tregs suppress immunostimulatory programs of tumor CCR7^+^ DCs, most notably CD40 expression.

To deplete Tregs in a therapeutically relevant setting, we combined anti-PD-1 immunotherapy with a non-IL-2-blocking CD25-depleting antibody (anti-CD25^NIB^), which targets Tregs while preserving IL-2 signaling in effector T cells^86^ (Figure 3E). In MC38 tumors, T_CONV_ and CD8^+^ T cells showed minimal CD25 expression compared to Tregs (Figure S8A). Analysis of TMEs and draining lymph nodes confirm efficient and specific Tregs depletion by anti-CD25^NIB^ (Figures S8B and S8C). This treatment increased CD40 protein (Figure 3E) and *Cd40* mRNA (Figure S8D) in tumor CCR7^+^ DCs (Figures S8E and S8F), replicating the effects of genetic Treg depletion.

Together, these findings indicate that Tregs limit the expression of T cell stimulatory molecules in tumor CCR7^+^ DCs, an effect reversible by Treg ablation. The selective induction of CD40 in tumor CCR7^+^ DCs after Treg depletion suggests that CD40 on these cells is critical for tumor control when Tregs are absent.

### Treg-mediated control of CD40 expression by CCR7^+^ DCs limits antitumor immunity

The results above raise the question of how Tregs limit CCR7^+^ DC function. Since CD40 expression by DCs facilitates interactions with CD40L^+^ T_CONV_ and promotes CD8^+^ T cell responses and tumor control,^9,39,87–91^ Treg-driven inhibition of CD40 on CCR7^+^ DCs may have implications for antitumor immunity. To test this, we compared immune responses in MC38 tumor-bearing mice treated with anti-PD-1 alone or combined with anti-CD25^NIB^. While anti-PD-1 alone triggered initial tumor regression but no lasting control, the combination achieved durable responses, with 90% of mice tumor-free at day 60 (Figure 3F). This effect required both T_CONV_ and CD8^+^ T cells (Figures 3F, S8G, and S8H). These findings indicate that Tregs hinder effective immunotherapy responses by impairing CD8^+^ and CD4^+^ T cell antitumor activity in this model. In addition, the therapeutic benefit conferred by Treg depletion provided us with an experimental setup to investigate whether Treg-driven control of CD40 expression by CCR7^+^ DCs contributes to the observed effects.

To investigate whether Tregs suppress CCR7^+^ DC function through CD40 regulation, we isolated tumor CCR7^+^ DCs from wild-type (WT) and *Cd40*-deficient mice, with or without prior treatment with anti-CD25^NIB^ mAbs. These DCs were pulsed *ex vivo* with the OVA peptide SIINFEKL and co-cultured with OVA-specific OT-I CD8^+^ T cells. CCR7^+^ DCs from WT mice treated with anti-CD25^NIB^ exhibited a significantly enhanced ability to stimulate CD8^+^ T cell proliferation (Figure 3G), consistent with results from genetic Treg depletion (Figure 3B). In contrast, *Cd40*-deficient CCR7^+^ DCs failed to increase CD8^+^ T cell proliferation, regardless of anti-CD25^NIB^ treatment (Figure 3G). Combined with prior evidence that CD40 agonism boosts CCR7^+^ DC-driven CD8^+^ T cell activation,^9^ these results suggest that Tregs limit antitumor immunity at least in part by suppressing CD40 expression on tumor CCR7^+^ DCs.

To determine whether CD40 expression by cDCs is necessary for tumor control, we generated mixed bone marrow chimeras in which *Zbtb46*-dependent cDCs lacked CD40, while other cell types, including macrophages, retained it (Figures 3H, S8I, and S8J). This model allowed conditional deletion of CD40 specifically in cDCs after tumor establishment. During the initial 5 days of tumor growth, T cell and Treg responses developed in the presence of CD40-sufficient DCs. On day 5, immediately before anti-CD25^NIB^ and anti-PD-1 immunotherapy, diphtheria toxin was given to deplete *Zbtb46*-DTR-derived cDCs. As a result, WT:*Zbtb46*-DTR chimeras yielded mice with WT DCs, whereas *Cd40*-deficient:*Zbtb46*-DTR chimeras yielded mice with *Cd40*-deficient DCs (Figure S8K). Using this setup, we found that CD40-expressing cDCs were required for tumor control under anti-PD-1 and Treg-targeting treatments, both individually and in combination (Figures 3H and S8L). These findings indicate that Tregs can suppress key immunostimulatory pathways in CCR7^+^ DCs, notably CD40 signaling, which is critical for effective antitumor immunity.

### Immunotherapy reinforces Treg interactions with perivascular tumor CCR7^+^ DCs

Anti-PD-1 therapy enhances both the clustering of CCR7^+^ DCs around BVs (Figures S2E and S2F) and their antitumor functions.^25^ In particular, therapy-induced IL-12 production by CCR7^+^ DCs is critical for local CD8^+^ T cell activation.^9,21,38–41^ However, tumor CCR7^+^ DCs can also interact with Tregs (Figure 2), limiting their stimulatory capacity (Figure 3). We therefore investigated whether anti-PD-1 therapy reduces suppressive CCR7^+^ DC:Treg interactions around BVs.

**Figure 4.**
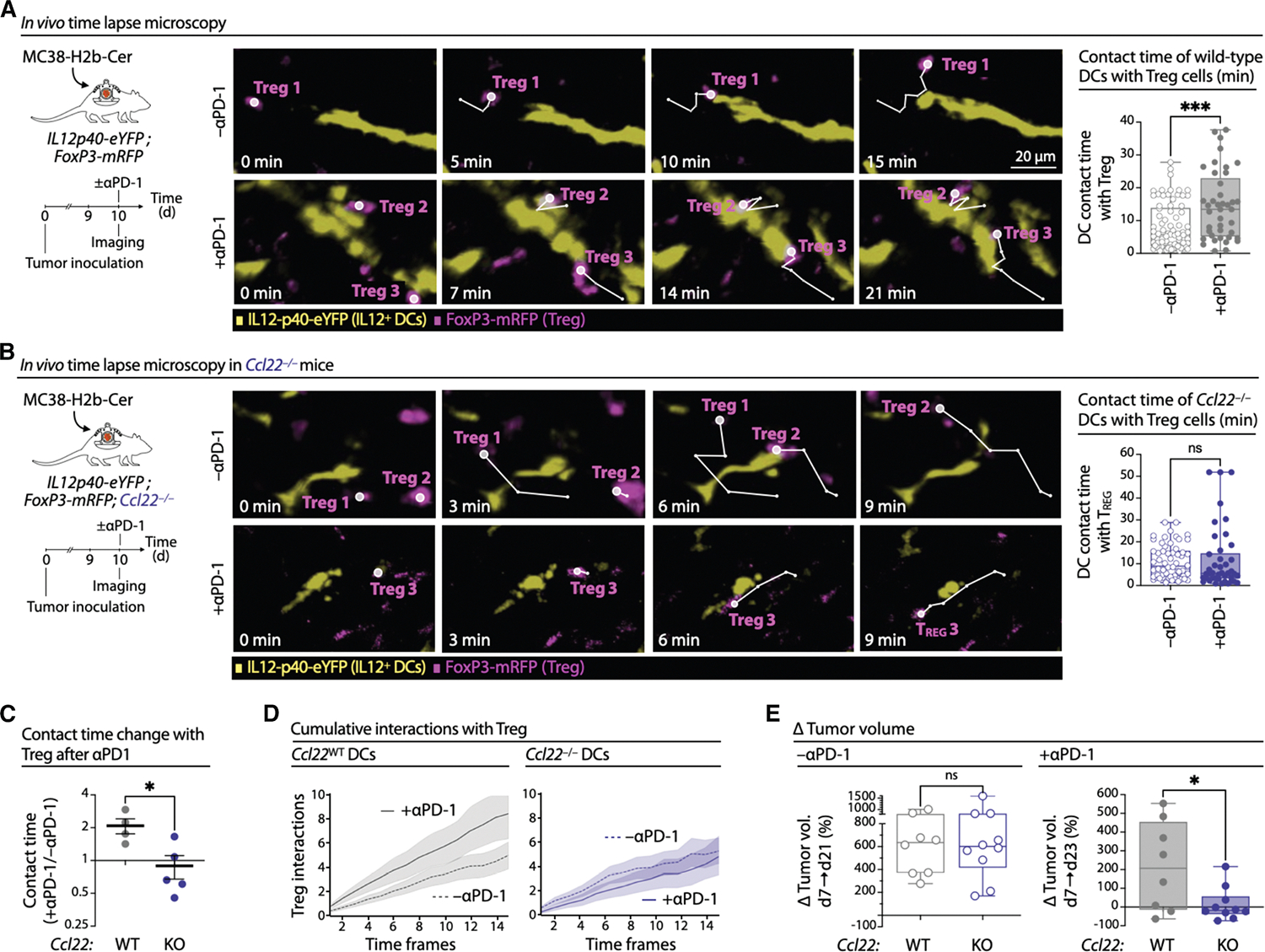
Immunotherapy enhances perivascular tumor CCR7^+^ DC interactions with neighboring Tregs via Ccl22 (A) (Left) Outline of *in vivo* two-photon time-lapse microscopy in MC38-H2b-Cerulean tumors. (Middle) Representative FOVs displaying the migratory behavior of Tregs (*FoxP3*-mRFP, magenta) in untreated (top) or anti-PD-1-treated (bottom) mice in perivascular regions containing *Il12*-eYFP^+^ DCs (yellow). Scale bar represents 20 μm. Treg migratory tracks are shown in white. (Right) Observed contact duration between DCs and Tregs in each experimental group (*n* = 4). Each dot represents one DC, whiskers represent mean to max. Unpaired *t* test; ***p < 0.001. (B) As in (A), but in *Il12-p40*-eYFP; *FoxP3*-mRFP; *Ccl22*^*−/−*^ mice (n = 5). (C) Change in contact duration between DCs and Tregs after anti-PD-1 treatment, comparing *Ccl22*^WT^ and Ccl22^−/−^ mice. Each dot represents one mouse, bar represents mean with SEM. Unpaired *t* test; *p < 0.05. (D) Quantification of the cumulative interactions of CCR7^+^ DCs with Tregs over time of acquisition, comparing *Ccl22*^WT^ and Ccl22^−/−^ mice. Shaded areas indicate confidence intervals. (E) Change in tumor volume in WT and *Ccl22*-deficient mice left untreated (left), or upon anti-PD-1 therapy (right). Unpaired *t* test, whiskers represent min to max; *p < 0.05. See also Figure S9.

Using *in vivo* time-lapse two-photon microscopy, we tracked tumor CCR7^+^ DC:Treg dynamics at perivascular sites in MC38 tumor-bearing *Il12-p40*-eYFP × *FoxP3*-mRFP reporter mice (Figure S9A). We measured contact durations between Tregs (*FoxP3*-mRFP^+^) and perivascular CCR7^+^ DCs (*Il12*-*p40*-eYFP^+^ located near BVs^36^), with BVs visualized with quantum dots (Figure 4A). In untreated mice, median contact time was 5.6 min (2.1–13.9 min, 25%–75% percentile). Surprisingly, this increased significantly to 13.4 min (5.2–22.9 min) 1 day after anti-PD-1 therapy (Figure 4A). Thus, while anti-PD-1 enhances the antitumor functions of perivascular CCR7^+^ DCs,^9^ it also prolongs their interactions with Tregs, potentially counteracting part of the therapy’s tumor eradication effects.

### CCL22 enhances CCR7^+^ DC:Treg interactions during immunotherapy

The mechanisms underlying the extended interaction times between perivascular tumor CCR7^+^ DCs and Tregs during immunotherapy remain unclear. Since CCL22 can attract tumor-infiltrating Tregs^56,92,93^ (Figure S9B) and was expressed by CCR7^+^ DCs in both mouse and human tumors (Figure S9C), we investigated its role in these cell-cell interactions. We generated *Il12-p40*-eYFP × *FoxP3*-mRFP × Ccl22^−/−^ mice, in which *Il12*-*p40*-eYFP^+^ DCs lack CCL22 expression and performed intravital microscopy as above.

In untreated Ccl22^−/−^ mice, the median contact time between *FoxP3*-mRFP^+^ Tregs and perivascular CCR7^+^ DCs was 8.9 min (4.3–15.9 min). Notably, anti-PD-1 therapy did not prolong these interactions, with the median contact time remaining at 5.6 min (3.3–14.8 min) (Figure 4B). These results contrast with those obtained in CCL22-sufficient mice. Indeed, the increase in contact time with *FoxP3*-mRFP^+^ Tregs induced by anti-PD-1 therapy was significantly higher for WT *Il12*-*p40*-eYFP^+^ cells compared to their Ccl22^−/−^ counterparts (Figure 4C). In addition, analysis of the cumulative number of interactions between individual CCR7^+^ DCs and Tregs over time revealed that anti-PD-1 treatment increased this number in WT mice but not in their *Ccl22*-deficient counterparts (Figure 4D). Finally, impaired Treg recruitment did not account for the reduced DC-Treg interaction times in *Ccl22*-deficient mice following immunotherapy (Figure S9D). These results indicate that CCL22 mediates the extended CCR7^+^ DC-Treg contacts during anti-PD-1 therapy.

Consistently, tumor growth was comparable between WT and *Ccl22*-deficient mice under untreated conditions; however, anti-PD-1 therapy more effectively controlled tumor progression in *Ccl22*-deficient mice (Figures 4E and S9E). Other chemokine-receptor axes involved in DC-T cell interactions were not substantially modulated by anti-PD-1 immunotherapy (Figure S9F). These findings suggest that CCL22 facilitates the prolonged suppressive interactions between CCR7^+^ DCs and Tregs during immunotherapy, thereby limiting treatment efficacy. Conversely, interfering with this mechanism can improve immunotherapy responses.

### Co-expression of stimulatory and regulatory molecules by single CCR7^+^ DCs

Although CCR7^+^ DCs promote effector T cell responses through IL-12 production and CD40 expression, their CCL22 expression may increase susceptibility to Treg-mediated suppression. This raised the question of whether individual CCR7^+^ DCs simultaneously express stimulatory (e.g., IL-12 and CD40) and suppressive (e.g., CCL22) molecules or represent distinct subsets with divergent functions.

**Figure 5.**
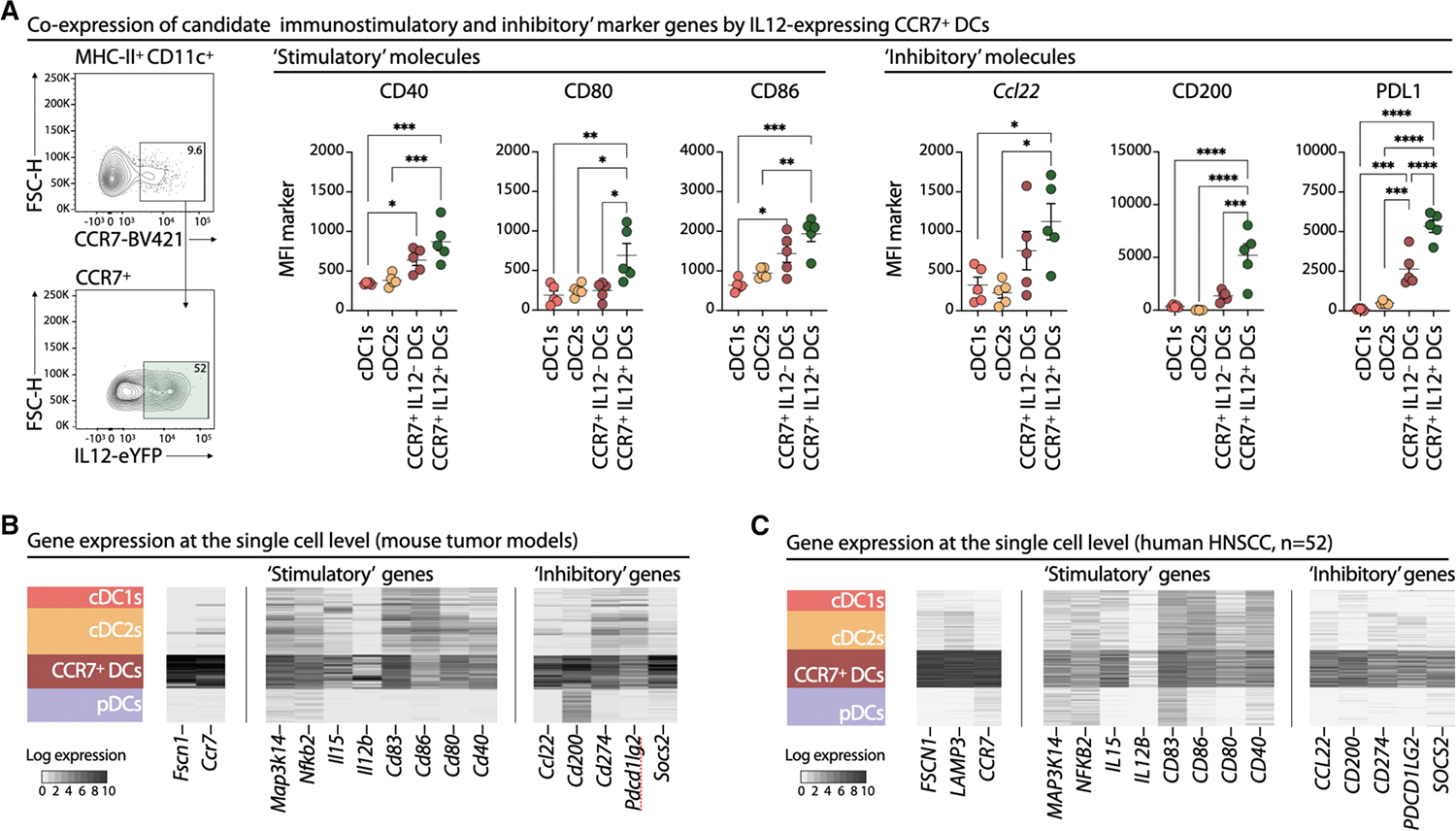
Individual CCR7^+^ DCs co-express stimulatory and regulatory molecules (A) (Left) FACS plots display the gating strategy for the analyses of IL-12-eYFP^+^CCR7^+^ DCs. (Right) Graphs show MFI values of indicated stimulatory and inhibitory markers in cDC1s, cDC2s, CCR7^+^ DCs, and IL12-eYFP^+^CCR7^+^ DCs. Expression of *Ccl22* transcripts was analyzed by PrimeFlow. Each dot represents one mouse (*n* = 5), bar represents mean with SEM. Ordinary one-way ANOVA test was performed; *p < 0.05, **p < 0.01, ***p < 0.001, and ****p < 0.0001. (B) Heatmap displays the expression of genes (with stimulatory or inhibitory function) at single-cell level in individual DCs in each state in mouse tumor models. (C) As in (B), but in HNSCC tumors. See also Figure S10.

To address this, we profiled tumor CCR7^+^ DCs at the single-cell level, focusing on those producing IL-12 and assessing CD40 and *Ccl22* (Figure 5A). IL-12-expressing CCR7^+^ DCs showed the highest CD40 levels, indicating coordinating expression of multiple stimulatory molecules driving effector T cell responses. However, these same also expressed the highest *Ccl22* levels, indicating that the very cells that drive effector T cell responses are simultaneously equipped with mechanisms rendering them vulnerable to Treg-mediated suppression. Extending this analysis, IL-12^+^ CCR7^+^ DCs also co-expressed additional stimulatory (CD80 and CD86) and suppressive (PD-L1 and CD200) molecules, confirming concomitant activation of both immune-promoting and regulatory programs within single cells (Figure 5A).

To validate these findings across tumor types, we analyzed tumor scRNA-seq data from 24 mice representing five orthotopic models: Kras/p53-driven lung adenocarcinoma,^26^ B16F10 melanoma, LLC lung carcinoma,^94^ MMTV-PyMT mammary cancer,^57,58^ IDH glioma,^95^ and Nras/Pten-driven liver cancer.^96^ Hierarchical clustering revealed shared DC states across models, including CCR7^+^ DCs (Figure S10A). At the single-cell level, CCR7^+^ DCs co-expressed stimulatory (e.g., *Il12b*, *Cd40*, *Cd80*, *Cd83*, *Cd86*, and *Il15*) and potentially suppressive (e.g., *Ccl22*, *Cd274*, *Cd200*, *Pdcd1lg2*, and *Socs2*) genes, all globally more highly expressed than in other DCs (Figure 5B).

Analysis of scRNA-seq data from 52 HNSCC tumors confirmed these findings: individual CCR7^+^ DCs co-expressed *IL12B*, *CD40*, *CD80*, *CD83*, *CD86*, and *IL15*, alongside *CCL22*, *CD274*, *CD200*, *PDCD1LG2*, and *SOCS2*, at higher levels than other DC populations (Figure 5C). To quantify this, we generated “stimulatory” and “inhibitory” scores based on curated gene sets and plotted them by DC subtype. CCR7^+^ DCs show the highest average stimulatory and inhibitory scores (Figures S10B and S10C).

Flow cytometry confirmed that CCR7^+^ DCs co-expressed CD40, IL-12, and CD86 together with PD-L1 and CD200, unlike cDC1s and cDC2s (Figure S10D). Cells with higher expression of stimulatory proteins also show higher levels of inhibitory markers. Taken together, these data indicate that CCR7^+^ DCs intrinsically co-activate both immunostimulatory and inhibitory programs. The concurrent expression of molecules such as CCL22 alongside IL-12 and other co-stimulatory markers suggests that these cells are wired for strong effector potential but are also inherently prone to Treg-mediated suppression, acting as a built-in brake on their activity.

## DISCUSSION

Tumor CCR7^+^ DCs are emerging as key players within the intratumoral circuits that facilitate antitumor immune responses. In this study, we uncovered two interconnected mechanisms that control the ability of tumor CCR7^+^ DCs to drive anticancer immune responses: (1) their CCL19:CCR7-dependent positioning in perivascular niches, and (2) their Treg-dependent regulation of stimulatory immune functions. The findings reveal additional rate-limiting steps in the cancer immunity cycle and highlight key regulators that may be exploited for therapeutic benefit, as discussed below.

Our study shows that tumor CCR7^+^ DCs preferentially localize near BVs in both human and mouse tumors, with their positioning governed by CCL19-CCR7 interactions. Fibroblasts lining venous BVs are a primary source of CCL19, facilitating local CCR7^+^ DC accumulation. These perivascular CCR7^+^ DC hubs serve as key sites for intratumoral T cell interactions and contribute to antitumor immunity.^9,36,70^ Mechanistically, this organization parallels that of lymph nodes, where CCL19- and CCL21-producing fibroblastic reticular cells guide DCs into T cell-rich paracortical zones.^97^ Within tumors, perivascular niches containing CCR7^+^ DCs resemble organized immune aggregates variably termed stem immunity hubs,^47^ T cell environments,^76^ or tertiary lymphoid structures (TLSs).^70,75,76,98^ Compositionally, all such intratumoral structures are enriched in CCR7^+^ DCs, CCL19^+^ fibroblasts, and T cells.^47,70,76^ Developmentally, DC-derived CCR7 and fibroblast-derived CCL19 are critical for TLS formation,^70,75^ and ablation of *Ccl19*^+^ fibroblastic precursors in lung tumors disrupts T cell environments.^76^ Functionally, these niches support local T cell-mediated antitumor immunity and are typically linked with improved patient outcomes.^47,70,75,76^ These data underscore the central role of the CCL19-CCR7 axis in orchestrating the spatial organization of CCR7^+^ DCs within tumors.

Building on our findings that CCR7^+^ DC clustering near BVs depends on CCL19-CCR7 interactions, and given the established role of CCL21-CCR7 signaling in guiding CCR7^+^ DC migration to lymph nodes,^32,33,35^ we propose a model in which the relative sensing of CCL19 versus CCL21 helps determine whether tumor CCR7^+^ DCs remain in perivascular niches or migrate to tumor-draining lymph nodes. The mechanisms regulating CCL19 expression and the balance between BV and LV localization during tumor progression remain unclear, and additional factors likely contribute. During immunotherapy, CCR7^+^ DCs form larger clusters without an increase in *CCL19*^+^ fibroblasts or *CCL19* transcripts, suggesting CCL19-independent mechanisms. For instance, anti-PD-1 therapy can trigger a feedback loop in which CD8^+^ T cell-derived IFN-γ enhances IL-12 production by CCR7^+^ DCs near BVs,^9^ illustrating how T cell-derived signals may shape DC positioning independently of CCL19.

Beyond their spatial organization, our study reveals a previously unappreciated regulatory mechanism that limits the function of tumor CCR7^+^ DCs. The presence of these cells in perivascular tumor niches is not sufficient for effective antitumor immunity, as they remain susceptible to Treg-mediated immunosuppression. This vulnerability is functionally significant: Tregs modulate the expression of key immunostimulatory genes in CCR7^+^ DCs, shifting their activity from immune activation toward tolerance. Mechanistically, Tregs suppress CD40 expression on tumor CCR7^+^ DCs, thereby impairing their ability to activate T cells, and weakening both CD4^+^ T_CONV_ and CD8^+^ T cell responses. Our findings align with previous data suggesting that increased CD40 signaling in CCR7^+^ DCs enhances CD8^+^ T cell responses and improves therapeutic outcomes.^9^ This regulatory checkpoint operates within perivascular niches, creating an immunosuppressive environment that becomes even more pronounced during anti-PD-1 immunotherapy. These findings extend earlier studies identifying Tregs as dominant suppressors of antitumor DC activity^5,49,50,53,56,99^ and reveal a localized mechanism by which Tregs constrain CCR7^+^ DC function in tumors. Our findings also highlight a functional duality in CCR7^+^ DCs. While these cells express potent immunostimulatory molecules such as IL-12 and CD40, they concurrently produce CCL22, which facilitates Treg interactions. This duality places tumor CCR7^+^ DCs at the interface of immune tolerance and tissue destruction, reinforcing the notion that the two processes are tightly linked.^100^ Such a regulatory balance may serve as a safeguard in tissues against excessive immune activation, but in tumors, it can instead suppress effective immune responses, even those induced by immunotherapy.

These findings have implications for immunotherapy. They suggest that therapeutic strategies should aim to preserve or enhance the CCL19 axis, which promotes the perivascular positioning and CCR7^+^ DCs within tumors, while limiting the CCL22 axis, which facilitates Treg-DC interactions that suppress DC activity. Interventions that strengthen CD40 expression on CCR7^+^ DCs could further counteract Treg-mediated inhibition and amplify their ability to drive effective T cell responses. Notably, the reversibility of Treg-mediated suppression highlights the potential for therapeutic reprogramming of tumor CCR7^+^ DCs. In conclusion, our study provides a framework for understanding how the CCR7-CCL19 axis organizes CCR7^+^ DCs into perivascular immune hubs that support antitumor immunity, and how Treg-mediated suppression, driven in part by CCL22, undermines their function. These insights identify spatial and molecular mechanisms that shape the tumor immune landscape and point to strategies for improving the efficacy of immunotherapy.

### Limitations of the study

We acknowledge limitations in the *ex vivo* proliferation assay used to assess CD8^+^ T cell responses following Treg perturbation in *Cd40*-deficient mice. This reductionist approach tested the intrinsic ability of tumor DCs to stimulate OT-I T cells and showed that DC-expressed CD40 is required for this function. However, because non-conditional *Cd40*-deficient mice were used, the absence of CD40 throughout DC development may have affected DC maturation or activation *in vivo*. Nevertheless, as DCs and T cells were obtained independently, the observed defect most likely reflects an intrinsic functional impairment of CD40-deficient DCs, rather than altered priming in lymph nodes. Consistent with this interpretation, *in vivo* bone marrow chimera experiments, in which CD40 was deleted in DCs only after T cell priming, confirmed that DC-expressed CD40 is required for tumor control after Treg depletion. Additionally, our data indicate a role of CCL22 signaling in Treg-CCR7^+^ DC interaction during immunotherapy, but we did not address the potential effects of CCL22 deficiency on other cellular interactions in the TME. Although Treg recruitment was unaltered in *Ccl22*-deficient mice, CCL22 loss may influence additional cell-cell interactions, either directly or indirectly, including Treg-CD8^+^ T cell associations, which could also contribute to the enhanced anti-PD-1 response observed in these mice.

## RESOURCE AVAILABILITY

### Lead contact

Further information or requests for resources or reagents should be directed to and will be fulfilled by the lead contact, Prof. Mikaël J. Pittet (mikael.pittet@unige.ch).

### Materials availability

This study did not generate new unique reagents.

### Data and code availability

- Single-cell RNA sequencing and MERFISH datasets are publicly available. Accession numbers are listed in the key resources table.
- Microscopy and flow cytometry data, multiplex immunofluorescence image QPTIFFs, single-cell AnnData objects, and code to reproduce the analyses and figures will be shared by the lead contact upon request.
- Any additional information required to reanalyze the data reported in this paper is available from the lead contact upon request.

## STAR★METHODS

### EXPERIMENTAL MODEL AND STUDY PARTICIPANT DETAILS

#### Animals

WT (C57BL6/J; Cat #000664), CD45.1 (JAX #002014) were purchased from Charles River Laboratories. *Cd40*-KO (JAX #002927), *Il-12p40*-IRES-eYFP (JAX #006412), *FoxP3*-IRES-mRFP (JAX #008374), *Ccr7*-GFP knockin/knockout (referred to as *Ccr7*^gfp/wt^ or *Ccr7*^ko/wt^; JAX #027913) and *FoxP3*-iDtr-GFP (JAX #016958) were purchased from Jackson Laboratories. *Ccl22*-KO mice were kindly provided by the laboratory of Prof. David Anz (Ludwig-Maximilians-Universität München, Germany). *Zbtb46*-iDtr mice (JAX #019506) were kindly provided by the laboratory of Prof. Ping-Chih Ho (University of Lausanne, Switzerland). *Ccl19*-KO (JAX #012851) and Ccl19-ieYFP (*Ccl19*-tTA × LC1-Cre × R26-eYFP) mice^72^ were kindly provided by the laboratory of Prof. Sanjiv Luther (University of Lausanne, Switzerland). OT-1 × CD45.1 were kindly provided by the laboratory of Prof. Daniel Speiser (University of Lausanne, Switzerland). *Il-12p40*-IRES-eYFP × *FoxP3*-IRES-mRFP and; *Il-12p40*-IRES-eYFP × *FoxP3*-IRES-mRFP × *Ccl22*-KO mice were generated at the Agora in Vivo Center (AIVC). All the mice, except C57BL6/J, CD45.1 and *Ccl19*-ieYFP mice, were bred in-house. Upon arrival, mice purchased from Charles River as well as *Ccl19*-ieYFP mice were housed in our facility for at least one week for acclimation before the start of the experiment.

#### Tumor cell lines

MC38 colorectal carcinoma cell lines, obtained from Dr. Mark Smyth (QIMR Berghofer), MC38-H2B-Cerulean, obtained from Dr. Mauro Di Pilato (MD Anderson Cancer Center) and D4M3.A-OVA melanoma cell lines, obtained from Dr. Thorsten R. Mempel (MGH Boston), were grown in DMEM-GlutaMAX (Gibco) supplemented with 10 % FBS and 1% antibiotics. B16F10 were purchased from ATCC and were grown in RPMI-GlutaMAX (Gibco) supplemented with 10 % FBS and 1% antibiotics. All cell lines were used in experiments when in exponential growth phase and tested for mycoplasma.

#### Human samples

Fresh tumor samples from HNSCC patients were collected with signed informed consent under IRB approved protocols Massachusetts General Hospital (MGH) and Massachusetts Eye and Ear (MEE) IRB#2014P000559; IRB#13–416; IRB#11–024H (Table S1). Archival FFPE tissues from NSCLC patients were collected with signed informed consent under IRB approved protocol Massachusetts General Hospital (MGH) DFCI #13–416 (Table S1). Archival FFPE tissues from endometrial patients were collected and the study was approved by the local ethical committee of Geneva under protocol CCER 2020–01385 (Table S1).

NSCLC samples were obtained at time of tumor resection. HNSCC biopsies or resections of tumor lesions were obtained prior to treatment with anti-PD-1 immunotherapy. HPV status of oropharyngeal tumors was determined based on p16 expression by immunohistochemistry (IHC) and/or by HPV 16/18 in situ hybridisation (ISH) and/or HPV PCR. Nasopharyngeal squamous cell carcinoma cases were tested for EBV by EBER ISH. Clinical demographic information, such as age, sex, smoking, and other, were extracted from electronic medical records. Non-smokers were defined as having a smoking history of <10 pack year. Response was determined based on clinical-radiological best response at 6 months. ‘Non-responders’ were patients with progressive disease (PD) or stable disease (SD) at 6 months after starting immunotherapy, whereas ‘responders’ are those with a partial (PR) or complete (CR) response.

### METHOD DETAILS

#### Mice studies

##### Tumor models

MC38 (2×10^6^), MC38-H2B-Cerulean (2×10^6^), D4M3.A-OVA (10×10^6^) and B16F10 (1×10^6^) cells were resuspended in sterile phosphate-buffered saline (PBS) or Hank’s balanced salt solution (HBSS) and inoculated subcutaneously in the right flank of each mouse in 50 μl volume. Pre-treatment tumor volumes were normalized between treatment groups. Tumor dimensions were measured using a digital caliper with the formula (d^2*D)/2 (where d and D represent the smallest and greatest dimensions, respectively). 6/7 days post tumor inoculation, MC38 tumor-bearing mice were treated with anti-PD-1, clone 29F.1A12 (BioXCell), at 10 mg/kg or agonistic anti-CD40, clone eFGK4.5 (BioXCell), at 5mg/kg intraperitoneally and analyzed 2/3 days after treatment, unless otherwise noted. In case of overall survival experiments, mice received anti-PD-1 on days 7, 14 and 21 of tumor growth. For CD8^+^ and CD4^+^ T cells depletion, mice received anti-CD8β clone 53–5.8 at 10 mg/kg or anti-CD4 clone GK1.5 at 5 mg/kg, respectively, (BioXCell) intraperitoneally one day before anti-PD-1 treatment and every 3 days thereafter.

##### Treg cells targeting

For systemic Treg depletion, *FoxP3*-iDtr-GFP (Jackson Labs Cat #016958) mice were implanted with MC38 cells as described above. To deplete Treg cells, diphtheria toxin (DT) was administered one day before and one day after immunotherapy treatment, on days 6 and 8 of tumor growth, respectively. For therapeutic Treg cells depletion, mice implanted with MC38 cells were treated with anti-CD25^NIB^ (mIgG2a, clone 7D4, S. Quezada laboratory; purchased from Evitria) or an isotype matched control (mIgG2a, clone C1.18.4, BioXCell) at 200 μg / mouse intraperitoneally one day before immunotherapy treatment. Tissues were collected and analyzed on day 9 or 10 of tumor growth. In case of overall survival experiments, mice received anti-PD-1 on days 7, 14 and 21 of tumor growth and anti-CD25^NIB^ one day before anti-PD-1 treatment.

##### DT injection

Mice receiving diphtheria toxin (DT; Sigma Aldrich) were dosed at 30 μg of DT per kg of body weight to initiate depletion 1 day before immunotherapy treatment and then every 2 days with 15 μg of DT per kg of body weight administered.

##### Bone marrow transfer experiments

CD45.1 recipient mice were irradiated with a single dose of 9 Gy using an Xstrahl CIX3 irradiator using a 1 mm thick copper filter. The next day, bone marrow was harvested from donor mice and processed for injection: either C57BL6/J (CD45.2), *Cd40*-KO or *Zbtb46*-Dtr mice served as donors. Cells from each type of donor were counted manually. For 50:50 bone marrow chimeras, cells were mixed at a 1:1 ratio before injection. Cells were injected intravenously at 10×10^6^ cells / mouse in 200 μl volume and mice were allowed to reconstitute for 6–7 weeks. Chimerism was confirmed by tail vein blood collection 1 week before tumor challenge.

##### Preparation of single cell suspensions, antibody staining and flow cytometry

Tumors were collected, minced into small pieces and treated with 50U/mL DNAse I (DCLS; Worthington) and 400U/mL collagenase IV (CLS-4; Worthington) for 20 minutes at 37°C under agitation. After enzymatic digestion, tumor fragments were passed through a 70μm cell strainer and cell suspensions were resuspended for staining in PBS. Prior to phenotypic analyses, dead cells were stained using the fixable viability dyes Zombie UV or Zombie Near IR (Biolegend) for 15 minutes at room temperature and concomitantly Fc receptors were blocked with TruStain fcx (Biolegend). Cell surface proteins analyses were performed by staining cells in FACS buffer (PBS supplemented with 2.5% FBS and 2mM EDTA) added with fluorochrome-conjugated antibodies to surface antigens for 20 minutes at room temperature. Cells were thereafter fixed and permeabilised with the FoxP3 transcription factor staining kit following manufacturer’s instructions (eBioscience) and stained for intracellular and nuclear proteins for 1 hour at room temperature. For the analyses of *Ccl22* mRNA, we used PrimeFlow following manufacturer’s instructions (Thermo Fisher). Flow cytometry data were acquired on a Symphony A5 instrument (BD Bioscience) and analyzed with FlowJo software (v10.5.3; BD Bioscience).

##### Isolation of tumor-resident DCs and ex vivo OT-I T cells stimulation

Tumor-derived cell suspensions were obtained as described above. For CCR7 receptor analyses only, cell suspensions were stained with anti-CCR7 antibody for 1 hour at 37°C in RPMI and then we proceeded to dead cell staining. After cell surface proteins staining, cells were passed through a 40 μm filter and FACS sorted on a FACSAria II SORP (BD Bioscience) or a MoFlo Astrios EQ (Beckman Coulter) sorter. DCs were sorted for live CD45^+^F4/80^−^Ly6G^−^CD3^−^MHC-II^+^CD11c^+^CCR7^+^ cells in 100 % FBS for cell culture or 70 μl RLT lysis buffer for RNA extraction following manufacturer’s instructions (RNeasy Micro Kit; Qiagen). For *ex vivo* stimulation of OT-I × CD45.1 T cells, FACS-sorted DCs were resuspended in T cell medium (RPMI 10 % FBS, 1 % GlutaMax) at a concentration of 3000–5000 cells/ml, placed in 96-U well plates and pulsed with 1 μg/ml OVA peptides (OVA_257–264_) for 30 minutes at 37°C. Spleens from naive OT-I × CD45.1 mice were passed through a 70 μm cell strainer and blood was lysed with RBC lysed buffer. CD8^+^ T cells were purified by immunomagnetic negative selection using the Miltenyi CD8^+^ T cells isolation kit and labelled with 2.5 μM CellTrace Violet in 5 ml of PBS for 15 minutes at 37°C. After washing, CD8^+^ T cells were incubated with DCs at 1:5 ratio (DCs:T cells) in 200 μl total volume of T cell medium in 96-U well plates and maintained for 5 days in culture.

#### Immunofluorescence staining procedure

Tumors and tumor-draining lymph nodes were washed in PBS, fixed with 4% paraformaldehyde for 2 to 4 hours, dehydrated with 20% sucrose in PBS overnight and finally embedded in OCT and preserved at −80°C. Tumor sections of 8 μm were prepared using a cryostat, permeabilized using 0.2% Triton X-100 in PBS for 5 minutes and blocked using a buffer containing 1% bovine serum albumin (BSA) and 10% FBS for 30 minutes. Sections were stained with primary antibodies and/or fluorochrome-conjugated antibodies diluted in blocking buffer overnight at 4°C in a dark humidified chamber and subsequently with fluorochrome conjugated secondary antibodies diluted in blocking buffer for 30 minutes. Finally DAPI staining was used to identify cell nuclei and sections were imaged using a slide scanner Axioscan 7 (Zeiss) or a confocal microscope Stellaris 8 (Leica). Confocal images are exclusively used to display representative field of views (FOVs).

#### Image analyses of immunofluorescence stainings

##### Tumor area quantification

Using QuPath,^101^ tumor boundaries were delineated using the magic wand tool on the DAPI signal, and the area of the resulting object was quantified.

##### BV and LV abundances in tumors

CD31^+^ and LYVE^+^ objects were generated across the entire tumors using the surface generation tool in IMARIS software (Oxford Instruments). BVs and LVs were defined as CD31^+^ surfaces with an overlapped area ratio to LYVE-1^+^ surfaces either below or above 0.01%, respectively. The total areas of BVs and LVs were quantified and normalized to the tumor area measured as described above.

##### CCR7^+^ DC abundance in tumors

FSCN1^+^ objects were generated across the entire tumors using the surface generation tool in IMARIS software (Oxford Instruments). The total area of CCR7^+^ DCs was quantified by summing the areas of all individual FSCN1^+^ surfaces and normalized to the tumor area measured as described above.

##### CCR7^+^ DC association to LVs vs BVs at whole tumor level

Distances border to border to closest BV and LV, as defined above, were compared for each CCR7^+^ DC surface located within 20μm of either a BV or LV. Two scenarios were distinguished: 1) a CCR7^+^ DC surface was classified as associated with a BV if its distance to the closest BV was shorter than its distance to the closest LV; 2) a CCR7^+^ DC surface was classified as associated with a LV if its distance to the closest LV was shorter than its distance to the closest BV. CCR7^+^ DC surfaces located more than 20 μm away from any vessel were classified as non-vessel-associated CCR7^+^ DCs.

##### CCR7^+^ DC clusters association to LVs vs BVs at whole tumor level

FSCN1^+^ structures were segmented by applying an artificial neural network pixel classifier at a resolution of 0.65 μm/pixel, focusing solely on the FSCN1 channel and using Gaussian and Laplacian of Gaussian features on Qupath. The pixel classifiers were trained using a training image composed of selected fields of view (FOVs) from several representative images, with at least one pixel classifier generated per experiment. The resulting segmentations were then divided into individual detections. FSCN1 structures with an area smaller than 5 μm^2^ were excluded. Subsequently, a Delaunay triangulation with an edge length of 22.5 μm was applied to cluster FSCN1 detections. The clusters were then manually curated to include all the detections with a border-to-border distance less than 20 μm, and exclude groups of cells with a total area below 500 μm^2^.

To assess the association of clusters with BV or LV, vessels near previously annotated CCR7^+^ DC clusters were manually segmented based on CD31, and CD31 and LYVE-1 markers, respectively. The border-to-border distance for each FSCN1 detection within the clusters to nearby BVs and LVs was calculated. A cluster was considered associated with a vessel if at least 50% of its area was within 20 μm of the nearest vessel. In the rare instances where a CCR7^+^ DC cluster is found to be associated with both a BV and a LV simultaneously, the vessel type it is linked to was determined through visual inspection.

##### CCR7^+^ DC cluster association to CCL21^+^ areas

CCL21 positive regions were manually annotated in entire tumors using the magic wand tool in QuPath. The segmentation of CCR7^+^ DC clusters and their association to BVs or LVs were performed as described above. Then, the distance from each FSCN1 detection within a CCR7^+^ DC cluster to the nearest CCL21^+^ region (calculated as the centroid-to-border distance) was determined. A CCR7^+^ DC cluster was considered to be within a CCL21^+^ region if at least one of its FSCN1 detection had a distance of 0.

##### Ccl19*-covered BVs abundance in tumors*

*Ccl19*-tomato^+^ eYFP^+^ objects were generated across the entire tumors using the surface generation tool in IMARIS software (Oxford Instruments). *Ccl19*-covered BVs were defined as BVs with an overlapped area ratio to *Ccl19*-tomato^+^ eYFP^+^ surfaces greater than 0%, while *Ccl19*-non-covered BVs were defined as BVs with an overlapped area ratio to *Ccl19*-tomato^+^ eYFP^+^ surfaces equal to 0%. The areas of all *Ccl19*-covered and non-covered BVs were summed and normalized to the total BV area measured as described above.

##### CCR7^+^ DC association to Ccl19-covered BVs/LVs

A CD31^+^ object, representing a single vessel, was generated using the surface generation tool in IMARIS software (Oxford Instruments). LYVE-1 status of the CD31^+^ object was visually assessed to classify the vessel as either a BV (LYVE-1^−^) or a LV (LYVE-1^+^). *Ccl19* coverage status of the vessel was defined as described above.

FSCN1^+^ objects located within 20μm border to border of the CD31^+^ surface, representing CCR7^+^ DCs associated with the vessel, were then generated. The areas of all individual FSCN1^+^ surfaces generated were summed to calculate the total area of CCR7^+^ DCs within the vessel niche. A vessel associated with a CCR7^+^ DC cluster was defined as a vessel with a total CCR7^+^ DC area greater than or equal to 500um^2^. In contrast, a vessel associated with few or any CCR7^+^ DC was defined as a vessel with a total CCR7^+^ DC area less than 500um^2^.

The full process was repeated to gather information on a representative fraction of tumor vessels.

##### CCR7^+^ DC clusters association to BV subtypes

BVs and their associated CCR7^+^ DC clusters were obtained as described just above. ACKR1 and ENCM status of the BV was visually assessed on consecutive sections. Venous BV were defined as ENCM^+^ ACKR1^+^, arterial BVs as ENCM^−^ ACKR1^−^ and capillaries as ENCM^+^ ACKR1^−^.

#### Volumetric Confocal microscopy

Volumetric confocal microscopy of tumors was performed as previously described.^102^ In brief, mouse tumors were fixed in Antigenfix (Diapath), dehydrated in 30% sucrose and embedded in TissueTek OCT freezing medium (Sakura Finetek). Embedded tumor samples were frozen and stored at −80°C. 50μm thick consecutive sections were generated using a CM3050 S cryostat (Leica). Sections were permeabilized, blocked and stained in 0.1M Tris (Carl Roth) containing 1% BSA, 0.3% Triton X-100 (Merck) and 1% normal mouse serum (Merck). Stained sections were mounted in Mowiol (Merck) and acquired with an inverted TCS SP8 confocal microscope (Leica) using a HC PL APO CS2 20x/0.75NA objective. All images were acquired as tiled Z-stacks spanning whole tumor sections in the XY plane with 1μm Z-spacing, providing large three-dimensional images of at least 20μm depth.

#### Analyses of volumetric confocal microscopy images

Image analysis was performed using the IMARIS software v9.7 (Oxford Instruments), as previously described.^102^ The IMARIS surface generation tool was used for cell rendering to visualise representative 3D objects for individual CCR7^+^ DCs, Treg, T_CONV_ and CD8^+^ T cells. FSCN1^+^ cells in the images were identified as CCR7^+^ DCs, Treg as CD4^+^FOXP3^+^ cells, T_CONV_ as CD4^+^FOXP3^−^ cells and CD8^+^CD4neg cells as CD8^+^ T cells. Interactions between CCR7^+^ DCs and T cell subsets were determined using the object-object shortest distance function in IMARIS. Two cells were considered to be interacting if the distance from a CCR7^+^ DC to the respective T cell was ≤ 5μm. CCR7^+^ DC clusters were defined as a minimum of 5 DCs within a distance of ≤ 20μm to each other, as identified by the IMARIS built-in “distance to 5 nearest neighbors” function. DC clusters with fewer than 5 cells were removed manually. For the enumeration of CCR7^+^ DC clusters per tumor and of CCR7^+^ DCs per cluster, the maximum cluster diameter was set to 100μm to allow for distinction of individual clusters in DC-rich tumor regions.

#### Preparation of mice for 2-photon intravital microscopy studies

*Il-12p40*-IRES*-*eYFP, *Il-12p40*-IRES*-*eYFP × *FoxP3*-IRES-mRFP or *Il-12p40*-IRES*-*eYFP × *FoxP3*-mRFP × *Ccl22*-KO mice were depilated on the back using Veet cream and 2×10^6^ MC38-H2B-Cerulean cells were injected subcutaneously in the right flank of each mouse, around 1 cm lateral to the midline of the back. 5 days post tumor inoculation, dorsal skin fold chambers (DSFC) were surgically implanted to encircle and contain the growing tumors.^36^ Intra- and preoperative anaesthesia was achieved using isofluorane inhalation. Mice received 1 mg/kg of buprenorfin (Temgesic) and antibiotics (Nopil) diluted in drinking water for 4 days. 2-photon intravital microscopy was started 5 days after the surgery and performed at multiple time points thereafter.

#### Intravital imaging

Mice bearing DSFC were anaesthetised with isofluorane and the DSFC were mounted on a custom-built stage. In order to visualise BVs, mice were injected intravenously with 40 nM QTracker 705 non-targeted quantum dots (Invitrogen) diluted in 100 μl sterile PBS before image acquisition. A Maitai and an Insight 3X lasers (Leica/Spectra Physics) were tuned for balanced excitation of Cerulean, EYFP, mRFP and QDots. Stacks of 12 to 20 optical sections (1094×1094 pixels) with a 5 μm spacing were acquired every 90 s to visualise imaging volumes of 60 to 100 μm in depth. Datasets were transformed in IMARIS 10 (Bitplane) to generate maximum intensity projections (MIPs) for export as MP4 movies.

#### Processing and analyses of 2-photon intravital microscopy recordings

MP-IVM recordings were analysed using IMARIS. Individual *Il-12p40*-IRES-eYFP- and *FoxP3*-IRES-mRFP-expressing DC and Treg cells, respectively, were identified based on fluorescence intensity and morphology of the 3D objects. Cellular migration was tracked based on automated track generation and manually refined. DCs were considered interacting with Treg cells when shortest distance between border surfaces was below 5 μm during the time of acquisition. Duration of interactions was calculated by using the time during which two surfaces were below 5 μm distance without being interrupted. Tracks speed (μm/s) was extracted from statistical data generated within IMARIS. To compute the cumulative DC-Treg interaction, the centroid-to-centroid distance between DCs and each Treg was calculated at each timeframe (one timeframe represents on average a time of 100s ± 7 seconds). One Treg was considered interacting with a DC if its distance to it was below 20μm. The number of interactions was calculated for each DC over time.

#### GO enrichment analyses

GO pathway enrichment analyses was performed using METASCAPE.^103^ For each comparison (*e.g.* CCR7^+^ DCs from Treg-depleted vs WT tumors), we used differentially expressed genes (DEGs) as a query.

#### Human studies

##### scRNAseq analyses

scRNA analysis was done using various functions in R package scalpi (https://www.gitlab.com/pwirapati/scalpi). For human data, the cell annotations from Bill et al.^62^ were used to train logistic regression classifier for DC subtypes cDC1_XCR1 → cDC1, cDC2_CD1A + cDC2/MoDC_CLEC10A → cDC2, DC_LAMP3 → CCR7 DC and pDC_LILRA4 → pDC. Simplified T cell subtypes were used by combining the respective minor states into CD8, CD4_Treg and CD4_Tconv. The classifier was then used to automatically annotate other human datasets. To quantify the average expression per cell subtypes, scalpi aggregated (pseudobulk) function is used to make per-patient summaries first (thus normalizing the cell counts per sample), before the patient-level summaries were averaged. Analogous process was done similarly for the combined mouse data. Manually identification into DC subtypes was done based on the heatmap. Visualization using hierarchical clustering heatmaps and UMAP dimensionality reduction were done using the respective functions in the scalpi analysis pipeline.

##### Multispectral Imaging of NSCLC and EC tumor samples

Four μm thick formalin-fixed paraffin-embedded (FFPE) tissue sections of NSCLC and EC tumor samples from 20 and 5 patients, respectively, were stained by multiplexed immunofluorescence (IF). All samples passed quality control and could be used for subsequent analyses. Multiplexed staining was performed on Ventana Discovery Ultra staining module (Ventana, Roche, Serial #313236) using a preset protocol. Slides were baked at 60°C for 1 hour, placed on the staining module for deparaffinization (Discovery Wash, Roche, Cat #950–510), and in high pH for 64 min at 95°C for epitope retrieval (Discovery CC1, Roche, Cat #950–500). Multiplex staining consisted in multiple rounds of staining. Each round included endogenous peroxidase quenching (Discovery Inhibitor, Roche, Cat #760–4840), non-specific sites blocking for 8 minutes (Discovery Goat IgG and Discovery Inhibitor, Roche, Cat #760–6008), primary antibody incubation, secondary HRP-labeled antibody incubation for 16 minutes (OmniMap anti-rabbit HRP, Roche, Cat# 760–4311) or (OmniMap anti-mouse HRP, Roche, Cat#760–4310)), OPAL^™^ reactive fluorophore detection (Akoya Biosciences, Marlborough, MS, USA) that covalently label the primary epitope and then antibodies heat denaturation (Discovery CC2, Roche, ref # 950–223). Sequence of antibodies used in the multiplex with the associated OPAL for NSCLC and EC respectively are the following:

- 1^st^: Rabbit anti-CD4 1h, 37°C (Cat# 104R-16, Cellmarque, diluted 1/100), OPAL690; 2^nd^: Mouse monoclonal anti-CD11c 1h at 37°C (Cat# 111M-15, Cellmarque, diluted 1/50), OPAL570; 3^rd^: Rabbit monoclonal anti-FoxP3 1h at 37°C (Cat# 99963, abcam, diluted 1/400), OPAL520; 4^th^: Mouse monoclonal anti-CD31, 1h at room temperature (Cat# CM347A, Biocare, diluted 1/1000), OPAL620; 5^th^: Mouse monoclonal anti-Cytokeratin, 1h at 37°C (Cat# M3515, Dako, diluted 1/100), OPAL480; 6^th^: Rabbit monoclonal anti-LAMP3, 1h at room temperature (Cat# 108R-16, Atlas Antibodies, diluted 1/250), OPAL780.
- 1^st^: Mouse monoclonal anti-CD31, 1h at room temperature (Cat# CM347A, Biocare, diluted 1/1000), OPAL520; 2^nd^: Mouse monoclonal anti-CD11c 1h at 37°C (Cat# 111M-15, Cellmarque, diluted 1/50), OPAL620; 3^rd^: Rabbit monoclonal anti-LAMP3, 1h at room temperature (Cat# 108R-16, Atlas Antibodies, diluted 1/250), OPAL690; 4^th^: Mouse monoclonal anti-Cytokeratin, 1h at 37°C (Cat# M3515, Dako, diluted 100), OPAL480; 5^th^: Mouse monoclonal anti-PDPN, 1h at room temperature (Cat# 916605, Biolegend, diluted 1/250), OPAL780.

Nuclei were visualised by final incubation with Spectral DAPI (1/10, Akoya Biosciences, Cat #FP1490) for 12 minutes. The slides were mounted with Fluorescence mounting medium (Cat #S3023, Dako), stored in the dark at 4°C and scanned within 48 hours.

##### Multispectral Imaging and Data analyses of NSCLC and EC tumor samples

Multiplex immunofluorescence (IF) images were acquired at 20x and 40x on the Vectra^®^ Polaris automated quantitative pathology imaging system (Akoya Biosciences, Marlborough, USA), allowing the unmixing of spectrally overlapping fluorophores and tissue autofluorescence of whole slide scans. For the optimal IF signal unmixing (individual spectral peaks) and the subsequent multiplex analysis, a spectral library containing the individual emitting spectral peaks of all the 7 fluorophores were created and validated using the inForm Analysis software (Akoya Biosciences). The phenotyping analysis was performed using inForm 2.5.1 image analysis software (Akoya Biosciences) enabling a per-cell analysis of weak and/or spectrally overlapping IF markers of multiplex-stained tissue sections. The images were first segmented into specific tissue categories of tumor, stroma and no tissue, based on the CK and DAPI staining using the inForm Tissue Finder^™^ algorithms.Then, individual cells were segmented using the counterstained-based on adaptive cell segmentation algorithm. Quantification of the immune cells was finally performed using the inForm active learning phenotyping algorithm by assigning the different cell phenotypes across several images representative of the whole scan: CCR7^+^ DCs (defined as LAMP3^+^CD11c^+^panCK^−^; in both NSCLC and EC tumors), BECs (CD31^+^PDPN^−^; in EC tumors), LECs (PDPN^+^; in EC tumors), Treg (CD4^+^FOXP3^+^; in NSCLC tumors), tumor cells (panCK^+^; in both NSCLC and EC tumors). InForm software was trained to recognize cell phenotypes according to combination of probes/Ab described above. The output csv files, containing cellular coordinates, mean fluorescent intensities of each channel, and cellular phenotypes, were loaded in R for downstream analyses. In-house spatial analysis package *spati* [https://gitlab.com/pwirapati/spati] was used to generate digital reconstruction of regions of interests using the function *spati_plot()*. The function *d2_nearest*, following a square root calculation, was used to calculate the nearest distances from each annotated CCR7^+^ DCs to other classified cells.

##### FAST-FFPE sample preparation of HNSCC tumor samples

FFPE tissue sections of HNSCC was cut at 5 μm thickness and stored at −80°C. To prepare for imaging, FFPE tissue sections were baked at 55°C overnight on a slide warmer (Newcomer Supply), de-paraffinized in xylene, rehydrated in a series of graded alcohols, and washed in water. Tissue sections were then placed in Tris-based antigen unmasking solution (Vector Laboratories) in a de-cloaking chamber (Biocare Medical) for antigen retrieval. Tissue sections were boiled at 110°C under full pressure for 1 min, cooled to 80°C, and incubated in water (5 min, 2 times) and then in PBS (5 min). FFPE tissue sections were blocked with Intercept buffer (LI-COR Biosciences) for 30–60 minutes and permeabilized with 0.1% Triton X-100 for 30 min. A microfluidic tape device was installed around the tissue on the glass slide to allow efficient fluid exchange under cover slip for repeated immunostaining for FAST imaging.^104^

##### FAST cycling imaging of HNSCC tumor samples

FAST multiplex cyclic imaging was performed on HNSCC tissue section samples. Carrier-free antibodies against target proteins were purchased and conjugated to FAST probes^105^ as follows. Antibodies were exchanged into bicarbonate buffer (pH 8.4) using a 40k Zeba column (Thermo Fisher) and incubated with an appropriate molar excess of the Fluorophore-TCO-NHS molecule (FAST probe) with 10% DMSO for 25 mins at room temperature. The conjugation reaction was loaded onto a 40k Zeba column equilibrated with PBS for removal of unreacted FAST probes. Antibodies with the degree of labeling of 2–4 were used. The FAST-labeled antibodies were stored in the dark at 4°C in PBS. All antibody conjugates were validated using human tonsil tissue sections. Immunostaining for FAST imaging was performed as typical immunofluorescence protocol. The FAST-conjugated antibodies were diluted to 1–5 μg/ml in Intercept buffer for staining. In each cycle, tissue sections were incubated with antibodies for 2 hours for surface markers or overnight for intracellular/nuclear markers, and then washed with PBS for 10 minutes 3 times. Following image acquisition, fluorescent signal was quenched by treating tissue sections with 10 μM Tz-BHQ for 30 minutes in PBS-bicarbonate buffer. Tz-BHQ solution was removed by three washes with PBS, and the quenched signal was imaged in the same FOVs. Tissue sections were then incubated in a solution of 10 μM dTCO-PEG6-CO2H in order to block any residual Tz-BHQ3 from reacting with FAST antibodies of the next cycle. The same staining, imaging, and quenching cycle was repeated to image all target proteins.

Each tissue section was scanned by slide scan module in Metamorph, using Olympus BX-63 upright epifluorescence microscope (20x objective resulting in a FOV of 561.6 × 561.6 μm). We investigated 2370 separate FOVs typically allowing for the coverage of 10–100 mm^2^ of each tumor for spatial analyses. The light source was a X-Cite XYLIS system (Excelitas, Mississauga, ON, Canada), and the imaging camera was an ORCA-Fusion (Hamamatsu). The following filters were used for four imaging channels: Hoechst: OSF3-DAPI-1160B-Z (Ex: 387/11; Em: 447/60nm); AF488: OSF3-GFP-3035D-Z (Ex: 472/30; Em: 520/35 nm); AF555: OSF3-CY3–4040C-Z (Ex: 531/40; Em: 593/40nm); AF647: OSF3-CY5–4040B-Z (Ex: 628/40; Em: 692/40 nm).

##### High-plex whole-tissue imaging and image acquisition of HNSCC human sections

FFPE tissue sections of HNSCC were cut at 5 μm thickness and stored at −80°C. Prior to imaging, sections were incubated at 55°C overnight (HybEZ ^™^Oven ACD, BioTechne), de-paraffinized (HistoChoice Clearing Agent,Sigma Aldrich) for 2×10 min, rehydrated for 5 min in a series of graded alcohols (100%, 100%, 90%, 70%, 50%, 30%) and then washed twice 5 min in distilled water. Antigen retrieval was performed using a pressure cooker for 20 min (high pressure) at pH9(AR9 buffer, Akoya Biosciences). Tissue sections were cooled at room temperature for 2 hours and washed twice in distilled water. Finally, sections were immersed in Hydration Buffer (Akoya Biosciences) for 2 min twice and equilibrated in Staining Buffer (Akoya Biosciences) for 30 min. Sections were then incubated in antibody cocktail containing N, J S PUBLISHING and G blockers (Akoya Biosciences) at 4°C overnight. Antibodies were either directly purchased from Akoya Biosciences or conjugated to oligo barcodes according to Manufacturer’s instructions (Akoya Biosciences). Post-staining procedure, reporter plate preparation, flow cell assembly and imaging was performed following the Manufacturer’s instructions. Upon image acquisition, a high resolution QPTIFF file containing a composite image of all markers was generated and imported into the QuPath (v0.5.1) software (https://qupath.github.io/) for manual quality assessment. Image processing was conducted using the Sopa pipeline^106^ with Cellpose^107^ as the segmentation algorithm using the DAPI channel.

##### Cell typing of high-plex whole tissue imaging of HNSCC human sections

Cell phenotyping was performed manually on three HNSCC samples (PI001, PI011, PI012). Cells were classified as follows: tumor cells (Pan-Cytokeratin^+^), BVs (CD31^+^), LVs (Podoplanin^+^), CCR7^+^ DCs (LAMP3^+^ and HLA-DR^+^), Treg (FOXP3^+^). Gates for each marker were manually adjusted based on their standard deviation distributions, except for the LAMP3 marker, where the sum distribution was used. Phenotypes were visually validated using QuPath. Manually curated phenotypes were subsequently used to train a Random Forest classifier, which was applied to predict cell phenotypes in the remaining six samples. The classifier was trained on the full marker standard deviation expression matrix.

##### Spatial-omics analyses of high-plex whole tissue imaging of HNSCC human sections

Downstream analysis was restricted to tumor-adjacent areas using ‘*spati*’ (https://gitlab.com/pwirapati/spati), an in-house R package designed for spatial data processing. The tumor regions computed by the ‘*spati*’ function were further extended by an additional 10 cell layers, as described in Bill et al. (2023).^62^ This extension ensured the inclusion of peritumoral regions for more comprehensive spatial analysis.

##### CCR7^+^ DCs association to LVs and BVs

CCR7^+^ DCs located at a distance greater than 50 μm border to border (FAST cycling imaging) or 100 μm centroid to centroid (multispectral imaging) were classified as non-vessel-associated CCR7^+^ DCs. For each of all the other CCR7^+^ DCs, representing vessel-associated CCR7^+^ DCs, distances to closest BV/BEC and LV/LEC were compared. Two scenarios were distinguished: 1) a CCR7^+^ DC was classified as associated with a BV if its distance to the closest BV/BEC was shorter than its distance to the closest LV/LEC; 2) a CCR7^+^ DC surface was classified as associated with a LV if its distance to the closest LV/LEC was shorter than its distance to the closest BV/BEC.

#### Public spatial transcriptomic data

##### Cell typing for new cell annotation

We downloaded Vizgen’s MERSCOPE FFPE Human Immuno-oncology dataset,^77^ which contains single-cell MERFISH spatial transcriptomics data from 16 samples across 8 different tumor types, using a panel of 500 genes. 14 out of the 32 genes of the CCR7^+^ DC signature overlapped with the dataset panel. These genes were used to score all cells in the dataset using Scanpy’s *tl.score_genes* function.^108^ The distribution of scores followed a bimodal distribution with a separation between the two modes at 0.5. From the four samples with the highest number of cells scoring above 0.5, we selected HumanLungCancerPatient2 (1983 cells), HumanColonCancerPatient2 (1243 cells), and HumanUterineCancerPatient1 (979 cells) due to the availability of high-quality reference datasets for cell typing. We performed cell typing for each of the three query samples individually using a seed labeling procedure. We performed dimensionality reduction using scVI,^109^ and clustering using Scanpy’s Leiden algorithm with *resolution = 2*. Clusters that could be confidently annotated based on their marker genes were labeled with the corresponding cell types. We selected the 500 genes in the reference dataset that overlapped with the MERFISH panel and used scANVI^110^ to perform semi-supervised transfer of the annotations from the reference dataset to the query sample based on the partial cell labels. The reference datasets used were the extended single-cell lung cancer atlas (LuCA)^111^ for HumanLungCancerPatient2, the Colorectal Cancer Atlas^112^ for HumanColonCancerPatient2, and the CellTypist Organ Atlas^113^ for HumanUterineCancerPatient1. The resulting cell type annotations were refined through manual curation.

##### Neighborhood enrichment analyses for new cell annotation

We identified the CCR7^+^ DCs in each sample individually. Cells were scored using the CCR7^+^ signature and Scanpy’s *tl.score_genes* function. The distribution of scores followed a bimodal distribution with a separation at 1.5 for all three samples. Cells labeled as cDCs with scores above 1.5 were classified as CCR7^+^ DCs. The remaining cDCs were classified as CCR7^−^ DCs. For each sample, we computed the spatial network using Squidpy’s *gr.spatial_neighbors* function with Delaunay triangulation.^114^ We removed edges with distances above the 99th percentile of all edge distances. Neighborhood enrichment was computed using CellCharter’s implementation of asymmetric analytical neighborhood enrichment without intra-cluster edges.^115^

##### Analyses of CCR7^+^ DC in NSCLC datasets

We used MERFISH spatial transcriptomics data from four NSCLC patients, with cell identities pre-annotated by the original authors.^47^ Among the four annotated DC states, we identified a high CCR7^+^ DC signature in both the “LAMP3^+^CCL19^+^ mreg DC” and “LAMP3^+^CD1C^+^ DC” populations. For subsequent analyses, we reclassified these subgroups as CCR7^+^ DCs based on their signature profiles.

To assess spatial relationships between CCR7+ DCs and vessels, we performed nearest-neighbor distance analyses between CCR7^+^ DCs and arterial, venous, and lymphatic endothelial cells using the d2_nearest() function from the spati package (https://gitlab.com/pwirapati/spati).^62^ The visualization of representative field of interest was made using spati_plot()function from the spati package. CCR7^+^ DC association to either BVs or LVs was then performed as explained above (**CCR7**^**+**^ **DCs association to BVs vs LVs**). Importantly, distance to closest BEC was selected as the lower distance between arterial endothelial cell and venous endothelial cell.

To assess whether *CCL19*^+^ fibroblasts preferentially localized around BV-associated CCR7^+^. DCs, we performed a permutation-based neighborhood analysis using a custom spatial randomizing strategy. For each patient sample, the spatial enrichment of *CCL19*^+^ fibroblasts in CCR7^+^ DC local neighborhood (radius = 5 cell layers) was quantified usingthe nh_sum() function from spati package (https://gitlab.com/pwirapati/spati).^62^ To evaluate whether this enrichment exceeded randomization, we performed 1,000 permutations by randomly shuffling the cell type labels of all non-selected cells (excluding the BV-associated CCR7^+^ DCs). For each iteration, we recalculated the mean densities of *CCL19*^+^ fibroblasts within the neighborhood of the selected BV-associated CCR7^+^ DCs. Enrichment scores were calculated for each sample by normalizing the mean neighborhood densities to the sample-level baseline density of *CCL19*^+^ fibroblasts.

To identify cellular enrichment in different DC neighborhoods in each dataset, we used a cell–neighborhood matrix (Y), determined by nh_sum function within in-house built spati package (https://gitlab.com/pwirapati/spati), in which each row corresponds to a cell and each column to its specific neighborhood type; the *“all”* column denotes the total number of cells in that neighborhood. Cells were classified as three classes of DCs (DCs): CCR7^+^ DCs, CD1C^+^ITGAX^+^ DCs and FLT3^+^ DCS. For each DC group, neighborhood counts were summed across all cells and normalized to the *“all”* column to obtain relative neighborhood frequencies. A pseudocount of 0.001 was added prior to normalization to avoid division by zero. Ratios of normalized frequencies between selected DCs subtype and all other cells were calculated for each neighborhood type. This approach measures the relative enrichment or depletion of specific neighborhood types for DCs subtypes compared with the rest of the cellular population. Treg enrichment was further normalized to display differences among DC neighborhoods.

### QUANTIFICATION AND STATISTICAL ANALYSIS

Statistical analyses were performed using either GraphPad Prism software (v10) or R statistical software (https://www.R-project.org). Statistical significance was at the level of *p*<0.05, with appropriate multiple testing applied depending on the context. Box and whisker plots display the median, minimum and maximum values. Other details of statistical methods for specific analyses are described in figure legends.

## Supplementary Material

Supplemental Figures

SUPPLEMENTAL INFORMATION

Supplemental information can be found online at https://doi.org/10.1016/j.immuni.2025.11.020.

## Figures and Tables

**KEY RESOURCES TABLE:** 

REAGENT or RESOURCE	SOURCE	IDENTIFIER

Antibodies		

PerCP-Cy5.5 rat anti-mouse CD45 (Clone 30-F11)	BioLegend	Cat#103132;RRID: AB_893340
PB rat anti-mouse CD45 (Clone 30-F11)	BioLegend	Cat#103126;RRID: AB_493535
AF700 rat anti-mouse CD45.2 (Clone 104)	BioLegend	Cat#109821;RRID: AB_493730
PerCP-Cy5.5 rat anti-mouse CD44 (Clone IM7)	BioLegend	Cat#103031;RRID: AB_2076206
BV605 rat anti-mouse CD44 (Clone IM7)	BioLegend	Cat#103047;RRID: AB_2562451
BV650 rat anti-mouse F4/80 (Clone BM8)	BioLegend	Cat#123149;RRID: AB_2564589
AF647 rat anti-mouse Ly6G (Clone 1A8)	BioLegend	Cat#127609;RRID: AB_1134162
PB rat anti-mouse Ly6C (Clone HK1.4)	BioLegend	Cat#128014;RRID: AB_1732079
PE-Cy7 rat anti-mouse CD3 (Clone 17A2)	BioLegend	Cat#100220;RRID: AB_1732057
BV510 rat anti-mouse CD19 (Clone 6D5)	BioLegend	Cat#115546;RRID: AB_2562137
AF700 rat anti-mouse MHC-II (Clone M5/114.15.2)	BioLegend	Cat#107622;RRID: AB_493727
BV711 armenian hamster anti-mouse CD11c (Clone N418)	BioLegend	Cat#117349;RRID: AB_2563905
BV421 rat anti-mouse CCR7 (Clone 4B12)	BioLegend	Cat#120120;RRID: AB_2561446
AF488 rat anti-mouse CCR7 (Clone 4B12)	BioLegend	Cat#120110;RRID: AB_492841
BV785 rat anti-mouse XCR1 (Clone ZET)	BioLegend	Cat#148225;RRID: AB_2783119
BV605 rat anti-mouse CD11b (Clone M1/70)	BioLegend	Cat#101237;RRID: AB_11126744
APC-Cy7 rat anti-mouse CD11b (Clone M1/70)	BioLegend	Cat#101225;RRID: AB_830641
PE/Dazzle594 rat anti-mouse SIRPα (Clone P84)	BioLegend	Cat#144016;RRID: AB_2565280
PE rat anti-mouse CD40 (Clone 3/23)	BioLegend	Cat#124610;RRID: AB_1134075
PerCP-Cy5.5 armenian hamster anti-mouse CD80 (Clone 16-10A1)	BioLegend	Cat#104722;RRID: AB_2291392
APC-Cy7 rat anti-mouse CD8α (Clone 53-6.7)	BioLegend	Cat#100714;RRID: AB_312753
PE/Dazzle594 rat anti-mouse CD25 (Clone PC61)	BioLegend	Cat#102048;RRID: AB_2564124
PE mouse anti-mouse H-2Kb bound to SIINFEKL (Clone 25-D1.16)	BioLegend	Cat#141603;RRID: AB_10897938
BUV805 rat anti-mouse CD45 (Clone 30-F11)	BD Biosciences	Cat#568336;RRID: AB_3684191
PE-Cy7 mouse anti-mouse CD45.1 (CloneA20)	BD Biosciences	Cat#560578;RRID: AB_1727488
BUV737 rat anti-mouse F4/80 (Clone T45-2342)	BD Biosciences	Cat#749283;RRID: AB_2873658
BUV395 rat anti-mouse Ly6G (Clone 1A8)	BD Biosciences	Cat#565964;RRID: AB_2716852
BUV805 rat anti-mouse CD3 (Clone 17A2)	BD Biosciences	Cat#741982;RRID: AB_3684854
BV711 rat anti-mouse CD90.2 (Clone 53-2.1)	BD Biosciences	Cat#740647;RRID: AB_2740336
BUV395 rat anti-mouse CD40 (Clone 3/23)	BD Biosciences	Cat#745697;RRID: AB_2743179
BV650 rat anti-mouse CD86 (Clone GL1)	BD Biosciences	Cat#564200;RRID: AB_2738665
BUV737 rat anti-mouse CD4 (Clone GK1.5)	BD Biosciences	Cat#612761;RRID: AB_2870092
BUV496 rat anti-mouse CD4 (Clone GK1.5)	BD Biosciences	Cat#612952;RRID: AB_2813886
BUV737 rat anti-mouse PD1 (Clone RMP1-30)	BD Biosciences	Cat#568363;RRID: AB_3684212
PE-Cy7 rat anti-mouse PDL1 (Clone MIH5)	eBioscience	Cat#25-5982-80;RRID: AB_2573509
AF488 rat anti-mouse CD8α (Clone 53-6.7)	eBioscience	Cat#53-0081-82;RRID: AB_469897
PE rat anti-mouse FOXP3 (Clone FJK-16s)	eBioscience	Cat#12-5773-82;RRID: AB_465936
APC rat anti-mouse FOXP3 (Clone FJK-16s)	eBioscience	Cat#17-5773-82;RRID: AB_469457
BV605 rat anti-mouse Ki67 (Clone SolA15)	eBioscience	Cat#406-5698-82; RRID: AB_2937179
AF700 rat anti-mouse FOXP3 (Clone FJK-16s)	eBioscience	Cat#56-5773-82;RRID: AB_1210557
AF594 rabbit anti-mouse TCF7 (Clone C63D9)	Cell Signaling Technology	Cat#35972
PE rat anti-mouse IL-12/IL-23 p40 (Clone C17.8)	eBioscience	Cat#12-7123-81;RRID: AB_466184
BUV737 mouse anti-mouse NK1.1 (Clone PK136)	eBioscience	Cat#741715; RRID: AB_2871088
PE-Cy7 armenian hamster anti-mouse CD103 (Clone 2E7)	BioLegend	Cat#121426; RRID: AB_2563691
AF647 mouse anti-mouse FSCN1 (Clone 55K-2)	Santa Cruz Biotechnology	Cat#sc-21743 AF647; RRID: AB_627580
AF488 mouse anti-mouse FSCN1 (Clone 55K-2)	Santa Cruz Biotechnology	Cat#sc-21743 AF488; RRID: AB_627580
AF546 mouse anti-mouse FSCN1 (Clone 55K-2)	Santa Cruz Biotechnology	Cat#sc-21743 AF546; RRID: AB_627580
AF594 mouse anti-mouse FSCN1 (Clone D-10)	Santa Cruz Biotechnology	Cat#sc-46675 AF594; RRID: AB_627582
AF488 rabbit anti-GFP (polyclonal)	Invitrogen	Cat#A21311; RRID: AB_221477
AF700 rat anti-mouse FOXP3 (Clone MF-14)	BioLegend	Cat#126422; RRID: AB_2750493
BV421 rat anti-mouse CD8α (Clone 53-6.7)	BioLegend	Cat#100738;RRID: AB_11204079
BV510 rat anti-mouse CD31 (Clone MEC 13.3)	BD Biosciences	Cat#563089;RRID: AB_2737997
AF647 rat anti-mouse CD4 (Clone GK1.5)	BioLegend	Cat#100424;RRID: AB_389324
Unconjugated rat anti-mouse FOXP3 (Clone FJK-16s)	eBioscience	Cat#14-5773-82;RRID: AB_467576
Unconjugated chicken anti GFP (polyclonal)	Aves Labs	Cat#GFP-1010;RRID: AB_2307313
Unconjugated goat anti-mouse CD31 (polyclonal)	R&D Systems	Cat#AF3628; RRID: AB_2161028
Unconjugated rat anti-mouse CD31 (Clone MEC 7.46)	Novus Biologicals	Cat#NB100-1642SS; RRID: AB_11014151
Unconjugated rat anti-mouse EMCN (Clone V.7C7)	eBioscience	Cat#14-5851-82;RRID: AB_891527
Unconjugated goat anti-mouse αSMA (polyclonal)	Novus Biologicals	Cat#NB300-978; RRID: AB_2273628
Unconjugated goat anti-mouse (polyclonal)	Relia Tech	Cat#103-PA50S
Unconjugated goat anti-mouse CCL21 (polyclonal)	R&D Systems	Cat#AF457; RRID: AB_2072083
Unconjugated rat anti-mouse CD31 (Clone MEC 13.3)	BD Biosciences	Cat#550274;RRID: AB_393571
Unconjugated rat anti-mouse CD31 (Clone EPR17260-263)	Abcam	Cat#ab222783; RRID: AB_2905525
Unconjugated rat anti-mouse ACKR1 (Clone 6B7)	Lab of U. von Andrian	PMID: 28526034
AF488 rat anti-mouse MECA-79 (Clone MECA-79)	Invitrogen	Cat#53-6036-82;RRID: AB_10804391
Unconjugated mouse anti-mCherry	Takara	Cat#632543; RRID: AB_2307319
Unconjugated goat anti-mouse Desmin (polyclonal)	eBioscience	Cat#PA5-19063;RRID: AB_10977860
AF488 donkey anti-rabbit IgG (polyclonal)	Invitrogen	Cat#A21206;RRID: AB_2535792
DyLight755 donkey anti-rat IgG (polyclonal)	Invitrogen	Cat#SA5-10031;RRID: AB_2556611
AF555 donkey anti-goat (polyclonal)	Invitrogen	Cat#A21432;RRID: AB_2535853
DyLight755 donkey anti-goat IgG (polyclonal)	Invitrogen	Cat#SA5-10091;RRID: AB_2556671
AF647 donkey anti-goat IgG (polyclonal)	Invitrogen	Cat#A21447;RRID: AB_2535864
AF488 donkey anti-chicken IgY (polyclonal)	Invitrogen	Cat#A78948;RRID: AB_2921070
AF555 donkey anti-rabbit IgG (polyclonal)	Invitrogen	Cat#A32974;RRID: AB_2762834
DyLight755 donkey anti-rabbit IgG (polyclonal)	Invitrogen	Cat#SA5-10043;RRID: AB_2556623
AF647 goat anti-rat IgG (polyclonal)	Invitrogen	Cat#A21247;RRID: AB_141778
AF488 donkey anti-goat IgG (polyclonal)	Invitrogen	Cat#A32814TR;RRID: AB_2866497
AF488 donkey anti-rat IgG (polyclonal)	Invitrogen	Cat#A21208;RRID: AB_2535794
Unconjugated rabbit anti-human LAMP3 (polyclonal)	Atlas Antibodies	Cat#HPA051467;RRID: AB_2681475
Unconjugated mouse anti-human CD31 (Clone BC2)	Biocare Medical	Cat#CM347A
Unconjugated rabbit anti-human FOXP3 (Clone SP97)	Abcam	Cat#ab99963; RRID: AB_10675258
Unconjugated mouse anti-human CD11c (Clone 5D11)	Cell Marque	Cat#111M-15
Unconjugated rabbit anti-human CD4 (Clone SP35)	Cell Marque	Cat#104R-16
Unconjugated mouse anti-human panCK (Clone AE1/AE3)	Agilent Dako	Cat#M3515
purified mouse anti-human PDPN (Clone D2-40)	BioLegend	Cat#916605;RRID: AB_2565820
Purified mouse anti-human panCK (Clone AE1/AE3)	BioLegend	Cat#914204;RRID: AB_2616960
Purified mouse anti-human CD68 (Clone KP1)	BioLegend	Cat#916104;RRID: AB_2616797
Purified mouse anti-human CD66b (Clone G10F5)	BioLegend	Cat#305102;RRID: AB_314494
Unconjugated rabbit anti-human CD45 (Clone D9M8I)	Cell Signaling Technology	Cat#47937SF; RRID: AB_
Unconjugated rabbit anti-human CD8α (Clone D8A8Y)	Cell Signaling Technology	Cat#85336
Unconjugated rabbit anti-human CD11b (Clone D6X1N)	Cell Signaling Technology	Cat#49420; RRID: AB_2799357
Unconjugated rabbit anti-human CD14 (Clone D7A2T)	Cell Signaling Technology	Cat#43878; RRID: AB_3683725
Unconjugated rabbit anti-human CD11c (Clone D3V1E)	Cell Signaling Technology	Cat#45581; RRID: AB_2799286
Unconjugated rat anti-human CD3 (Clone CD3-12)	Abcam	Cat#ab11089; RRID: AB_1889189
Unconjugated rabbit anti-human CD4 (Clone EPR6855)	Abcam	Cat#ab280849
Unconjugated mouse anti-human FOXP3 (Clone 236A/E7)	eBioscience	Cat#14-4777-80;RRID: AB_467555
Purified mouse anti-human PDPN (Clone D2-40)	BioLegend	Cat#916606;RRID: AB_2565820
Unconjugated mouse anti-human αSMA (Clone 1A4)	Invitrogen	Cat#14-9760-80;RRID: AB_2572995
Unconjugated rabbit anti-human ACKR1 (polyclonal)	LS Bio	Cat#LS-B2306-0.05; RRID: AB_2572995
Unconjugated rat anti-human MECA-79 (Clone MECA-79)	Novus Biologicals	Cat# NB100-77673SS
AF555 rabbit anti-human CD45 (Clone AKYP0074)	Akoya Biosciences	Cat#240060
AF750 mouse anti-human panCK (Clone AKYP0053)	Akoya Biosciences	Cat#S6501000
AF647 rabbit anti-human CD4 (Clone AKYP0048)	Akoya Biosciences	Cat#S6501002
AF647 mouse anti-human FOXP3 (Clone AKYP0102)	Akoya Biosciences	Cat#S6501007
AF550 mouse anti-human CD8 (Clone AKYP0028)	Akoya Biosciences	Cat#S6501001
AF647 rabbit anti-human CD11c (Clone AKYP0147)	Akoya Biosciences	Cat#200074
AF750 rabbit anti-human LAMP3 (polyclonal)	Sigma	Cat#HPA051467; RRID: AB_2681495
AF750 rabbit anti-human CD31 (Clone AKYP0047)	Akoya Biosciences	Cat#232172
AF550 rat anti-human PDPN (Clone AKYP0007)	Akoya Biosciences	Cat#240203

Biological samples		

Human HNSCC FFPE samples	MGH	This study
Human NSCLC FFPE samples	MGH	This study
Human endometrial FFPE samples	HUG	This study

Chemicals, peptides, and recombinant proteins		

Collagenase, Type 4	Worthington	Cat#CLS-4
DNASE I recombinant	Worthington	Cat#DCLS
Qtracker 705 Vascular Labels	Invitrogen	Cat#Q21061MP
TruStain FcX	BioLegend	Cat#101320
RBC Lyses Buffer 10X	BioLegend	Cat#420301
OVA 257-264 H-2Kb-restricted OVA MHC class I epitope	InvivoGen	Cat#vac-sin
Paraformaldehyde 32% Aqueous Solution EM Grade	EMS	Cat#15714
Triton X-100	Sigma Aldrich	Cat#X100-100ML
TissueTek OCT freezing medium	Sakura Finetek	Cat#4583
Antigenfix	Diapath	Cat#P0016
DAPI	ThermoFisher Scientific	Cat#D21490
Diphteria Toxin	Sigma Aldrich	Cat#D0564-1MG
Bovine Serum Albumin	Sigma Aldrich	Cat#A9418
InVivoMAb anti-mouse anti-PD-1 (clone 29F.1A12)	BioXcell	Cat#BE0273
InVivoMAb anti-mouse CD40 (clone FGK45)	BioXcell	Cat#BE0016-2
InVivoMAb mouse IgG2a isotype control (clone C1.18.4)	BioXcell	Cat#BE0085
Anti-CD25NIB (mIgG2a, clone 7D4)	Evitria	Solomon et al.^86^
InVivoMAb rat anti-mouse CD8β (Clone 53-5.8)	BioXCell	Cat#BE0223; RRID: AB_1107636
InVivoMAb rat anti-mouse CD4 (Clone GK1.5)	BioXCell	Cat#BE0003-1; RRID: AB_2687706
HistoChoice Clearing Agent	Sigma Aldrich	Cat#H2779
AR9 Buffer	Akoya Biosciences	Cat#AR9001KT

Critical commercial assays		

CD8a+ T Cell Isolation Kit	Miltenyi	Cat#130-104-075
Zombie UV Fixable Viability Kit	BioLegend	Cat#423108
Zombie near IR Fixable Viability Kit	BioLegend	Cat#423106
Foxp3 / Transcription Factor Staining Buffer Set	ThermoFisher Scientific	Cat#00-5523-00
CellTrace Violet Cell Proliferation Kit	ThermoFisher Scientific	Cat#C34557
Precision Counting Beads	BioLegend	Cat#424902
UltraComp eBeads^™^ Compensation Beads	ThermoFisher Scientific	Cat#01-2222-42
RNeasy Micro Kit	Qiagen	Cat#74004
PrimeFlow^™^ RNA Assay Kit	ThermoFisher Scientific	Cat#88-18005-204
PhenoCycler Staining Kit	Akoya Biosciences	Cat#7000008

Deposited data		

NSCLC MERFISH data	Chen et al.^47^	https://zenodo.org/records/11198494
MERSCOPE FFPE Human Immuno-oncology dataset	Vizgen	https://vizgen.com/human-ffpe-immunooncology-release-roadmap/
Human HNSCC scRNAseq and CITEseq dataset (1)	Bill et al.^62^	GSE234933
Human HNSCC scRNAseq dataset (2)	Franken et al.^63^	EGA50000000033
Human HNSCC scRNAseq dataset (3)	Kürten et al.^64^	GSE164690
Human CRC scRNAseq dataset (1)	Lee et al.^65^	GSE132465; GSE132257; GSE144735
Human CRC scRNAseq dataset (2)	Pelka et al.^66^	GSE178341
Human ESCC scRNAseq dataset	Zhang et al.^67^	GSE160269
Human NSCLC scRNAseq dataset (1)	Zilionis et al.^26^	GSE127465
Human NSCLC scRNAseq dataset (2)	Kim et al.^68^	GSE131907
Human prostate cancer scRNAseq dataset	Hirz et al.^69^	GSE181294
Human breast cancer scRNAseq dataset	Bassez et al.^46^	EGAS00001004809
Mouse lung cancer scRNAseq dataset (KP1.9)	Zilionis et al.^26^	GSE127465
Murine glioma scRNAseq dataset (IDH)	Alghamri et al.^95^	GSE152277
Murine melanoma scRNAseq dataset (B16F10)	Murgaski et al.^94^	GSE209763
Murine lung scRNAseq dataset (LLC)	Murgaski et al.^94^	GSE209763
Murine breast cancer scRNAseq dataset (MMTV-PyMT)	Ramos et al.^57^	GSE192935
Murine breast cancer scRNAseq dataset (MMTV-PyMT)	Li et al.^58^	GSE189856
Murine liver cancer scRNAseq dataset (*Nras*^G12D^/*Pten*^KO^)	Ramirez et al.^96^	GSE216717

Experimental models: Cell lines		

Murine MC38 colorectal carcinoma cell line	Mark J. Smyth	RRID: CVCL_B288
Murine MC38-H2B-Cerulean	Thorsten Mempel Lab	N/A
Murine D4M3.A-OVA melanoma cell line	Thorsten Mempel Lab	N/A
Murine B16F10 melanoma cell line	ATCC	CRL-6475; RRID: CVCL_0159

Experimental models: Organisms/strains		

Mouse: WT C57BL/6J	Charles River	Cat#000664
Mouse: B6.SJL-*Ptprc*^a^ *Pepc*^b^/BoyJ	Charles River	Cat#002014
Mouse: B6.129-*Il12b^tm1.1Lky^*/J	Jackson Laboratories	Cat#006412
Mouse: C57BL/6-*Foxp3^tm1Flv^*/ J	Jackson Laboratories	Cat#008374
Mouse: C57BL/6-*Ccr7^tm1.1Dnc^*/J	Jackson Laboratories	Cat#027913
Mouse: B6.129(Cg)-*Foxp3^tm3(HBEGF/GFP)Ayr^*/J	Jackson Laboratories	Cat#016958
Mouse: *Ccl22*-KO	David Anz Lab	Rapp et al.^93^
Mouse: B6(Cg)-*Zbtb46^tm1(HBEGF)Mnz^*/J	Jackson Laboratories	Cat#019506
Mouse: B6.129X1-*Ccl19^tm1Cys^*/J	Jackson Laboratories	Cat#012851
Mouse: *Ccl19*-tTA × LC1-Cre × R26-eYFP	Burkhard Ludewig Lab	Chen et al.^71^
Mouse: CNCr.129P2-*Cd40^tm1Kik^*/J	Jackson Laboratories	Cat#002927
Mouse: OT-IxCD45.1	Daniel Speiser Lab	N/A
Mouse: *Il-12p40*-IRES-eYFPx*FoxP3*-IRES-mRFP	AIVC	This study
Mouse: *Il-12p40*-IRES-eYFPx*FoxP3*-IRES-mRFPx*Ccl22*-KO	AIVC	This study

Software and algorithms		

FlowJo v10	FlowJo, LLC	RRID_008520
GraphPad Prism v10	GraphPad Prism	RRID_002798
QuPath v0.5.0 Digital Pathology	Bankhead et al.^101^	RRID_018257
IMARIS 10	Oxford Instruments	RRID_007370
METASCAPE	Zhou et al.^102^	RRID: SCR_016620
inForm 2.5.1 Automated Image Analyses	Akoya Bioscience	https://www.akoyabio.com/phenoimager/inform-tissue-finder/
R v4.5	R Core	https://www.r-project.org/
Code used for spatial analyses (R package *spati*)	This study	https://gitlab.com/pwirapati/spati
Code used for scRNAseq analyses (R package *scalpi*)	This study	https://www.gitlab.com/pwirapati/scalpi

## Highlights

- CCL19-CCR7 signaling clusters CCR7^+^ DCs around blood vessels for T cell activation
- Treg cells in these hubs suppress CD40 on CCR7^+^ DCs, restraining antitumor control
- Anti-PD-1 enhances CCR7^+^ DC clustering but increases CCL22-mediated Treg cell suppression
- CCR7^+^ DCs co-express both stimulatory and inhibitory programs
