# Supplementary material for: Positioning and reversible suppression of CCR7+ dendritic cells in perivascular tumor niches shape cancer immunity
Source: Immunity. Author manuscript; Available in PMC 2026 Feb 7. (PMC12882814; doi:10.1016/j.immuni.2025.11.020)
Supplement: Supplemental Figures [file NIHMS2134284-supplement-Supplemental_Figures.pdf]

Immunity, Volume *59*

Supplemental information

Positioning and reversible suppression of CCR7$^+$ dendritic cells in perivascular tumor niches shape cancer immunity

Beatrice Zitti, Florent Duval, Pratyaksha Wirapati, Mehdi Hicham, Yuxuan Xie, Juhyun Oh, Jan Hoelzl, Philippa Meiser, Marco Varrone, Hannah M. Peterson, Chiara Cianciaruso, Ruben Bill, Felix Bayerl, Evangelia Bolli, Anne-Gaëlle Goubet, Máté Kiss, Sheri McDowell, Phil Cheng, Dan Celestini, Julie Terzic, Thomas Zwahlen, Nagham Alouche, Nawel Zouggari, David Tarussio, Stephanie Tissot, Paula Nunes-Hasler, Mari Mino-Kenudson, Michael Lanuti, William C. Faquin, Peter M. Sadow, Jean-Christophe Tille, Sana Intidhar Labidi-Galy, Christopher S. Garris, Stephanie Hugues, Tatiana V. Petrova, Burkhard Ludewig, Sergio Quezada, Sanjiv Luther, Thorsten R. Mempel, Giovanni Ciriello, Sara I. Pai, Olivier Michielin, Jan P. Böttcher, Ralph Weissleder, and Mikael J. Pittet

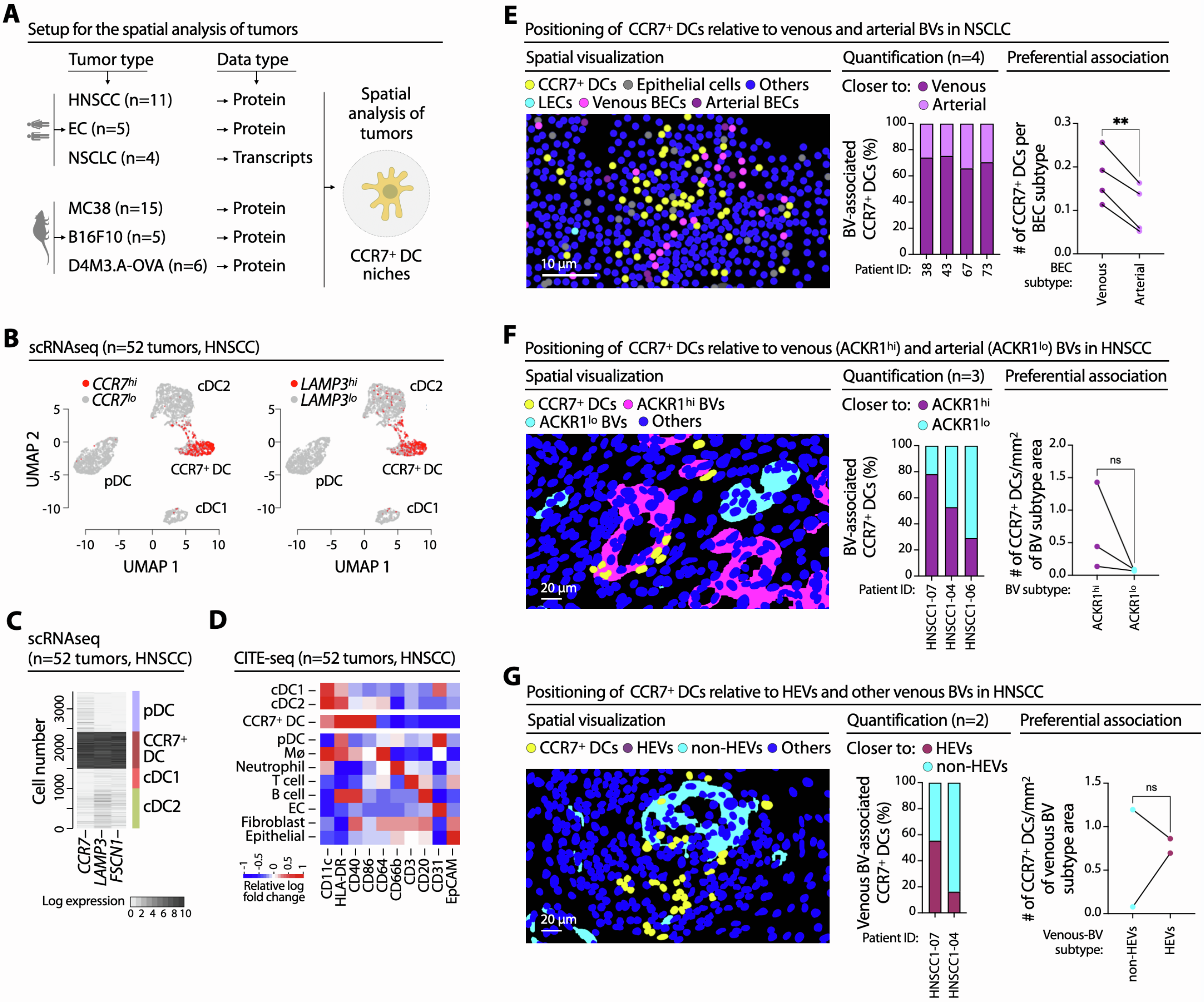

Figure S1: Analyses of BV- and LV-associated CCR7⁺ DCs in human tumors (related to main Figure 1)

A. Schematic outlining the tumor and the data types used for the spatial analysis of CCR7⁺ DCs in human (**Table S1**) and mouse tumors.

B. Uniform manifold approximation and projection (UMAP) visualization of tumor DCs from single-cell transcriptomic data combined from 52 HNSCC samples[S1],highlighting expression of *CCR7* (left UMAP) and *LAMP3* (right UMAP) in identified cell states.

C. Heatmap displays the expression of *CCR7*, *LAMP3* and *FSCN1* genes at single cell level in individual DCs in each state in HNSCC[S1].

D. Heatmap showing expression of protein markers as measured by CITEseq (rows), averaged over cells within states identified by corresponding scRNAseq profiles (columns) in HNSCC[S1].

E. Left: synthetic image of one representative FOV displaying CCR7⁺ DCs (yellow), venous BECs (light violet), arterial BECs (dark violet), LECs (cyan) and epithelial cells (grey) in one NSCLC patient[S2]. All cell subtypes were defined by their mRNA signature, as already annotated[S2]. Middle: frequencies of venous and arterial BEC-associated CCR7⁺ DCs among total BV-associated CCR7⁺ DCs. Whole tumor sections were analyzed. Scale bar represents 10 µm. One bar represents one NSCLC patient. Right: numbers of venous and arterial BEC-associated CCR7⁺ DCs normalized to the total number of venous and arterial BECs, respectively. Whole tumor sections were analyzed. One dot represents one patient; lines connect same patients. Paired t-test, **p<0.01.

F. Left: synthetic image of one representative FOV displaying CCR7⁺ DCs (yellow), venous BVs (identified as ACKR1^{hi}, magenta) and arterial BVs (identified as ACKR1^{lo}, cyan), in one HNSCC patient using

FAST cycling imaging. Scale bar represents 20 μm. Middle: frequencies of venous and arterial BV-associated $CCR7^+$ DCs among total BV-associated $CCR7^+$ DCs. Numbers of FOVs analyzed per sample: HNSCC1-04 n = 7; HNSCC1-06 n = 9; HNSCC1-07 n = 13. One bar represents one HNSCC patient. Right: numbers of venous and arterial BV-associated $CCR7^+$ DCs normalized to the total area of venous and arterial BVs, respectively. Numbers of FOVs analyzed per sample: HNSCC1-04 n = 7; HNSCC1-06 n = 9; HNSCC1-07 n = 13. One dot represents one patient; lines connect same patients.

G. Left: synthetic image of one representative FOV displaying $CCR7^+$ DCs (yellow), high endothelial venues (HEVs; identified as $ACKR1^{hi}MECA79^+$, purple) and non-HEV venous BVs (identified as $ACKR1^{hi}MECA79^–$, cyan) in one HNSCC patient using FAST cycling imaging. Scale bar represents 20 μm. Middle: frequencies of HEV and non-HEV venous BV-associated $CCR7^+$ DCs among total venous BV-associated $CCR7^+$ DCs. Numbers of FOVs analyzed per sample: HNSCC1-04 n = 1; HNSCC1-07 n = 10. One bar represents one HNSCC patient. Right: numbers of HEV and non-HEV venous BV-associated $CCR7^+$ DCs normalized to the total area of HEV and non-HEV venous BVs, respectively. Numbers of FOVs analyzed per sample: HNSCC1-04 n = 1; HNSCC1-07 n = 10. One dot represents one patient; lines connect same patients.

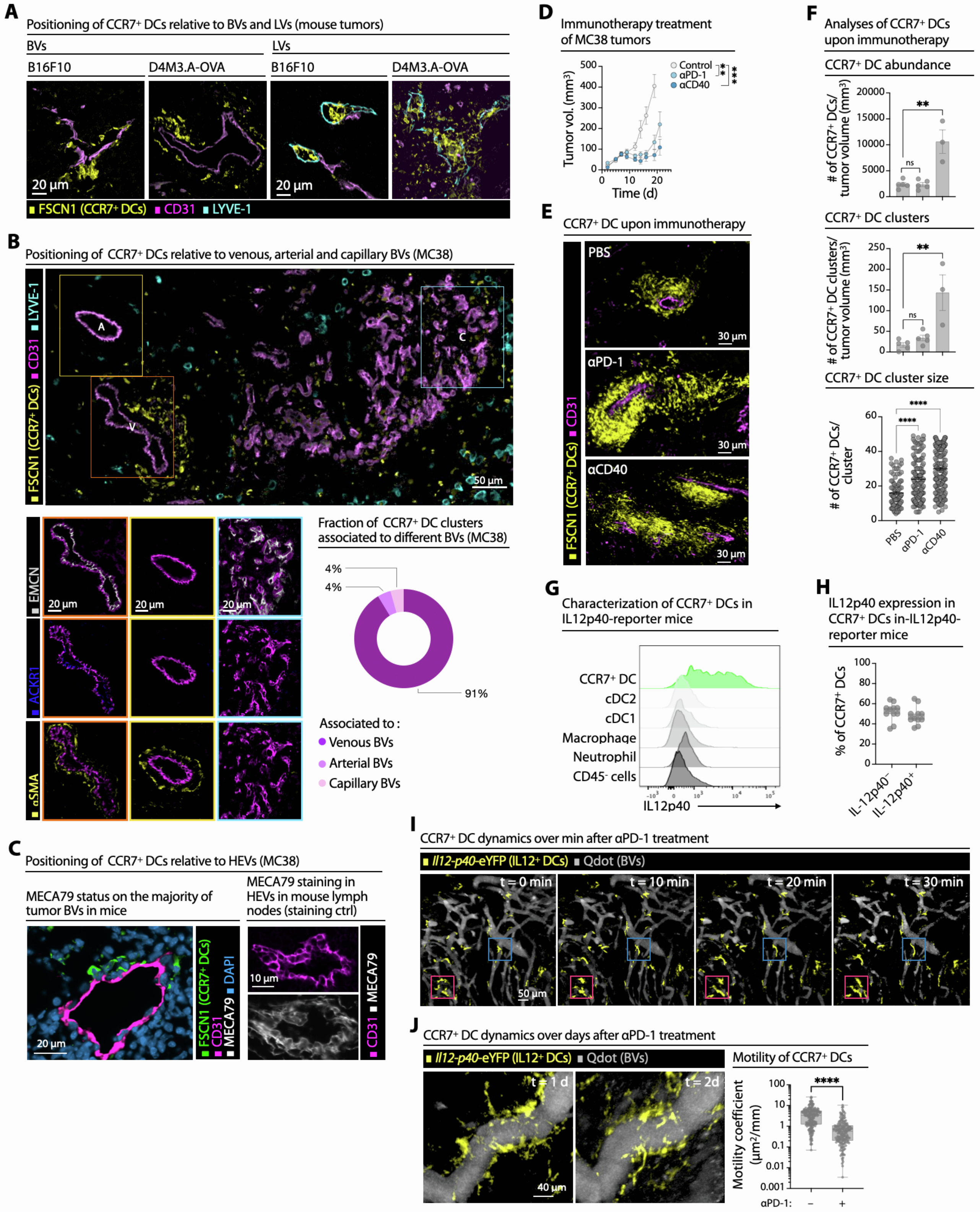

Figure S2: Analyses of BV- and LV-associated CCR7⁺ DCs in mouse tumors (related to main Figure 1)

A. Visualization of representative FOVs displaying CCR7⁺ DCs (identified as FSCN1⁺ cells; FSCN1 in yellow) located near BVs (identified as CD31⁺LYVE-1⁻ cells; CD31 in magenta) or LVs (identified as CD31⁺LYVE-1⁺ cells; LYVE-1 in cyan) in mouse B16F10 and D4M3.A-OVA tumors. Scale bar represents 20 µm.

B. Top: Representative FOV displaying CCR7^{+} DCs (identified as FSCN1^{+} cells; FSCN1 in yellow) located near BVs (identified as CD31^{+}LYVE-1^{-} cells; CD31 in magenta and LYVE-1 in cyan) in mouse MC38 tumors. Scale bar represents 50 µm. Squares highlight different examples of BV subtypes, namely arterial ("A"), venous ("V") and capillary ("C") BVs further characterized in lower left panels. Lower panels on the left: visualization of representative FOVs displaying markers used to identify different BV subtypes: endomucin (EMCN, white), ACKR1 (blue) and αSMA (yellow). Venous BVs are CD31^{+}EMCN^{+}ACKR1^{+}αSMA^{+} vessels, arterial BVs are CD31^{+}EMCN^{-}ACKR1^{-}αSMA^{+} vessels and capillary BVs are CD31^{+}EMCN^{+}ACKR1^{-}αSMA^{-} vessels. Scale bar represents 20 µm. Lower panel on the right: frequencies of perivascular CCR7^{+} DC clusters associated with venous, arterial or capillary BVs among total perivascular CCR7^{+} DC clusters (n=5 mice and 23 clusters). Whole tumor sections were analyzed.

C. Left: representative FOV displaying absence of HEVs (identified as CD31^{+}MECA79^{+} vessels) close to CCR7^{+} DCs (identified as FSCN1^{+} cells, green) in mouse MC38 tumors. Right: representative FOVs displaying staining of HEVs (identified as CD31^{+}MECA79^{+} vessels, CD31 in magenta and MECA79 in white) in MC38 tumor-draining lymph nodes (tdLNs) as positive control. Scale bar represents 20 µm.

D. Growth curves of MC38 tumors inoculated in WT mice left untreated or treated with anti-PD-1 or anti-CD40 immunotherapies. Tumor volume mean ± SEM per group (n=7-9 mice/group) is shown. Two-way ANOVA with multiple comparisons was used; **p<0.01, ***p<0.001 for comparisons at day 19.

E. Representative FOVs of volumetric immunofluorescence microscopy displaying CCR7^{+} DC clusters, marked by FSCN1 staining (yellow) located near vessels, marked by CD31 staining (magenta), in mouse MC38 tumors that were left untreated or three days after treatment with anti-PD-1 or anti-CD40 immunotherapies. Scale bar represents 30 µm.

F. Analyses of data acquired as in **E**. Top: Number of CCR7^{+} DCs normalized to tumor volumes, in mouse MC38 tumors that were left untreated or three days after treatment with anti-PD-1 or anti-CD40 immunotherapies (n=3-5 mice/group). Each dot represents one mouse. Unpaired t-test compared to untreated was used; mean ± SEM; **p<0.01. Middle: Numbers of CCR7^{+} DC clusters normalized to tumor volumes (n=3-5 mice/group). Each dot represents one mouse. Unpaired t-test compared to untreated was used; mean ± SEM; **p<0.01. Low: numbers of CCR7^{+} DCs per cluster (n=3-5 mice/group). Whole tumor sections were analyzed. Each dot represents one cluster. Unpaired t-test compared to untreated was used; mean ± SEM; ****p<0.0001.

G. Flow cytometry histogram showing expression of IL12p40 in different immune and non-immune populations from MC38 tumors.

H. Dot plot displays the percentage of IL12p40+ and IL12p40- CCR7+ DCs in MC38 tumors. Each dot represents a tumor. Whiskers represent min to max.

I. Representative FOVs displaying the migratory behavior of *Il12*-eYFP^{+} cells (yellow) in perivascular regions of MC38 tumors (vessels in grey), before and during treatment with anti-PD-1. Scale bar represents 50 µm. Red and blue squares highlight examples of *Il12*-eYFP^{+} cells that persist at BVs during treatment.

J. Left: representative FOVs displaying the migratory behavior of *Il12*-eYFP^{+} cells clusters (yellow) in perivascular regions of MC38 tumors (vessels in grey) one and two days after treatment with anti-PD-1. Scale bar represents 40 µm. Right: motility coefficient displays the motility of *Il12*-eYFP^{+} cells over time before and 24 hours after treatment with anti-PD-1 (n=4). One FOV per mouse was analyzed. Each dot represents one *Il12*-eYFP^{+} cells. Unpaired t-test, whiskers represent min to max; ****p < 0.0001.

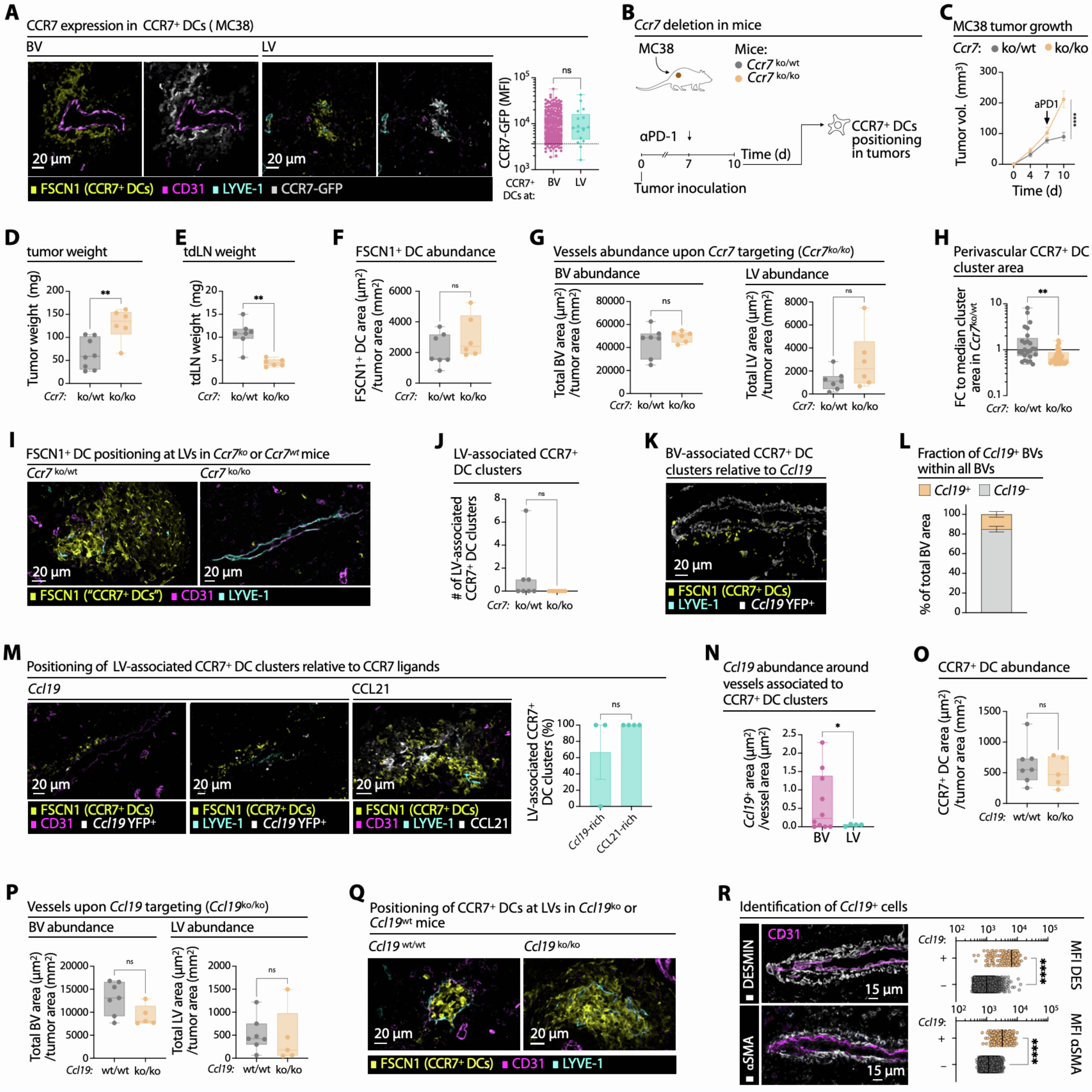

Figure S3: Analyses of BV- and LV-associated CCR7⁺ DCs upon perturbation of the CCR7-CCL19 axes (related to main Figure 1)

A. Left: representative FOVs displaying GFP expression (white) reporting for *Ccr7* in FSCN1⁺ DCs (yellow) associated with BVs (identified as CD31⁺LYVE-1⁻, CD31 in magenta) or LVs (identified as CD31⁺LYVE-1⁺, LYVE-1 in cyan) in MC38 tumors inoculated in *Ccr7*-GFP (*Ccr7*$^{gfp/wt}$) mice. Scale bar represents 20 µm. Right: plot displays expression levels of CCR7-GFP (mean fluorescence intensity, MFI) in FSCN1⁺ DCs associated with BVs or LVs (n=2). Each dot represents one surface. Unpaired t-test. Whiskers represent min to max.

B. Scheme outlining the experimental setup for analysis of the spatial positioning of CCR7+ DCs in MC38 tumors inoculated in anti-PD-1 treated *Ccr7*$^{ko/wt}$ and *Ccr7*$^{ko/ko}$ mice.

C. Growth curves of MC38 tumors inoculated in anti-PD-1 treated *Ccr7*$^{ko/wt}$ and *Ccr7*$^{ko/ko}$ mice. Tumor volume mean ± SEM per group (*Ccr7*$^{ko/ko}$ n=5 mice and *Ccr7*$^{wt/ko}$ n=6 mice) is shown. Two-way ANOVA with multiple comparisons was used; ****p<0.0001 for comparisons at day 10.

D. Weight of MC38 tumors inoculated in *Ccr7*$^{ko/wt}$ and *Ccr7*$^{ko/ko}$ mice harvested 3 days post anti-PD-1 treatment. Each dot represents one mouse. Unpaired t-test, whiskers represent min to max; **p<0.01.

E. tdLN weight from MC38 tumor-bearing mice (*Ccr7*$^{ko/wt}$ and *Ccr7*$^{ko/ko}$) harvested 3 days post anti-PD-1 treatment. Each dot represents one mouse. Unpaired t-test, whiskers represent min to max; **p<0.01.

F. Total FSCN1$^+$ DC ("CCR7$^+$ DC") areas normalized to tumor areas in MC38 tumors inoculated in *Ccr7*$^{ko/wt}$ and *Ccr7*$^{ko/ko}$ mice and analyzed three days after anti-PD-1 immunotherapy (n=6-7). Whole tumor sections were analyzed. Each dot represents one mouse. Unpaired t-test. Whiskers represent min to max.

G. Total BV (identified as CD31$^+$LYVE-1$^–$ vessels) or LV (identified as CD31$^+$LYVE-1$^+$ vessels) areas normalized to tumor areas in MC38 tumors inoculated in *Ccr7*$^{ko/wt}$ and *Ccr7*$^{ko/ko}$ mice and analyzed three days after anti-PD-1 immunotherapy (n=6-7). Whole tumor sections were analyzed. Each dot represents one mouse. Unpaired t-test. Whiskers represent min to max.

H. Change in BV-associated FSCN1$^+$ DC ("CCR7$^+$ DC") cluster area comparing *Ccr7*$^{ko/ko}$ and *Ccr7*$^{ko/wt}$ mice. Each dot represents one FSCN1$^+$ DC cluster (*Ccr7*$^{ko/ko}$ n=5 mice and 30 clusters, *Ccr7*$^{wt/ko}$ n=6 mice and 25 clusters). Whole tumor sections were analyzed. Each dot represents a BV associated with a cluster of CCR7$^+$ DCs. Unpaired t-test, whiskers represent min to max; **p < 0.01.

I. Visualization of representative FOVs displaying FSCN1$^+$ DCs ("CCR7$^+$ DC"; FSCN1 in yellow) located near LVs (identified as CD31$^+$LYVE-1$^+$ cells; CD31 in magenta and LYVE-1 in cyan) in MC38 tumors inoculated in *Ccr7*$^{ko/wt}$ and *Ccr7*$^{ko/ko}$ mice and analyzed three days after anti-PD-1 immunotherapy. Scale bar represents 20 μm.

J. Number of LV-associated FSCN1$^+$ DC clusters in MC38 tumors inoculated in *Ccr7*$^{ko/wt}$ and *Ccr7*$^{ko/ko}$ mice and analyzed three days after anti-PD-1 immunotherapy. Whole tumor sections were analyzed. Each dot represents one mouse. Unpaired t-test. Whiskers represent min to max.

K. Visualization of a FOV displaying absence of LYVE-1 (cyan) staining in the *Ccl19*-covered vessel associated with the CCR7$^+$ DC cluster shown in **Figure 1E** (consecutive section to the one represented in **Figure 1E**, left image). Scale bar represents 20 μm.

L. Frequencies of *Ccl19*-covered and -uncovered BV areas among total BV area in MC38 tumors inoculated in *Ccl19*-ieYFP mice and analyzed three days after anti-PD-1 immunotherapy. Whole tumor sections were analyzed. Mean ± SEM is shown.

M. Left: visualization of representative FOVs displaying CCR7$^+$ DCs (identified as FSCN1$^+$ cells; FSCN1 in yellow) located near LVs (identified as CD31$^+$LYVE-1$^+$ cells; CD31 in magenta and LYVE-1 in cyan) and *Ccl19* (identified as *Ccl19*-eYFP$^+$ Tomato$^+$ cells using a *Ccl19*-ieYFP reporter mouse, white, left images show two consecutive sections) or CCL21 (white, right image) in mouse MC38 tumors. Scale bar represents 20 μm. Right: frequencies of LV-associated CCR7$^+$ DC clusters associated with *Ccl19*-covered LVs or within CCL21$^+$ areas of the tumors among total LV-associated CCR7$^+$ DC clusters. Whole tumor sections were analyzed. Each dot represents one mouse. Unpaired t-test. Mean ± SEM is shown.

N. *Ccl19*$^+$ area within a 20 μm radius of associated BVs or LVs, normalized to the area of the respective BVs or LVs in MC38 tumors in *Ccl19*-ieYFP reporter mice. Whole tumor sections were analyzed. Each dot represents a vessel associated with a cluster of CCR7$^+$ DCs. Unpaired t-test, whiskers represent min to max; *p<0.05.

O. Total CCR7$^+$ DC areas normalized to tumor areas in MC38 tumors inoculated in littermate *Ccl19*$^{wt/wt}$ and *Ccl19*$^{ko/ko}$ mice and analyzed two days after anti-PD-1 immunotherapy (n=5-7). Whole tumor sections were analyzed. Each dot represents one mouse. Unpaired t-test. Whiskers represent min to max.

P. Total BV (identified as CD31$^+$LYVE-1$^–$ vessels) or LV (identified as CD31$^+$LYVE-1$^+$ vessels) areas normalized to tumor areas in MC38 tumors inoculated in littermate *Ccl19*$^{wt/wt}$ and *Ccl19*$^{ko/ko}$ mice and analyzed two days after anti-PD-1 immunotherapy (n=5-7). Whole tumor sections were analyzed. Each dot represents one mouse. Unpaired t-test. Whiskers represent min to max.

Q. Visualization of representative FOVs displaying CCR7$^+$ DCs (identified as FSCN1$^+$ cells; FSCN1 in yellow) located near LVs (identified as CD31$^+$LYVE-1$^+$ cells; CD31 in magenta and LYVE-1 in cyan) in mouse MC38 tumors inoculated in littermate *Ccl19*$^{wt/wt}$ and *Cccl19*$^{ko/ko}$ mice and analyzed two days after anti-PD-1 immunotherapy. Scale bar represents 20 μm.

R. Left: Visualization of representative FOVs displaying fibroblast markers (desmin, top and αSMA, bottom) expressed by *Ccl19*-expressing cells covering the same BV associated with a $CCR7^{+}$ DC cluster shown in **Figures 1E** and **S3G** (consecutive sections to the one represented in **Figure 1E**, left image). Scale bar represents 15 μm. Right: MFI of desmin and αSMA in *Ccl19*-YFP^{+} Tomato+ ($Ccl19^{+}$) and *Ccl19*-YFP^{-} $Tomato^{-}$ ($Ccl19^{-}$) surfaces from MC38 tumors inoculated in *Ccl19*-ieYFP reporter mice and analyzed three days after anti-PD-1 immunotherapy. Each dot represents one surface (n=3 mice). Unpaired t-test, median is shown; ****$p<0.0001$.

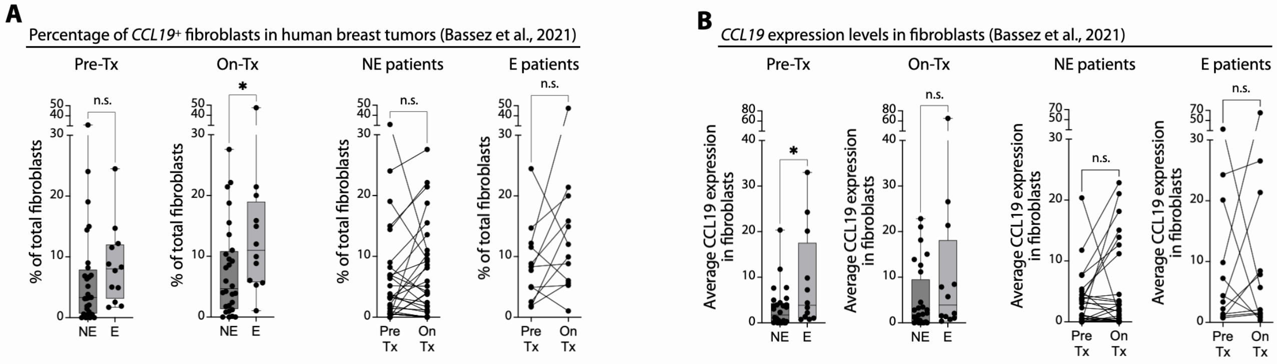

Figure S4: Quantification of CCL19-expressing fibroblasts upon immunotherapy in human tumors (related to main Figure 1).

A. Percentage of *CCL19*+ fibroblasts in breast cancer patients receiving anti-PD-1 therapy[S3]. Patients without (NE) or with (E) T cell clonotype expansion during treatment (Pre- vs On-Tx) are distinguished. From left to right, the data compare i) NE and E patients Pre-Tx (unpaired t-test), ii) NE and E patients On-Tx (unpaired t-test; *p<0.05), iii) NE patients Pre- vs On-Tx (paired t-test), iv) E patients Pre- vs On-Tx (paired t-test).

B. Same as **A.**, but showing relative *CCL19* expression levels in fibroblasts.

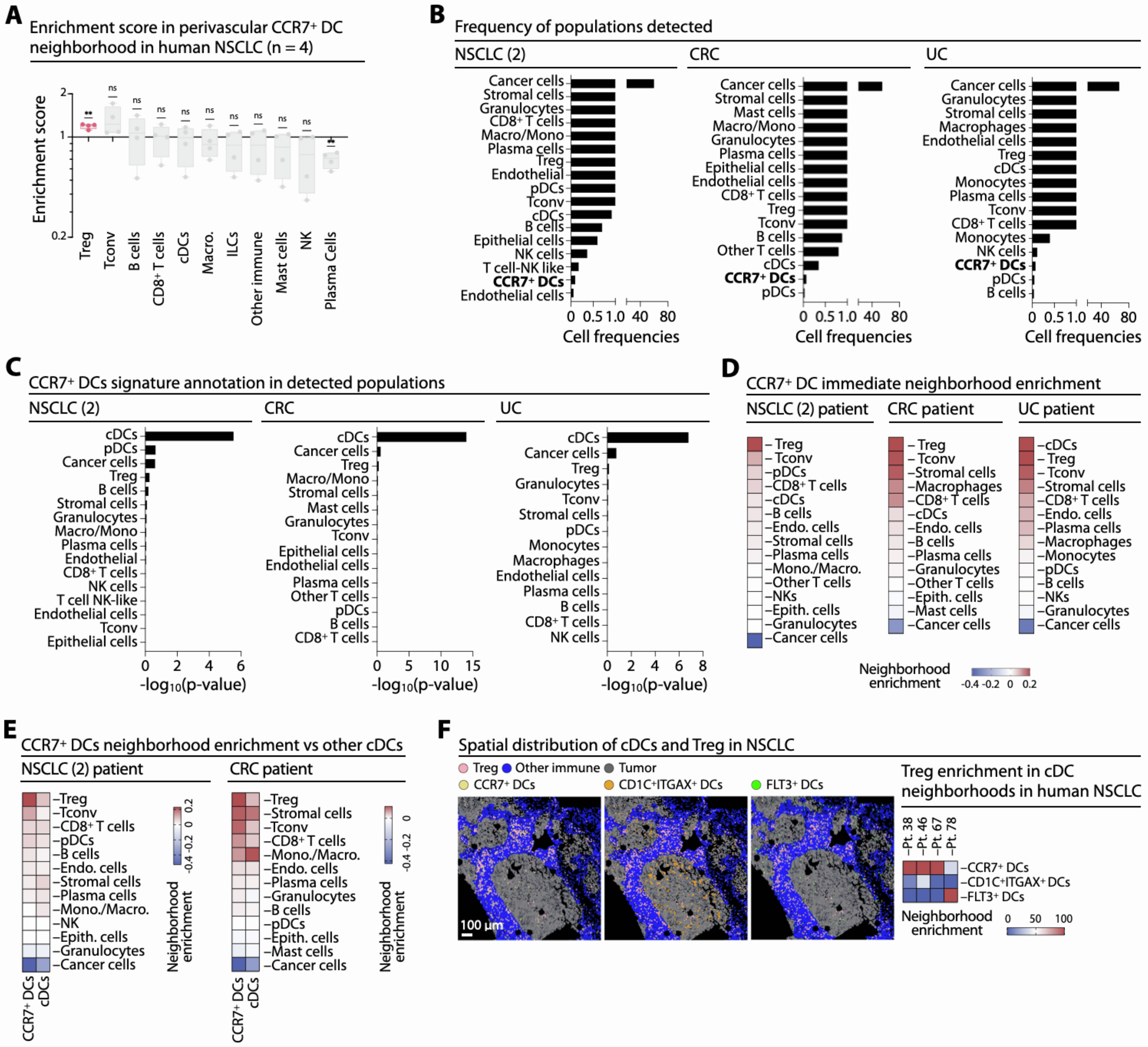

Figure S5: Analyses of cellular neighbours of perivascular CCR7⁺ DCs (related to main Figure 2).

A. Box plots depict the enrichment score of indicated immune cell populations in the perivascular CCR7⁺ DC neighborhood in four NSCLC samples[S2]. Each dot represents one patient. Median and IQR within each population is displayed. One sample t-test with n=4 testing for the ratio > 1, ***p<0.001, **p<0.01 and *p<0.05.

B. Bar plots depict frequencies of annotated cell states in each dataset described in spatial transcriptomic data of tumor tissues from one NSCLC, one CRC and one uterine cancer (UC) patient[S4].

C. Bar plots depict -$\log_{10}$ raw binomial p-values of CCR7⁺ DC signature genes annotation in populations detected in each dataset described in **B**.

D. Neighborhood enrichment analyses performed on spatial transcriptomic data (**B**). Heatmaps display the enrichment of identified immune and non-immune cell types in CCR7⁺ DC neighborhoods in each tumor.

E. Heatmaps display the enrichment of identified immune and non-immune cell types in CCR7⁺ DC compared to other cDCs neighborhoods in NSCLC and CRC tumors.

F. Left: synthetic images of one representative FOV displaying CCR7⁺ DCs (yellow), CD1C⁺ITGAX⁺ DCs (orange), FLT3⁺ DCs (green) and Treg (pink) in one representative NSCLC patient analyzed by spatial

transcriptomics[S2]. Whole tumor sections were analyzed. Scale bar represents 100 μm. Right: heatmap displays normalised Treg enrichment near $CCR7^+$ DC, $CD1C^+ITGAX^+$ DC or $FLT3^+$ DC neighborhoods in four NSCLC patients.

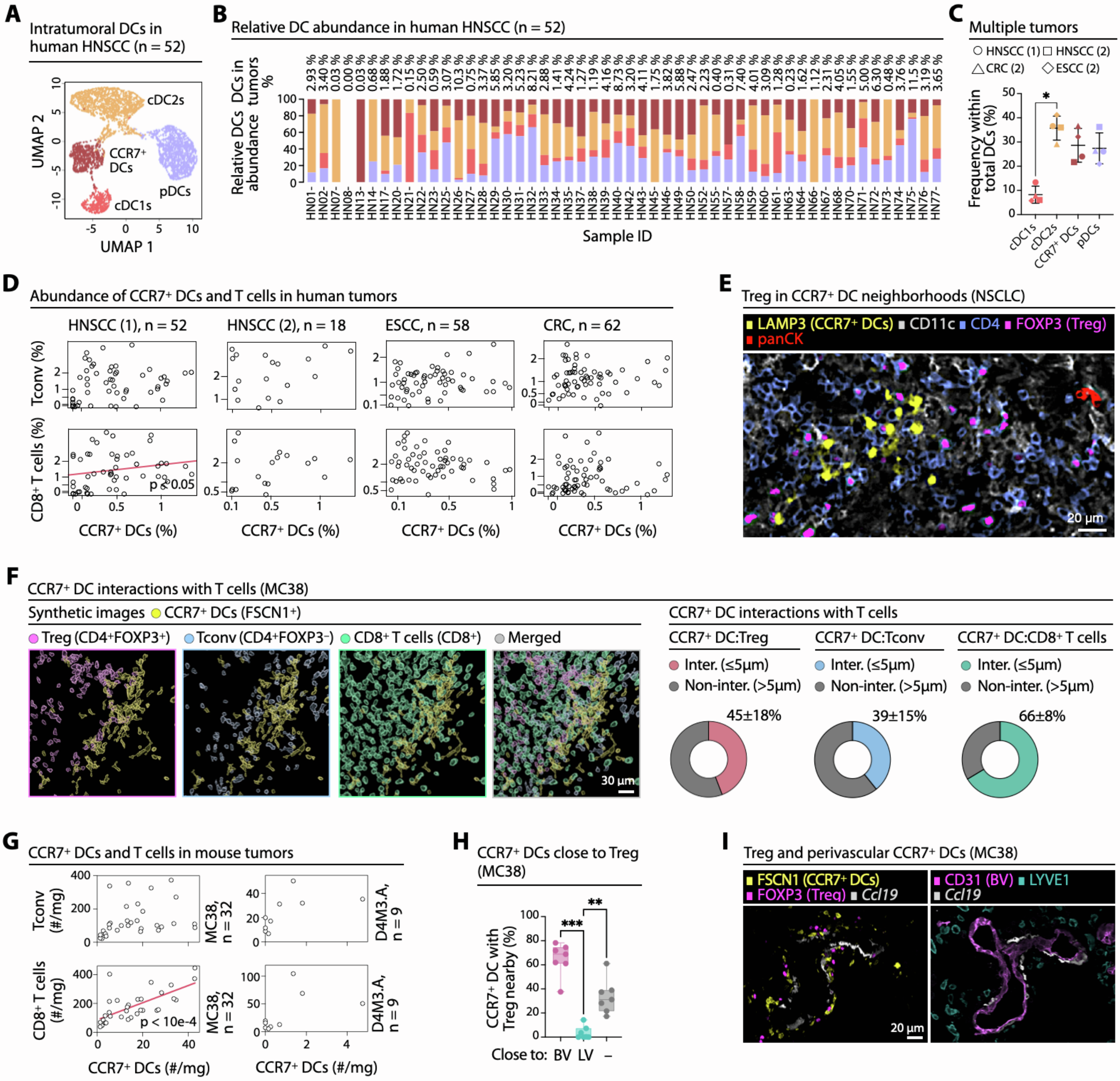

Figure S6: Analyses of CCR7⁺ DCs and T cell states in human and mouse tumors (related to main Figure 2).

A. Uniform manifold approximation and projection (UMAP) visualisation of tumor DCs from single-cell transcriptomic data combined from all 52 samples from the HNSCC cohort[S1], highlighting the separation of DC states.

B. Relative abundance of DC states in individual tumor samples from the HNSCC cohort (**A**), numbers represent the percentages of total DCs among all cells sequenced.

C. Plot depicts the average frequencies of indicated DC states among all intratumoral DCs in tumors. Shapes indicate the datasets and tumor types analyzed (HNSCC (1)[S1], HNSCC (2)[S5], CRC[S6], ESCC[S7]). Friedman test was used, mean with SEM; *p<0.05.

D. Correlations between the proportions of CCR7⁺ DCs and T_{CONV} or CD8⁺ T cells within CD45⁺ cells, as determined by scRNAseq in multiple human cancer types. Spearman rank correlation; significant correlations are shown with a fitted red line.

E. Representative FOVs depicting CCR7⁺ DCs (identified as CD11c⁺ and LAMP3⁺, yellow) and Treg (identified as CD4⁺ and FOXP3⁺, magenta) in their proximity in NSCLC using multispectral imaging. Scale bar represents 20 µm.

F. Left: representative FOVs of volumetric immunofluorescence microscopy displaying CCR7$^+$ DCs, marked by FSCN1 staining (surfaced in yellow), Treg, marked by FOXP3 staining (surfaced in magenta), T_{CONV}, marked by CD4 and lack of FOXP3 staining (surfaced in turquoise) and CD8$^+$ T cells, marked by CD8 staining (surfaced in green) in MC38 tumors. Scale bar represents 30 µm. Right: frequencies of CCR7$^+$ DCs interacting (<5 µm) with indicated T cell subsets (n=5). One-way ANOVA with multiple comparisons was used; *$p<0.05$ and **$p<0.01$. Whole tumor sections were analyzed.

G. Correlations between the numbers of CCR7$^+$ DCs and T_{CONV} or CD8$^+$ T cells per mg of tumor tissue, as determined by flow cytometry analyses of MC38 and D4M3.A mouse tumors. Spearman rank correlation; significant correlations are shown with a fitted red line.

H. Box plots display the frequencies of BV-associated, LV-associated and non vessel-associated CCR7$^+$ DCs close (< 5 µm) to Treg among all tumor CCR7$^+$ DCs with nearby Treg. One-way ANOVA test, whiskers represent min to max; ***$p<0.001$ and **$p<0.01$.

I. Representative FOVs of confocal microscopy showing two consecutive sections. Left: CCR7$^+$ DCs (identified as FSCN1$^+$, yellow), Treg (identified as FOXP3$^+$, magenta) and *Ccl19*-expressing cells (identified as *Ccl19*-eYFP$^+$ Tomato$^+$ cells using a *Ccl19*-ieYFP reporter mouse, white). Right: BVs (identified as CD31$^+$ and Lyve1$^-$, magenta), LVs (identified as CD31$^+$ and Lyve1$^+$, cyan) and Ccl19-expressing cells (identified as *Ccl19*-eYFP$^+$ Tomato$^+$ cells using a *Ccl19*-ieYFP reporter mouse, white). Scale bar represents 20 µm.

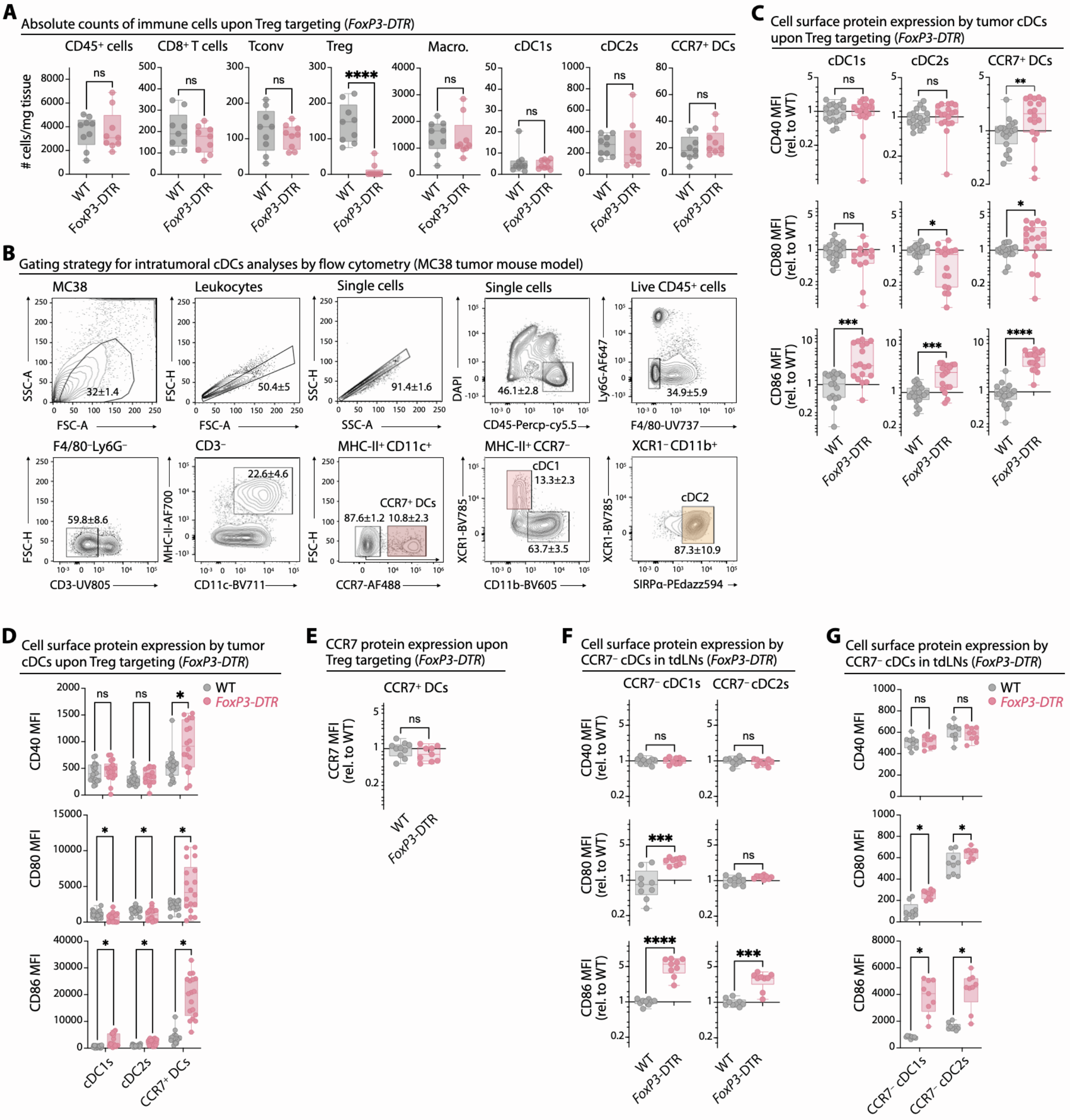

Figure S7: Analyses of CCR7+ DCs upon genetic perturbation of Treg cells (related to main Figure 3).

A. Absolute counts of indicated cell subsets in MC38 tumors from WT or Treg-depleted mice measured by flow cytometry (n=9). Each dot represents one mouse, whiskers represent min to max. Unpaired t-test was used; ****p<0.0001.

B. Gating strategy for the analyses of CCR7⁺ DCs, cDC1s and cDC2s by flow cytometry and FACS sorting of CCR7+ DCs from mouse MC38 tumors.

C. MFI of CD40, CD80 and CD86 protein expression measured by flow cytometry in cDC1s, cDC2s and CCR7⁺ DCs in MC38 tumors from WT or Treg-depleted mice (n = 18) and expressed as normalised values. Each dot represents one mouse, whiskers represent min to max. Unpaired t-test was used; ***p<0.001, **p<0.01 and *p<0.05.

D. MFI of CD40, CD80 and CD86 as in C and expressed as absolute MFI values. Each dot represents one mouse, whiskers represent min to max. Multiple unpaired t-test was used; adjusted p-values are shown, *p<0.05.

E. MFI of CCR7 protein expression measured by flow cytometry in $CCR7^+$ DCs in MC38 tumors from WT or Treg-depleted mice (n=18). Each dot represents one mouse, whiskers represent min to max. Unpaired t-test was used.

F. MFI of CD40, CD80 and CD86 protein expression measured by flow cytometry in $CCR7^-$ DCs in tumor-draining lymph nodes (tdLNs) from MC38 tumors from WT or Treg-depleted mice (n = 9) and expressed as normalised values. Each dot represents one mouse, whiskers represent min to max. Unpaired t-test was used; **p<0.01 and *p<0.05.

G. MFI of CD40, CD80 and CD86 as in F and expressed as absolute MFI values. Each dot represents one mouse, whiskers represent min to max. Multiple unpaired t-test was used; adjusted p-values are shown, *p<0.05.

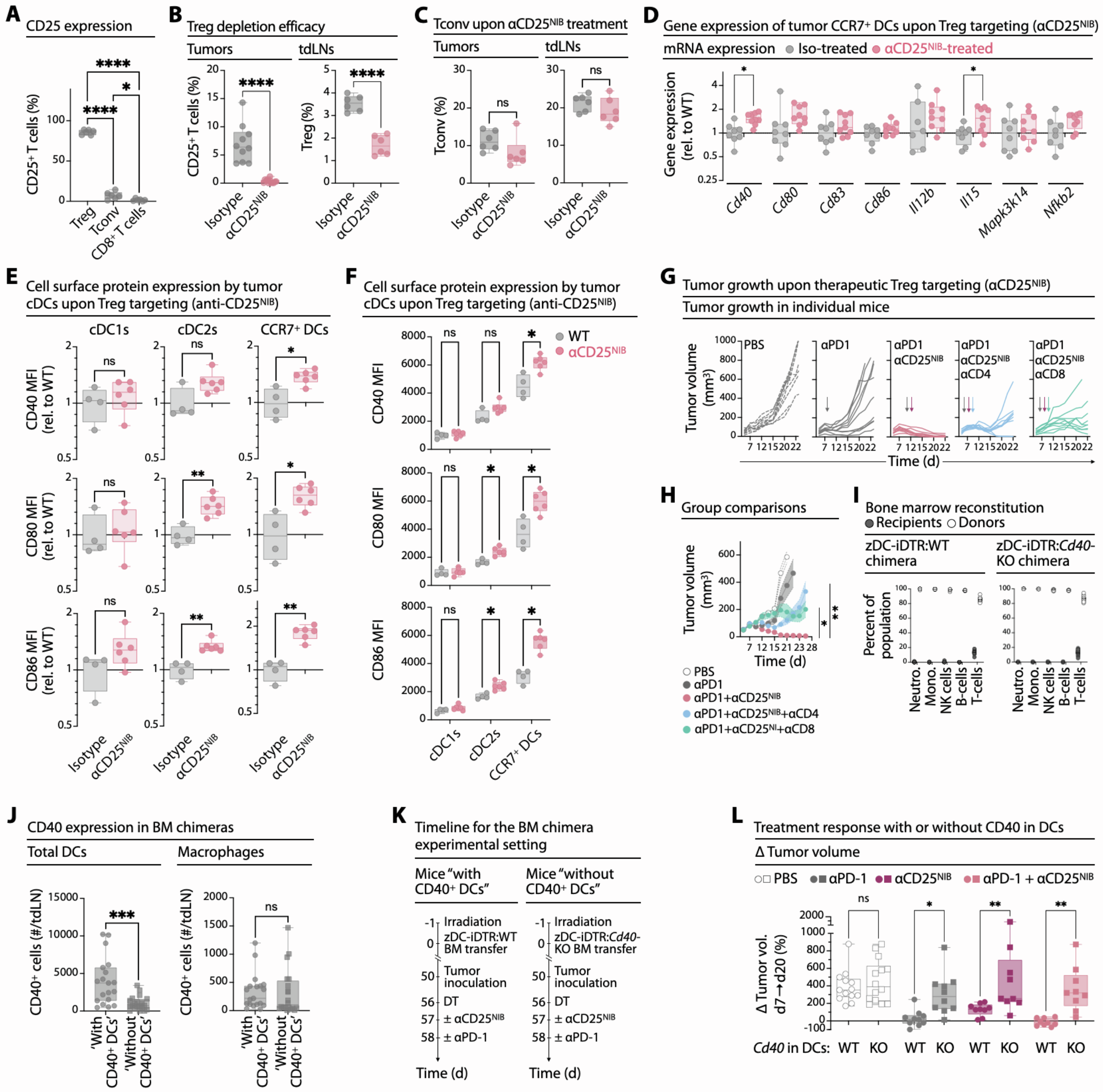

Figure S8: Analyses of CCR7⁺ DCs upon therapeutic depletion of Treg cells with anti-CD25NIB and contribution of CD40 expression by DCs (related to main Figure 3).

A. Frequencies of CD25-expressing T cells in MC38 tumors (n=6). Each dot represents one mouse, whiskers represent min to max. One-way ANOVA with multiple comparisons was used; *p<0.05, ****p<0.0001.

B. Efficacy of Treg depletion with αCD25NIB antibodies was assessed by analyzing the frequencies of CD25-expressing T cells in MC38 tumors (left) of FOXP3-expressing T cells in tdLNs (right; n=10 or 6). Each dot represents one mouse, whiskers represent min to max. Unpaired t-test was used; ****p<0.0001.

C. Frequencies of T_{CONV} cells in both tumors and tdLNs upon treatment with αCD25NIB (n=6). Each dot represents one mouse, whiskers represent min to max.

D. Relative expression levels of candidate immunostimulatory genes expressed by tumor CCR7⁺ DCs isolated from WT mice treated with anti-PD-1+isotype control (grey) or anti-PD-1+αCD25NIB antibodies

(pink) and analyzed by bulk RNAseq (n=9). Each dot represents one mouse, whiskers represent min to max. Unpaired t-test with multiple comparisons was used; *p<0.05.

E. MFI of CD40, CD80 and CD86 protein expression measured by flow cytometry in cDC1s, cDC2s and CCR7$^+$ DCs in MC38 tumors from isotype- or anti-CD25^{NIB}-treated mice (n = 4 and n = 6) and expressed as normalized values. Each dot represents one mouse, whiskers represent min to max. Unpaired t-test was used; **p<0.01 and *p<0.05.

F. MFI of CD40, CD80 and CD86 as in E and expressed as absolute MFI values. Each dot represents one mouse, whiskers represent min to max. Multiple unpaired t-test was used; adjusted p-values are shown, *p<0.05.

G. Growth curves of MC38 tumors inoculated in WT mice left untreated or treated with anti-PD-1 alone, anti-PD-1 + αCD25^{NIB} combination and CD4 or CD8 depletion in anti-PD-1+ αCD25^{NIB} combination. Tumor volumes in individual mice is shown.

H. Growth curves of MC38 tumors as in G. Mean ± SEM (n=8 or 9 mice/group). Ordinary two-way ANOVA with multiple comparisons was used; *p<0.05, **p<0.01.

I. Frequencies of donor-derived immune cells (CD45.2) in peripheral blood of reconstituted recipient mice (CD45.1) in both zDC-iDTR:WT and zDC-iDTR:Cd40-KO chimeras (n=20). Each dot represents one mouse.

J. Absolute numbers of CD40-expressing total cDCs (left) or macrophages (right) in tdLNs of zDC-iDTR:WT and zDC-iDTR:Cd40-KO chimeras (n=20). Each dot represents one mouse, whiskers represent min to max. Unpaired t-test was used; ***p<0.001.

K. Scheme outlining the timeline for the generation of bone marrow chimeras with inducible *Cd40*-deficiency in cDCs and treatments schedule.

L. Change of tumor volume in zDC-iDTR:WT and zDC-iDTR:Cd40-KO bone marrow chimeras left untreated, or in response to anti-PD-1, αCD25^{NIB} or a combination of anti-PD-1 and αCD25^{NIB} (n=13 or 9). Each dot represents one mouse, whiskers represent min to max. Two-way ANOVA with multiple comparisons was used; *p<0.05, **p<0.01.

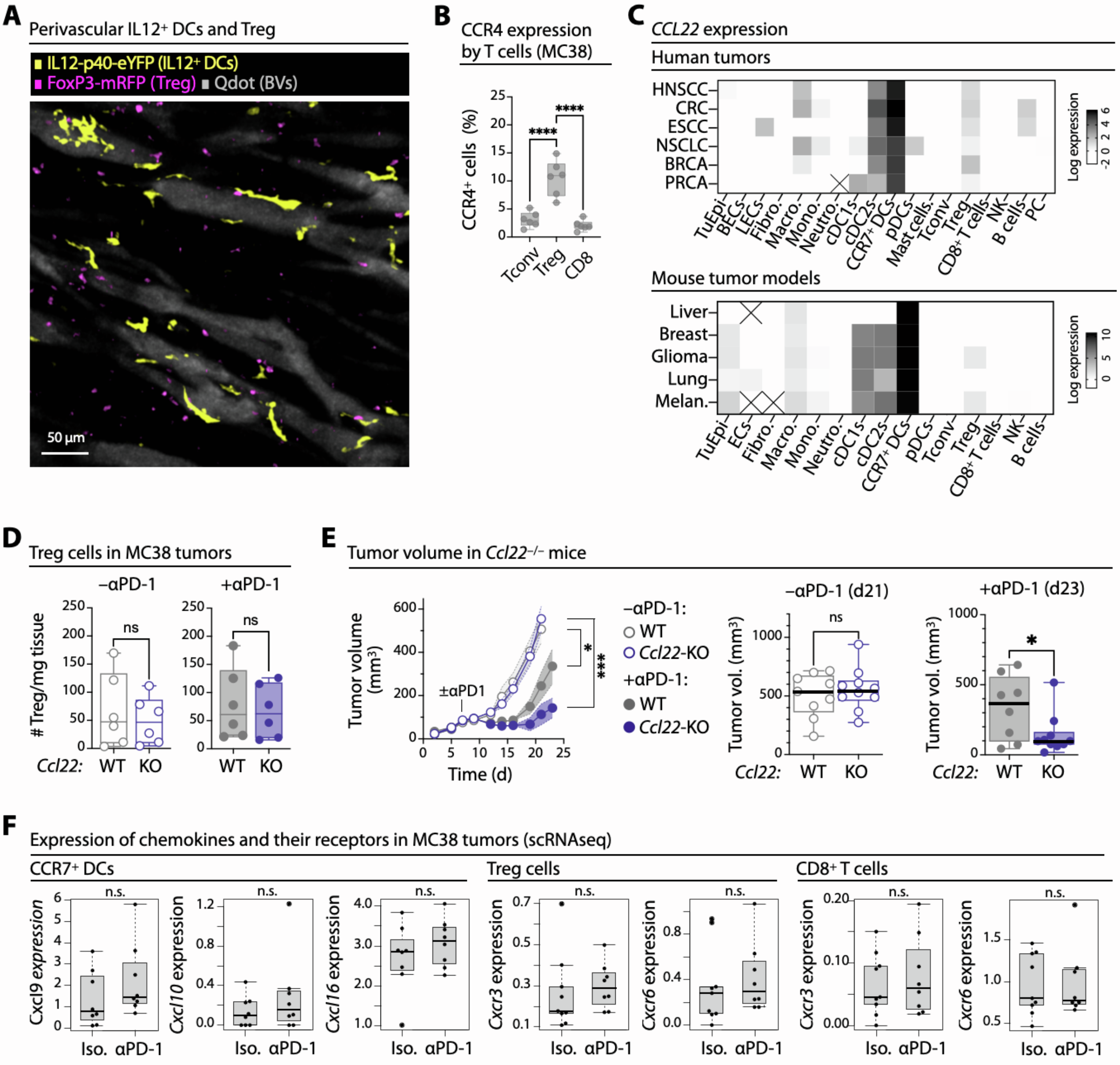

Figure S9: Analyses of *Ccl22* and *Ccl22*-KO mice upon immunotherapy (related to main Figure 4).

A. Representative FOVs displaying Treg (*FoxP3*-mRFP, magenta) interacting with *Il12*-eYFP+ DCs (yellow) in perivascular regions (vessels in grey) of tumors. Scale bar represents 50 µm.

B. Box plots depict the frequencies of CCR4-expressing cells among the indicated T cell subsets in MC38 tumors (n = 6). Two-way ANOVA with multiple comparisons was used, whiskers represent min to max; ****p<0.0001.

C. Heatmaps depicts $\log_2$ transformed averaged expression of *CCL22* in indicated immune and non-immune populations in the TME of multiple human tumors (left) or mouse tumor models (right). A cross indicates that the cellular population was not detected.

D. Absolute counts of Treg cells in MC38 tumors from WT or *Ccl22*-KO mice, either untreated (left) or treated with anti-PD-1 (right), measured by flow cytometry (n = 6). Each dot represents one mouse, whiskers represent min to max. Unpaired t-test was used.

E. Left: growth curves of MC38 tumors inoculated in WT or *Ccl22*-KO mice left untreated or in response to anti-PD-1 treatment. Graph displays mean ± SEM of tumor volumes (n=10 mice/group). Ordinary two-way ANOVA with multiple comparisons was used, *p<0.05, **p<0.01. Right: tumor volume calculated at day 21 (left) or day 23 (right) in WT and *Ccl22*-KO mice left untreated (left) or upon anti-PD-1

immunotherapy (right). Each dot represents one mouse, whiskers represent min to max. Unpaired t-test was used; *$p<0.05$.

F. Box plots depict averaged expression of *Cxcl9*, *Cxcl10* and *Cxcl16* in $CCR7^+$ DCs and *Cxcr3* and *Cxcr6* in Treg and $CD8^+$ T cells in the TME of MC38 tumors three days after immunotherapy with anti-PD-1 or isotype control. Whiskers represent min to max.

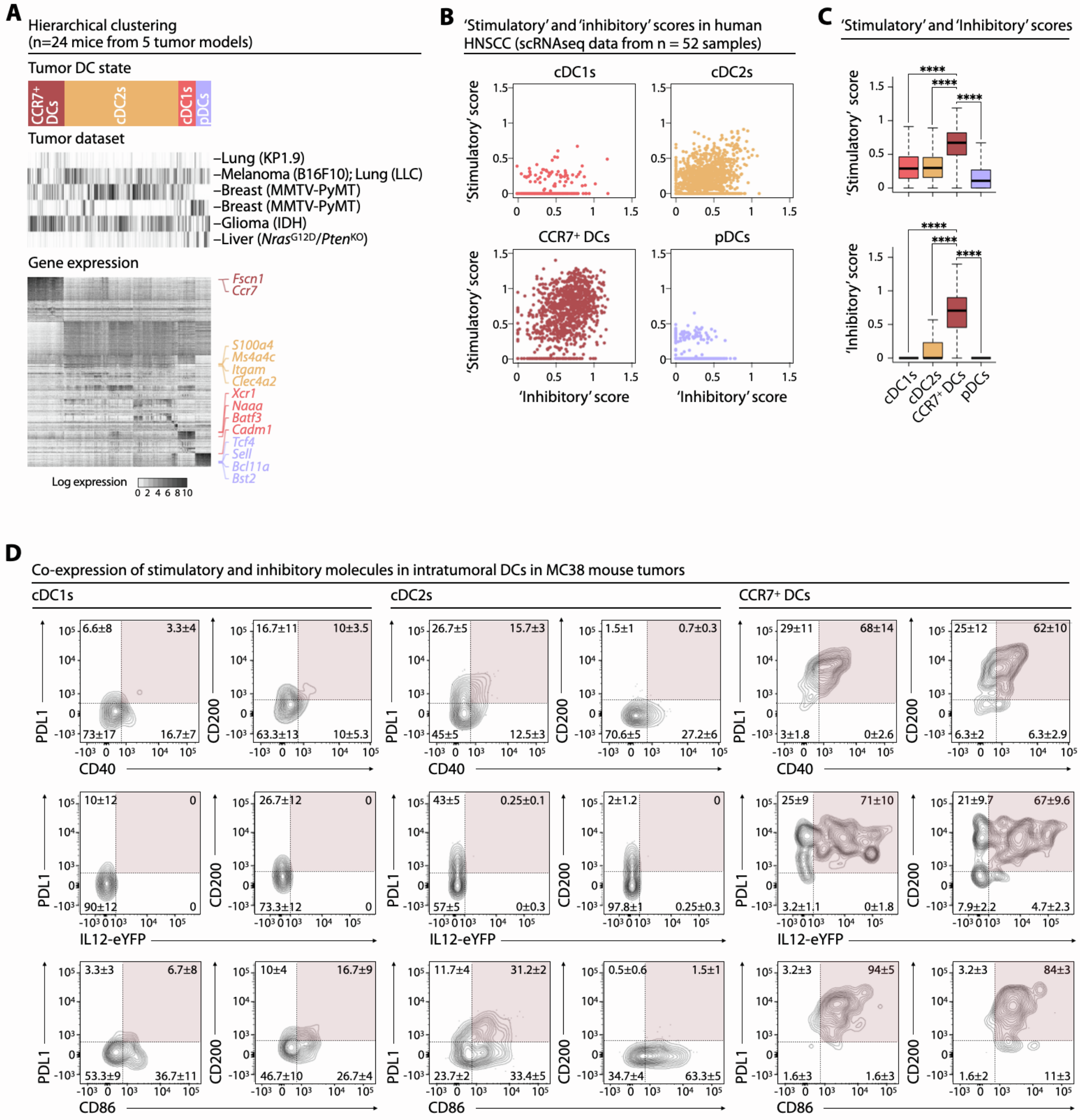

Figure S10: Analyses of CCR7⁺ DC stimulatory and suppressive phenotypes in human and mouse tumors (related to main Figure 5).

A. Unsupervised clustering of total DCs based on scRNAseq data from 24 mice and 5 experimental tumor models[S8-13]. Top 10 expressed genes in manually annotated DC states are highlighted on the right.

B. Dot plots display 'stimulatory' and 'inhibitory' scores calculated for individual cells in each intratumoral DC state. Score were generated for each cell based on average expression of respective gene sets.

C. Box plots display 'stimulatory' and 'inhibitory' scores calculated as the sum of the average expression of individual 'stimulatory' and 'inhibitory' marker genes expressed in single DCs in each state. T-test with multiple comparisons was used; ****p<0.0001.

D. Flow cytometry plots display co-expression of indicated 'stimulatory' (CD40, IL12, CD86) and inhibitory (PDL1, CCL22) markers in cDC1s, cDC2s and CCR7⁺ DCs in MC38 tumors. Numbers indicate frequencies of cells in each quadrant (mean ± SD, n = 6).

Cohort	Patient ID	Specimen obtained	Original anatomical site	Sex	Smoking history
HNSCC (1)	OP004	Locoregional recurrence	Oropharynx	M	Yes
HNSCC (1)	OP006	Locoregional recurrence	Oropharynx	F	No
HNSCC (1)	L007	Locoregional recurrence	Larynx	F	No
HNSCC (2)	PIO1	Locoregional recurrence	Oral cavity	F	No
HNSCC (2)	PIO6	Distant metastasis	Oropharynx	F	No
HNSCC (2)	PIO9	Primary tumor	Oropharynx	M	No
HNSCC (2)	PIO12	Locoregional recurrence	Oral cavity	M	Yes
HNSCC (2)	PIO11	Distant metastasis	Oropharynx	M	No
HNSCC (2)	PIO15	Distant metastasis	Oropharynx	F	Yes
HNSCC (2)	PIO26	Locoregional recurrence	Oral cavity	F	No
HNSCC (2)	PIO35	Locoregional recurrence	Oropharynx	M	Yes
**Cohort**	**Patient ID**	**Specimen obtained**	**Histotype**	**Sex**	
Endo.	03	Endometrial adenocarcinoma G1	MELF	F	N/A
Endo.	06	Endometrial adenocarcinoma G1	MELF	F	N/A
Endo.	07	Endometrial adenocarcinoma G1	MELF	F	N/A
Endo.	08	Endometrial adenocarcinoma G1	MELF	F	N/A
Endo.	10	Endometrial adenocarcinoma G1	MELF	F	N/A
**Cohort**	**Patient ID**	**Specimen obtained**	**Histotype**	**Sex**	
NSCLC	38	Chen 2024 (DOI: 0.1038/s41590-024-01792-2)	Brain metastasis, carcinoma	F	N/A
NSCLC	43	Chen 2024 (DOI: 0.1038/s41590-024-01792-2)	Lung, adenocarcinoma	F	N/A
NSCLC	67	Chen 2024 (DOI: 0.1038/s41590-024-01792-2)	Brain metastasis, adenocarcinoma	F	N/A
NSCLC	73	Chen 2024 (DOI: 0.1038/s41590-024-01792-2)	Lung, adenocarcinoma	M	N/A

Table S1: Patient sample characteristics (cohorts for spatial analyses of vessel-associated CCR7$^+$ DCs; related to main Figure 1).

Bibliography

1. Bill, R., Wirapati, P., Messemaker, M., Roh, W., Zitti, B., Duval, F., Kiss, M.t., Park, J.C., Saal, T.M., Hoelzl, J., et al. (2023). CXCL9:SPP1 macrophage polarity identifies a network of cellular programs that control human cancers. Science *381*, 515-524. 10.1126/science.ade2292.
2. Chen, J.H., Nieman, L.T., Spurrell, M., Jorgji, V., Elmelech, L., Richieri, P., Xu, K.H., Madhu, R., Parikh, M., Zamora, I., et al. (2024). Human lung cancer harbors spatially organized stem-immunity hubs associated with response to immunotherapy. Nat. Immunol. *25*, 644-658. 10.1038/s41590-024-01792-2.
3. Bassez, A., Vos, H., Dyck, L.V., Floris, G., Arijs, I., Desmedt, C., Boeckx, B., Bempt, M.V., Nevelsteen, I., Lambein, K., et al. (2021). A single-cell map of intratumoral changes during anti-PD1 treatment of patients with breast cancer. Nature Medicine *27*, 820-832. 10.1038/s41591-021-01323-8.
4. https://vizgen.com/human-ffpe-immunooncology-release-roadmap/
5. Kürten, C.H.L., Kulkarni, A., Cillo, A.R., Santos, P.M., Roble, A.K., Onkar, S., Reeder, C., Lang, S., Chen, X., Duvvuri, U., et al. (2021). Investigating immune and non-immune cell interactions in head and neck tumors by single-cell RNA sequencing. Nat. Commun. *12*, 7338. 10.1038/s41467-021-27619-4.
6. Pelka, K., Hofree, M., Chen, J.H., Sarkizova, S., Pirl, J.D., Jorgji, V., Bejnood, A., Dionne, D., Ge, W.H., Xu, K.H., et al. (2021). Spatially organized multicellular immune hubs in human colorectal cancer. Cell *184*, 4734-4752.e4720. 10.1016/j.cell.2021.08.003.
7. Zhang, X., Peng, L., Luo, Y., Zhang, S., Pu, Y., Chen, Y., Guo, W., Yao, J., Shao, M., Fan, W., et al. (2021). Dissecting esophageal squamous-cell carcinoma ecosystem by single-cell transcriptomic analysis. Nat. Commun. *12*, 5291. 10.1038/s41467-021-25539-x.
8. Zilionis, R., Engblom, C., Pfirschke, C., Savova, V., Zemmour, D., Saatcioglu, H.D., Krishnan, I., Maroni, G., Meyerovitz, C.V., Kerwin, C.M., et al. (2019). Single-Cell Transcriptomics of Human and Mouse Lung Cancers Reveals Conserved Myeloid Populations across Individuals and Species. Immunity *50*, 1317-1334.e1310. 10.1016/j.immuni.2019.03.009.
9. Alghamri, M.S., McClellan, B.L., Avvari, R.P., Thalla, R., Carney, S., Hartlage, M.S., Haase, S., Ventosa, M., Taher, A., Kamran, N., et al. (2021). G-CSF secreted by mutant IDH1 glioma stem cells abolishes myeloid cell immunosuppression and enhances the efficacy of immunotherapy. Sci. Adv. *7*, eabh3243. 10.1126/sciadv.abh3243.
10. Murgaski, A., Kiss, M.t., Damme, H.V., Kancheva, D., Vanmeerbeek, I., Keirsse, J., Hadadi, E., Brughmans, J., Arnouk, S.M., Hamouda, A.E.I., et al. (2022). Efficacy of CD40 agonists is mediated by distinct cDC subsets and subverted by suppressive macrophages. Cancer Res. *82*, 3785-3801. 10.1158/0008-5472.can-22-0094.
11. Ramos, R.N., Missolo-Koussou, Y., Gerber-Ferder, Y., Bromley, C.P., Bugatti, M., Nüñez, N.G., Boari, J.T., Richer, W., Menger, L., Denizeau, J., et al. (2022). Tissue-resident FOLR2+ macrophages associate with CD8+ T¬†cell infiltration in human breast cancer. Cell *185*, 1189-1207.e1125. 10.1016/j.cell.2022.02.021.
12. Li, J., Hubisz, M.J., Earlie, E.M., Duran, M.A., Hong, C., Varela, A.A., Lettera, E., Deyell, M., Tavora, B., Havel, J.J., et al. (2023). Non-cell-autonomous cancer progression from chromosomal instability. Nature *620*, 1080-1088. 10.1038/s41586-023-06464-z.
13. Ramirez, C.F.A., Taranto, D., Ando-Kuri, M., Groot, M.H.P.d., Tsouri, E., Huang, Z., Groot, D.d., Kluin, R.J.C., Kloosterman, D.J., Verheij, J., et al. (2024). Cancer cell genetics shaping of the tumor microenvironment reveals myeloid cell-centric exploitable vulnerabilities in hepatocellular carcinoma. Nat. Commun. *15*, 2581. 10.1038/s41467-024-46835-2.
